# Diabetes mortality and trends before 25 years of age: an analysis of the Global Burden of Disease Study 2019

**DOI:** 10.1016/S2213-8587(21)00349-1

**Published:** 2022-03

**Authors:** GBD 2019 Diabetes Mortality Collaborators, Ewerton Cousin, Bruce B Duncan, Caroline Stein, Kanyin Liane Ong, Theo Vos, Cristiana Abbafati, Mohsen Abbasi-Kangevari, Michael Abdelmasseh, Amir Abdoli, Rami Abd-Rabu, Hassan Abolhassani, Eman Abu-Gharbieh, Manfred Mario Kokou Accrombessi, Qorinah Estiningtyas Sakilah Adnani, Muhammad Sohail Afzal, Gina Agarwal, Krishna K Agrawaal, Marcela Agudelo-Botero, Bright Opoku Ahinkorah, Sajjad Ahmad, Tauseef Ahmad, Keivan Ahmadi, Sepideh Ahmadi, Ali Ahmadi, Ali Ahmed, Yusra Ahmed Salih, Wuraola Akande-Sholabi, Tayyaba Akram, Hanadi Al Hamad, Ziyad Al-Aly, Jacqueline Elizabeth Alcalde-Rabanal, Vahid Alipour, Syed Mohamed Aljunid, Rajaa M Al-Raddadi, Nelson Alvis-Guzman, Saeed Amini, Robert Ancuceanu, Tudorel Andrei, Catalina Liliana Andrei, Ranjit Mohan Anjana, Adnan Ansar, Ippazio Cosimo Antonazzo, Benny Antony, Anayochukwu Edward Anyasodor, Jalal Arabloo, Damian Arizmendi, Benedetta Armocida, Anton A Artamonov, Judie Arulappan, Zahra Aryan, Samaneh Asgari, Tahira Ashraf, Thomas Astell-Burt, Prince Atorkey, Maha Moh'd Wahbi Atout, Martin Amogre Ayanore, Ashish D Badiye, Atif Amin Baig, Mohan Bairwa, Jennifer L Baker, Ovidiu Constantin Baltatu, Palash Chandra Banik, Anthony Barnett, Mark Thomaz Ugliara Barone, Francesco Barone-Adesi, Amadou Barrow, Neeraj Bedi, Rebuma Belete, Uzma Iqbal Belgaumi, Arielle Wilder Bell, Derrick A Bennett, Isabela M Bensenor, David Beran, Akshaya Srikanth Bhagavathula, Sonu Bhaskar, Krittika Bhattacharyya, Vijayalakshmi S Bhojaraja, Ali Bijani, Boris Bikbov, Setognal Birara, Virginia Bodolica, Aime Bonny, Hermann Brenner, Nikolay Ivanovich Briko, Zahid A Butt, Florentino Luciano Caetano dos Santos, Luis Alberto Cámera, Ismael R Campos-Nonato, Yin Cao, Chao Cao, Ester Cerin, Promit Ananyo Chakraborty, Joht Singh Chandan, Vijay Kumar Chattu, Simiao Chen, Jee-Young Jasmine Choi, Sonali Gajanan Choudhari, Enayet Karim Chowdhury, Dinh-Toi Chu, Barbara Corso, Omid Dadras, Xiaochen Dai, Albertino Antonio Moura Damasceno, Lalit Dandona, Rakhi Dandona, Claudio Alberto Dávila-Cervantes, Jan-Walter De Neve, Edgar Denova-Gutiérrez, Deepak Dhamnetiya, Daniel Diaz, Sanam Ebtehaj, Hisham Atan Edinur, Sahar Eftekharzadeh, Iman El Sayed, Islam Y Elgendy, Muhammed Elhadi, Mohamed A Elmonem, Mohammed Faisaluddin, Umar Farooque, Xiaoqi Feng, Eduarda Fernandes, Florian Fischer, David Flood, Marisa Freitas, Peter Andras Gaal, Mohamed M Gad, Piyada Gaewkhiew, Lemma Getacher, Mansour Ghafourifard, Reza Ghanei Gheshlagh, Ahmad Ghashghaee, Nermin Ghith, Ghozali Ghozali, Paramjit Singh Gill, Ibrahim Abdelmageed Ginawi, Ekaterina Vladimirovna Glushkova, Mahaveer Golechha, Sameer Vali Gopalani, Rafael Alves Guimarães, Rajat Das Gupta, Rajeev Gupta, Vivek Kumar Gupta, Veer Bala Gupta, Sapna Gupta, Tesfa Dejenie Habtewold, Nima Hafezi-Nejad, Rabih Halwani, Asif Hanif, Graeme J Hankey, Shafiul Haque, Ahmed I Hasaballah, Syed Shahzad Hasan, Abdiwahab Hashi, Soheil Hassanipour, Simon I Hay, Khezar Hayat, Mohammad Heidari, Mohammad Bellal Hossain Hossain, Sahadat Hossain, Mostafa Hosseini, Soodabeh Hoveidamanesh, Junjie Huang, Ayesha Humayun, Rabia Hussain, Bing-Fang Hwang, Segun Emmanuel Ibitoye, Kevin S Ikuta, Leeberk Raja Inbaraj, Usman Iqbal, Md Shariful Islam, Sheikh Mohammed Shariful Islam, Rakibul M Islam, Nahlah Elkudssiah Ismail, Gaetano Isola, Ramaiah Itumalla, Masao Iwagami, Ihoghosa Osamuyi Iyamu, Mohammad Ali Jahani, Mihajlo Jakovljevic, Ranil Jayawardena, Ravi Prakash Jha, Oommen John, Jost B Jonas, Tamas Joo, Ali Kabir, Rohollah Kalhor, Ashwin Kamath, Tanuj Kanchan, Himal Kandel, Neeti Kapoor, Gbenga A Kayode, Sewnet Adem Kebede, Pedram Keshavarz, Mohammad Keykhaei, Yousef Saleh Khader, Himanshu Khajuria, Moien AB Khan, Md Nuruzzaman Khan, Maseer Khan, Amir M Khater, Tawfik Ahmed Muthafer Khoja, Jagdish Khubchandani, Min Seo Kim, Yun Jin Kim, Ruth W Kimokoti, Sezer Kisa, Adnan Kisa, Mika Kivimäki, Vladimir Andreevich Korshunov, Oleksii Korzh, Ai Koyanagi, Kewal Krishan, Barthelemy Kuate Defo, G Anil Kumar, Nithin Kumar, Dian Kusuma, Carlo La Vecchia, Ben Lacey, Anders O Larsson, Savita Lasrado, Wei-Chen Lee, Chiachi Bonnie Lee, Paul H Lee, Shaun Wen Huey Lee, Ming-Chieh Li, Stephen S Lim, Lee-Ling Lim, Giancarlo Lucchetti, Azeem Majeed, Ahmad Azam Malik, Borhan Mansouri, Lorenzo Giovanni Mantovani, Santi Martini, Prashant Mathur, Colm McAlinden, Nafiul Mehedi, Teferi Mekonnen, Ritesh G Menezes, Amanual Getnet Mersha, Junmei Miao Jonasson, Tomasz Miazgowski, Irmina Maria Michalek, Andreea Mirica, Erkin M Mirrakhimov, Agha Zeeshan Mirza, Prasanna Mithra, Abdollah Mohammadian-Hafshejani, Reza Mohammadpourhodki, Arif Mohammed, Ali H Mokdad, Mariam Molokhia, Lorenzo Monasta, Mohammad Ali Moni, Farhad Moradpour, Rahmatollah Moradzadeh, Ebrahim Mostafavi, Ulrich Otto Mueller, Christopher J L Murray, Ahmad Mustafa, Gabriele Nagel, Vinay Nangia, Atta Abbas Naqvi, Biswa Prakash Nayak, Javad Nazari, Rawlance Ndejjo, Ruxandra Irina Negoi, Sandhya Neupane Kandel, Cuong Tat Nguyen, Huong Lan Thi Nguyen, Jean Jacques Noubiap, Christoph Nowak, Bogdan Oancea, Oluwakemi Ololade Odukoya, Ayodipupo Sikiru Oguntade, Temitope T Ojo, Andrew T Olagunju, Obinna E Onwujekwe, Alberto Ortiz, Mayowa O Owolabi, Raffaele Palladino, Songhomitra Panda-Jonas, Seithikurippu R Pandi-Perumal, Shahina Pardhan, Tarang Parekh, Mojtaba Parvizi, Veincent Christian Filipino Pepito, Arokiasamy Perianayagam, Ionela-Roxana Petcu, Manju Pilania, Vivek Podder, Roman V Polibin, Maarten J Postma, Akila Prashant, Navid Rabiee, Mohammad Rabiee, Vafa Rahimi-Movaghar, Muhammad Aziz Rahman, Md. Mosfequr Rahman, Mosiur Rahman, Setyaningrum Rahmawaty, Nazanin Rajai, Pradhum Ram, Juwel Rana, Kamal Ranabhat, Priyanga Ranasinghe, Chythra R Rao, Satish Rao, Salman Rawaf, David Laith Rawaf, Lal Rawal, Andre M N Renzaho, Nima Rezaei, Aziz Rezapour, Seyed Mohammad Riahi, Daniela Ribeiro, Jefferson Antonio Buendia Rodriguez, Leonardo Roever, Peter Rohloff, Godfrey M Rwegerera, Paul MacDaragh Ryan, Maha Mohamed Saber-Ayad, Siamak Sabour, Basema Saddik, Sahar Saeedi Moghaddam, Amirhossein Sahebkar, Harihar Sahoo, KM Saif-Ur-Rahman, Hamideh Salimzadeh, Mehrnoosh Samaei, Juan Sanabria, Milena M Santric-Milicevic, Brijesh Sathian, Thirunavukkarasu Sathish, Markus P Schlaich, Abdul-Aziz Seidu, Mario Šekerija, Nachimuthu Senthil Kumar, Allen Seylani, Masood Ali Shaikh, Hina Shamshad, Md Shajedur Rahman Shawon, Sara Sheikhbahaei, Jeevan K Shetty, Rahman Shiri, K M Shivakumar, Kerem Shuval, Jasvinder A Singh, Ambrish Singh, Valentin Yurievich Skryabin, Anna Aleksandrovna Skryabina, Ahmad Sofi-Mahmudi, Amin Soheili, Jing Sun, Viktória Szerencsés, Miklós Szócska, Rafael Tabarés-Seisdedos, Hooman Tadbiri, Eyayou Girma Tadesse, Md. Tariqujjaman, Kavumpurathu Raman Thankappan, Rekha Thapar, Nihal Thomas, Binod Timalsina, Ruoyan Tobe-Gai, Marcello Tonelli, Marcos Roberto Tovani-Palone, Bach Xuan Tran, Jaya Prasad Tripathy, Lorainne Tudor Car, Biruk Shalmeno Tusa, Riaz Uddin, Era Upadhyay, Sahel Valadan Tahbaz, Pascual R Valdez, Tommi Juhani Vasankari, Madhur Verma, Victor E Villalobos-Daniel, Sergey Konstantinovitch Vladimirov, Bay Vo, Giang Thu Vu, Rade Vukovic, Yasir Waheed, Richard G Wamai, Andrea Werdecker, Nuwan Darshana Wickramasinghe, Andrea Sylvia Winkler, Befikadu Legesse Wubishet, Xiaoyue Xu, Suowen Xu, Seyed Hossein Yahyazadeh Jabbari, Hiroshi Yatsuya, Sanni Yaya, Taklo Simeneh Yazie Yazie, Siyan Yi, Naohiro Yonemoto, Ismaeel Yunusa, Siddhesh Zadey, Sojib Bin Zaman, Maryam Zamanian, Nelson Zamora, Mikhail Sergeevich Zastrozhin, Anasthasia Zastrozhina, Zhi-Jiang Zhang, Chenwen Zhong, Mohammad Zmaili, Alimuddin Zumla, Mohsen Naghavi, Maria Inês Schmidt

## Abstract

**Background:**

Diabetes, particularly type 1 diabetes, at younger ages can be a largely preventable cause of death with the correct health care and services. We aimed to evaluate diabetes mortality and trends at ages younger than 25 years globally using data from the Global Burden of Diseases, Injuries, and Risk Factors Study (GBD) 2019.

**Methods:**

We used estimates of GBD 2019 to calculate international diabetes mortality at ages younger than 25 years in 1990 and 2019. Data sources for causes of death were obtained from vital registration systems, verbal autopsies, and other surveillance systems for 1990–2019. We estimated death rates for each location using the GBD Cause of Death Ensemble model. We analysed the association of age-standardised death rates per 100 000 population with the Socio-demographic Index (SDI) and a measure of universal health coverage (UHC) and described the variability within SDI quintiles. We present estimates with their 95% uncertainty intervals.

**Findings:**

In 2019, 16 300 (95% uncertainty interval 14 200 to 18 900) global deaths due to diabetes (type 1 and 2 combined) occurred in people younger than 25 years and 73·7% (68·3 to 77·4) were classified as due to type 1 diabetes. The age-standardised death rate was 0·50 (0·44 to 0·58) per 100 000 population, and 15 900 (97·5%) of these deaths occurred in low to high-middle SDI countries. The rate was 0·13 (0·12 to 0·14) per 100 000 population in the high SDI quintile, 0·60 (0·51 to 0·70) per 100 000 population in the low-middle SDI quintile, and 0·71 (0·60 to 0·86) per 100 000 population in the low SDI quintile. Within SDI quintiles, we observed large variability in rates across countries, in part explained by the extent of UHC (*r*^2^=0·62). From 1990 to 2019, age-standardised death rates decreased globally by 17·0% (−28·4 to −2·9) for all diabetes, and by 21·0% (–33·0 to −5·9) when considering only type 1 diabetes. However, the low SDI quintile had the lowest decline for both all diabetes (−13·6% [–28·4 to 3·4]) and for type 1 diabetes (−13·6% [–29·3 to 8·9]).

**Interpretation:**

Decreasing diabetes mortality at ages younger than 25 years remains an important challenge, especially in low and low-middle SDI countries. Inadequate diagnosis and treatment of diabetes is likely to be major contributor to these early deaths, highlighting the urgent need to provide better access to insulin and basic diabetes education and care. This mortality metric, derived from readily available and frequently updated GBD data, can help to monitor preventable diabetes-related deaths over time globally, aligned with the UN's Sustainable Development Targets, and serve as an indicator of the adequacy of basic diabetes care for type 1 and type 2 diabetes across nations.

**Funding:**

Bill & Melinda Gates Foundation.

## Introduction

Diabetes has been identified by the UN and WHO as one of the five priority non-communicable diseases (NCDs) in their Action Plan to confront the NCDs challenge.^1^, ^2^ Prevention and management of the chronic complications in patients with diabetes involve complex, long-lasting, and costly endeavours.^3^

By contrast, deaths due to acute complications (ie, diabetic ketoacidosis, hyperosmolar coma, and severe hypoglycaemia), early-onset renal failure, and acute infections can be prevented through a minimal core set of actions. These include the provision of affordable insulin,^4^ access to health care (including glucose monitoring and promptly available services for acute decompensation), and health education (including rapid recognition and detection of type 1 diabetes and ketoacidosis).^5^ Since contemporary health care in high-income countries^6^, ^7^, ^8^ has proven to be effective in reducing mortality due to these complications it is reasonable to assume that major reductions could be seen globally if better care, including the availability of affordable insulin, were more widely provided.

Many low-income and middle-income countries (LMICs) have made considerable progress in the provision of a minimal core of actions to prevent mortality due to acute complications of diabetes. For example, Brazil observed a decrease of 74·5% in deaths due to acute complications in people younger than 40 years from 1991 to 2010 following the implementation of the National Health System, which greatly increased access to care for patients with diabetes, including the provision of free insulin.^9^

Since mortality data are the most available health statistics worldwide, they can be used to track levels and trends in basic care for diabetes. However, deaths due to diabetes (International Classification of Diseases, revision 10 [ICD-10], codes E10–E14) are frequently further classified vaguely as due to multiple (.7) or unspecified (.8) complications, or without complications (.9), making a direct assessment of trends in deaths due to acute complications difficult.^10^ For example, from 1996 to 2011 in Brazil, 83·6% of deaths, after removing those due to renal disease (.2) and peripheral arterial disease (.5), were coded with uninformative .7 to .9 codes.^10^ However, because mortality in people younger than 25 years is probably mostly due to acute complications,^11^ restricting the evaluation to this age range permits a focus on avoidable causes of death.

**Research in context**

**Evidence before this study**

Deaths due to acute complications of diabetes such as diabetic ketoacidosis, hyperosmolar coma, and severe hypoglycaemia, as well as due to early manifestation of diabetic kidney disease and acute infections, can largely be avoided with adequately functioning basic health care, including ready access to insulin. This has mostly been accomplished in high-income countries. Direct monitoring of progress on this cause of death globally is currently not feasible because details on the type of diabetes and cause of death are frequently incomplete, which makes it difficult to track and reveal disparities in avoidable causes of death related to diabetes around the world. We searched PubMed for research articles published up to Oct 13, 2021, using the terms [“diabetes” AND “mortality” AND (“child” OR “adolescent” OR “youth”)]. No language restriction was applied. This search revealed few studies evaluating nationwide diabetes mortality in the initial decades of life, and none considering diabetes type.

**Added value of this study**

The Global Burden of Diseases, Injuries, and Risk Factors Study (GBD) collates mortality data of nations around the world, adjusting for incompleteness of death registration and ill-defined causes of death. To produce more accurate estimates for countries with poor data, GBD applies modelling techniques which take advantage of findings from countries with good data. Based on GBD 2019 estimates, we assessed levels and trends of age-standardised diabetes death rates at ages younger than 25 years, considering it as a metric of potential avoidable mortality from diabetes that can be used for comparisons by location and over time. Trends across the world revealed progress in decreasing these deaths from 1990 to 2019 at all levels of development, as indicated by the GBD's Socio-demographic Index. Countries with a lower level of development had lesser declines and much higher mortality in 2019 than those with a higher level of development. However, even at similar levels of development mortality varied widely across countries, in great part related to the level of universal health coverage. Globally, most of the deaths were due to type 1 diabetes. The death rate due to type 2 diabetes at ages younger than 25 years showed less progress over the period.

**Implications of all the available evidence**

Diabetes-related death among people younger than 25 years, principally due to incomplete or inadequate access to basic health care for type 1 diabetes, can be largely avoided with universal access to insulin and basic diabetes care. However, the task of creating such access is far from accomplished globally. Although disparities were largely related to levels of development of a country, accessibility of health care was also important. The metric of age-standardised mortality due to diabetes at ages younger than 25 years, derived from readily available and frequently updated GBD data, can help to monitor diabetes-related potentially preventable deaths over time globally, aligned with the UN's Sustainable Development Target 3.8 of achieving “universal health coverage, including financial risk protection, access to quality essential health-care services, and access to safe, effective, quality, and affordable essential medicines and vaccines for all”. The metric mostly reflects potentially avoidable deaths due to type 1 diabetes in the young but also those due to early-onset type 2 diabetes, thus helping to give voice to those advocating better care for diabetes across the globe.

The Global Burden of Diseases, Injuries, and Risk Factors Study (GBD), which systematically collects mortality data, offers estimates of mortality rates by age group and disease for countries around the world.^12^ With the goal of developing a simple and up-to-date indicator of potentially preventable diabetes mortality, our objective was to evaluate diabetes mortality in people younger than 25 years and its trends using readily available GBD 2019 data on countries across the world.

## Methods

### Overview and definition

We used estimates from the GBD 2019 study. The GBD applies a standard methodological approach to generate estimates for mortality and causes of death for diseases for 204 countries and territories. We restricted our country-specific analyses to those with a total population of 1 million or more people in 2019 to minimise the higher variability present in the 45 countries with smaller populations. Estimates for all locations can be found in the appendix (pp 45–52).

We defined deaths occurring at ages younger than 25 years as due to type 1 and type 2 diabetes and due to chronic kidney disease resulting from diabetes based on ICD-9 and ICD‑10 codes.

The general methods used to generate these estimates are described in the appendix (pp 5–31), which also includes references to the methodology used in additional GBD publications, which provide further detail. Data sources for causes of death were obtained from vital registration systems, verbal autopsies, and other surveillance systems for 1990–2019.^12^, ^13^ Data inputs used to generate the estimates are available on the Global Health Data exchange website. The appendix (p 13) shows the quality of the national vital registration data over the period 2010–18. An explanation on how to assess the data quality is also provided in the appendix (p 12).

### Mortality estimation

Mortality estimates were generated through standardisation of input data and mapping of ICD-10 (and equivalent ICD-9 codes) to type 1 diabetes (ICD-10 codes E10–E10.11, E10.3–E10.9, and P70.2), type 2 diabetes (E11–E11.1 and E11.3–E11.9), and chronic kidney disease due to each type of diabetes (E10.2 for type 1 and E11.2 for type 2). Age-sex splitting was then performed for data from sources providing only overall summaries. After this process, intermediate or poorly defined ICD codes were redistributed to plausible causes using regression or proportion redistribution methods.^12^ Deaths reported as due to other or unspecified types of diabetes (E12–E14) were redistributed to type 1 or type 2 diabetes using regression modelling. To estimate the causes of death, GBD uses the Cause of Death Ensemble model (CODEm), which combines results from different statistical models weighted on the basis of their out-of-sample predictive validity.^12^ The estimate for each cause of death is the mean of 1000 draws from the set best performing models. 95% uncertainty intervals (UIs) reflect the 25th and 975th values of these 1000 draws, and were calculated for all estimates. CODEm was used to model diabetes overall, both types of diabetes, and chronic kidney disease. However, the distribution of chronic kidney disease deaths due to diabetes into separate type 1 and type 2 diabetes categories was performed with DisMod-MR 2.1, which permits adjustment based on the prevalence of each type. For estimation of deaths due to overall diabetes, the GBD applies two distinct models, one for ages younger than 15 years (calculating deaths assumed to be due only to type 1 diabetes), and the other for ages 15 years and older, representing deaths due to all types of diabetes.^12^ For ages 0–14 years, the covariates used in modelling were Healthcare Access and Quality (HAQ) Index, education years per capita, age-standardised fertility rate, geographic latitude, age-standardised underweight (weight-for-age) summary exposure variable, percentage of births occurring in women older than 35 years, percentage of births occurring in women older than 40 years, Socio-demographic Index (SDI), age-standardised stunting (height-for-age) summary exposure variable, and mean birthweight. For ages 15 years and older, the covariates were age-standardised mean fasting plasma glucose (mmol/L), age-standardised prevalence of diabetes, education years per capita, lag-distributed income per capita, mean BMI, mean cholesterol, mean systolic blood pressure, prevalence of obesity, age-specific and sex-specific summary exposure variable for low fruit intake, energy-adjusted grams of sugar, age-specific and sex-specific summary exposure variable for low vegetable intake, HAQ Index, and age-specific and sex-specific summary exposure variable for alcohol use.

For all models generated, GBD then applies a cause of death correction procedure that scales deaths from individual causes to match all-cause mortality for each sex-year-location, as derived from demographic analyses. Further details on these procedures can be found elsewhere.^12^, ^13^ For this study, we aggregated deaths from diabetes and from chronic kidney disease due to diabetes in ICD-10. Death rates per 100 000 population were age standardised using the direct method and the GBD standard population.^13^

### SDI and universal health coverage index, and diabetes prevalence

The SDI is a composite indicator of development status derived from the total fertility rate in women younger than 25 years, mean education for those 15 years or older, and lag-distributed income per capita,^13^ as detailed in the appendix (p 32). We expressed this index for 204 countries and territories, and stratified it into quintiles to explore the difference in age-standardised death rates due to diabetes between countries at different levels of development. We provide a list of countries by SDI quintile and identify the SDI quintile of each country in a map (appendix pp 40–44, 53).

GBD's universal health coverage (UHC) index was constructed by mapping 23 effective coverage indicators against five health service domains, then weighting each indicator relative to its associated potential health gains.^14^ We analysed the association between the UHC effective coverage index and age-standardised death rates by linear regression and present *r*^2^ as a measure of how much of the variance in diabetes mortality in people younger than 25 years is explained by the UHC index.

We similarly analysed the association between age-standardised prevalence of diabetes and mortality. Prevalence of diabetes was estimated from surveys of glycaemic values or use of diabetes medication, and for individuals younger than 15 years also from diabetes registries or hospital records. Detailed description of prevalence estimation is found elsewhere.^12^

Analyses in this Article were conducted with Python (version 3.6.2), Stata (version 13), and R (versions 3.5.0 and 3.6.0).

### Role of the funding source

The funder of the study had no role in study design, data collection, data analysis, data interpretation, or writing of the report.

## Results

### Mortality due to diabetes

In 2019, 16 300 (95% UI 14 200–18 900) deaths were due to diabetes (types 1 and 2 combined) in people younger than 25 years globally, with 15 900 (97·5%) occurring in low to high-middle SDI countries. 415 (390–443) individuals younger than 25 years died from diabetes in 2019 in high SDI countries compared to 4860 (4070–5900) in the low SDI countries and 5300 (4510–6200) in the low-middle SDI countries. Among global deaths, 73·7% (68·3–77·4) were classified as due to type 1 diabetes, and the remainder as due to type 2 diabetes. Chronic kidney disease due to either type of diabetes was responsible for 16·5% (9·8–24·6) of these deaths (varying between 4·9% [2·5–8·5] in the high SDI quintile and 29·1% [18·8–39·8] in the middle SDI quintile). At ages younger than 25 years, diabetes was a rare cause of death, with 16 300 (0·24%) deaths of 6 912 000 total deaths from all causes globally, varying from 4860 (0·15%) of 3 259 000 deaths in the low SDI quintile to 4710 (0·42%) of 1 133 000 deaths in the middle SDI quintile. Data for all-cause mortality are from GBD 2019 results (appendix pp 5–6). The age-standardised death rate in 2019 was 0·50 (0·44–0·58) per 100 000 population, varying from 0·13 (0·12–0·14) per 100 000 population in the high SDI quintile to 0·60 (0·51–0·70) per 100 000 population in the low-middle SDI quintile and 0·71 (0·60–0·86) per 100 000 population in the low SDI quintile (table 1). Age-standardised death rates decreased by 17·0% (−28·4 to −2·9) globally from 1990 to 2019, ranging from −13·6% (−28·4 to 3·4) in the low SDI quintile to −44·5% (−52·0 to −36·5) in the high-middle SDI quintile (table 1).

**Table 1:** Comparison of mortality due to diabetes at ages younger than 25 years and contribution of CKD according to the SDI

	**Deaths, 2019**				**Age–standardised death rate**				
	Number	Percentage due to type 1 diabetes	Percentage due to CKD*	Percentage of total deaths†	Per 100 000, 1990	Per 100 000, 2019	All diabetes percentage change, 1990–2019	Type 1 diabetes percentage change, 1990–2019	Type 2 diabetes percentage change, 1990–2019
Global	16 300 (14 200 to 18 900)	73·7% (68·3 to 77·4)	16·5% (9·8 to 24·6)	0·24% (0·21 to 0·27)	0·60 (0·51 to 0·69)	0·50 (0·44 to 0·58)	−17·0% (−28·4 to −2·9)	−21·0% (−33·0 to −5·9)	−2·5% (−17·7 to 15·8)
Low SDI	4860 (4070 to 5900)	79·3% (74·3 to 82·5)	7·8% (4·1 to 13·3)	0·15% (0·13 to 0·17)	0·83 (0·70 to 0·95)	0·71 (0·60 to 0·86)	−13·6% (−28·4 to 3·4)	−13·6% (−29·3 to 8·9)	−13·7% (−32·2 to 11·1)
Low–middle SDI	5300 (4510 to 6200)	73·7% (66·5 to 77·8)	13·3% (7·6 to 20·7)	0·25% (0·22 to 0·29)	0·71 (0·60 to 0·84)	0·60 (0·51 to 0·70)	−15·4% (−30·9 to 2·6)	−18·4% (−35·9 to 2·9)	−5·0% (−28·9 to 23·9)
Middle SDI	4710 (4000 to 5640)	67·5% (60·9 to 72·6)	29·1% (18·8 to 39·8)	0·42% (0·35 to 0·50)	0·70 (0·56 to 0·83)	0·48 (0·41 to 0·57)	−31·3% (−40·2 to −15·4)	−38·6% (−47·4 to −21·6)	−7·2% (−20·0 to 9·0)
High–middle SDI	1000 (878 to 1140)	72·9% (67·8 to 76·4)	20·8% (12·8 to 29·8)	0·35% (0·31 to 0·40)	0·39 (0·34 to 0·45)	0·22 (0·19 to 0·25)	−44·5% (−52·0 to −36·5)	−48·8% (−56·3 to −41·5)	−26·5% (−37·2 to −11·2)
High SDI	415 (390 to 443)	83·1% (78·2 to 85·2)	4·9% (2·5 to 8·5)	0·35% (0·33 to 0·37)	0·19 (0·17 to 0·20)	0·13 (0·12 to 0·14)	−29·4% (−33·3 to −25·4)	−29·6% (−33·7 to −25·4)	−28·7% (−37·1 to −15·4)

Data in parentheses are 95% uncertainty intervals. CKD=chronic kidney disease· SDI=Socio-demographic Index.

* CKD deaths due to type 1 and type 2 diabetes; note that percentages due to type 1 diabetes and those due to CKD are not mutually exclusive.

† Number of deaths due to diabetes at ages younger than 25 years divided by the total of deaths from all causes at ages younger than 25 years.

The appendix presents trends in age-standardised death rates due to diabetes overall by SDI for ages younger than 25 years, from 1990 to 2019 (appendix p 54), as well as data for age-standardised deaths rate, percentage change in rates from 1990 to 2019, percentage of total deaths, number of deaths, and population for all countries grouped into SDI quintiles (appendix pp 45–52). Additionally, age-specific mortality before the age of 15 years in 2019**,** as expected, was very low for all SDI quintiles, except for the low SDI quintile (appendix p 55). Rates then increased, particularly in the low to middle SDI quintiles. At the age range of 20–24 years, rates in the low SDI were almost 3·5 times those of the high SDI quintile (appendix p 55).

### Age-standardised death rates by type of diabetes

Because most deaths before the age of 25 years were due to type 1 diabetes, declines seen for this diabetes type resembled those seen for all cases. By contrast, declines for type 2 diabetes were generally smaller globally (−2·5 % [95% UI −17·7 to 15·8]), although variable across SDI quintiles (eg, −28·7% [–37·1 to −15·4] in high SDI countries; table 1).

**Figure 1:**
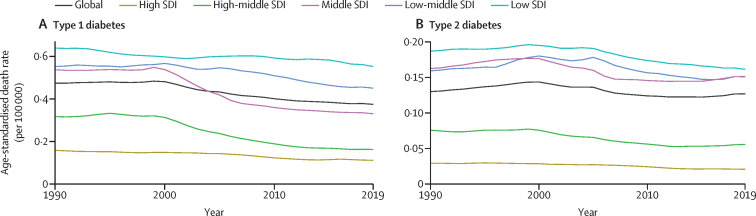
Trends in age-standardised death rates due to type 1 diabetes (A) and type 2 diabetes (B) at ages younger than 25 years, 1990–2019, by SDI quintiles SDI=Socio-demographic Index.

For type 1 diabetes (figure 1A), the age-standardised death rate decreased by 21·0% (95% UI −33·0 to −5·9) globally when considering only type 1 diabetes, and the greatest decreases from 1990 to 2019, starting at about year 2000, were observed for the high-middle quintile (−48·8% [–56·3 to −41·5]) and middle SDI quintile (−38·6% [–47·4 to −21·6]). In 2019, the high-middle quintile approached the rate of high SDI countries. Lesser decreases were seen in low-middle and low SDI quintiles, leaving them with high rates in 2019. For type 2 diabetes (figure 1B), declines were generally small. Ranking of SDI quintiles by their type 2 diabetes age-standardised death rates in 2019 showed a similar pattern to that seen for type 1 diabetes, except for the middle SDI quintile, the rate of which equalled that of the low-middle SDI quintile (figure 1B).

### Differences in age-standardised death rates by location

**Figure 2:**
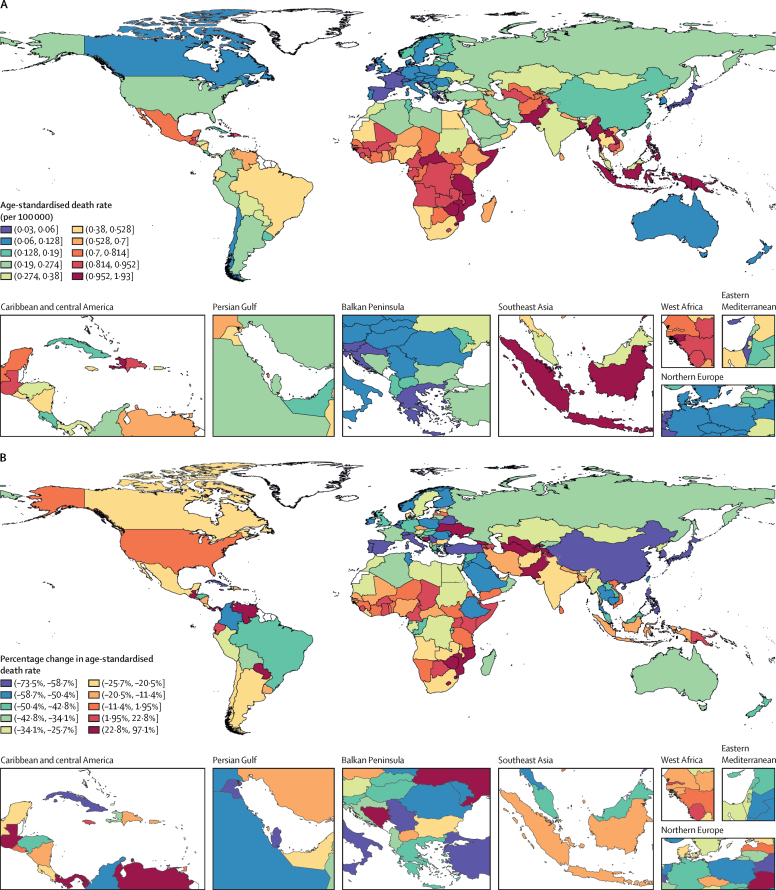
Age-standardised mortality due to diabetes at ages younger than 25 years (A) Age-standardised death rate per 100 000 population in 2019. (B) Percentage change in age-standardised death rate from 1990 to 2019. Countries with total population of less than 1 million people are excluded.

Important variation in age-standardised death rates due to diabetes before the age of 25 years in 2019 was seen globally (figure 2A). Many countries in sub-Saharan Africa, the Caribbean, and southeast Asia, as well as a cluster of countries in central Asia together with neighbouring Afghanistan and Pakistan, had the highest age-standardised death rates. By contrast, high-income countries of western Europe, Australasia, and Asia Pacific, as well as Chile and Canada, had the lowest age-standardised death rates.

Progress in decreasing this mortality rate from 1990 to 2019 has also varied considerably. Although age-standardised death rates in some countries increased, rates decreased in most countries, frequently by more than half (figure 2B). Many countries in central Asia, Oceania, Latin America and the Caribbean, and eastern, western, and southern sub-Saharan Africa had high increases, and many from Europe, high-income Asia Pacific, and east and southeast Asia had large decreases.

The three countries with the highest age-standardised diabetes death rates in 2019 across all SDI quintiles were Myanmar (1·93 [95% UI 1·30–2·68] per 100 000 population; table 2), Papua New Guinea (1·78 [1·29–2·39] per 100 000 population; table 2), and Haiti (1·57 [1·14–2·10] per 100 000 population; appendix p 48). The three countries with lowest age-standardised diabetes death rates in 2019 regardless of SDI index were Cyprus (0·03 [0·02–0·04] per 100 000 population; table 2), Slovenia (0·03 [0·03–0·04] per 100 000 population; appendix p 46), and Switzerland (0·03 [0·03–0·04] per 100 000 population; appendix p 47). Frequently, the highest rate in each SDI quintile was approximately ten times that of the lowest (table 2). A similar mortality pattern was seen globally when analyses included only type 1 diabetes deaths (appendix p 56).

**Table 2:** Highest and lowest age-standardised death rates due to diabetes at ages younger than 25 years in 2019, and most and least favourable percentage changes from 1990 to 2019, by SDI quintile

	**Age-standardised death rate per 100 000, 2019**		**Percentage change, 1990–2019**	
	Highest	Lowest	Least favourable	Most favourable
Low SDI	1·78 (1·29 to 2·39; Papua New Guinea)	0·27 (0·13 to 0·42; Yemen)	91·4% (41·1 to 146·0; Pakistan)	−52·2% (−64·8 to −35·8; Ethiopia)
Low-middle SDI	1·93 (1·30 to 2·68; Myanmar)	0·23 (0·19 to 0·28; Kyrgyzstan)	84·8% (18·1 to 158·2; Guatemala)	−51·1% (−66·2 to −27·0; Cambodia)
Middle SDI	1·35 (1·07 to 1·70; Philippines)	0·15 (0·11 to 0·19; Cuba)	96·3% (57·0 to 137·7; Uzbekistan)	−64·8% (−72·8 to −54·6; Cuba)
High-middle SDI	1·31 (0·98 to 1·74; Mauritius)	0·04 (0·03 to 0·05; Spain)	97·1% (51·0 to 151·2; Mauritius)	−73·1% (−82·8 to −60·9; Turkey)
High SDI	0·45 (0·34 to 0·62; Kuwait)	0·03 (0·02 to 0·04; Cyprus)	−3·5% (−10·5 to 2·0; USA)	−73·5% (−79·6 to −63·2; Singapore)

Data in the parentheses are 95% uncertainty intervals and countries. SDI=Socio-demographic Index.

### Differences in age-standardised death rates by SDI and UHC indices

Variability was also seen when considering the extremes of changes within each SDI quintile. For example, in the high SDI quintile, the age-standardised death rate decreased by 73·5% (95% UI −79·6 to −63·2) for Singapore versus 3·5% (−10·5 to 2·0) for the USA; within the low SDI quintile, the rate increased by 91·4% (41·1 to 146·0) for Pakistan and decreased by 52·2% (−64·8 to −35·8) for Ethiopia (table 2).

**Figure 3:**
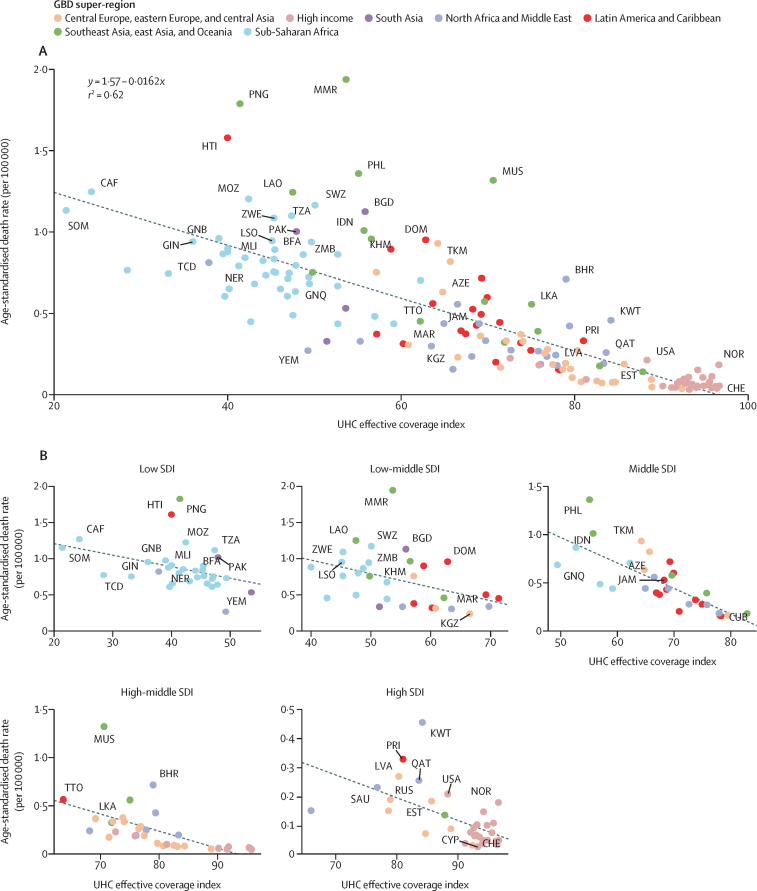
Scatterplot of age-standardised diabetes death rate at ages younger than 25 years by UHC index in 2019 globally (A) and by SDI quintile (B) The diagonal line is the death rate at each UHC index as estimated by linear regression. Country super-regions are indicated by colours. UHC=universal health coverage.

Death rates in 2019 were unrelated to age-standardised diabetes prevalence at ages younger than 25 years (*r*^2^=0·0004; data not shown). Universal health coverage was inversely associated with age-standardised death rates per 100 000 population explaining 62% of total variance (*r*^2^=0·62; figure 3A). An inverse association was also present within each individual SDI stratum (figure 3B).

## Discussion

From 1990 to 2019, we observed general progress in decreasing mortality due to diabetes at ages younger than 25 years, mostly related to type 1 diabetes. However, large disparities persisted across countries, with age-standardised mortality of low and low-middle SDI countries being approximately five times that of high SDI countries and varying more than ten times across countries within SDI quintiles. UHC explains an important part of the variability in rates and highlights the role of access to care in reducing disparities in mortality in this age group.

Deaths due to acute complications of type 1 diabetes, although usually avoidable, have been rarely used as an indicator of potentially preventable deaths, owing to poor specification on death certificates. By restricting our analyses to ages younger than 25 years, a range in which most deaths are among cases of type 1 diabetes and presumably mostly due to acute complications, this derived indicator minimises the problem of poor reporting in vital registration. Chronic kidney disease, as here reported, represents 16·5% of all diabetes deaths before the age of 25 years. As this cause of death at early ages is likely to be related to extremely poor glycaemic control, which can be prevented through basic diabetes care, it can logically be considered within this metric of avoidable deaths. Of note, acute infections associated with poor glycaemic control, another potentially avoidable cause of death among people with diabetes at ages younger than 25 years,^15^, ^16^, ^17^, ^18^ will not be captured by the metric as they are not assigned to diabetes on the death certificate.

Deaths due to chronic complications at ages younger than 25 years also occur in people with early-onset type 2 diabetes, as type 2 diabetes in the young has been recognised as a more severe form of disease than diabetes presenting at older ages.^19^ As the epidemic of type 2 diabetes grows, type 2 diabetes in the young is posing new challenges to health care. The small and variable progress in decreasing early deaths due to type 2 diabetes described here highlights the need to monitor this mortality in the years to come.

Our findings of low mortality rates at ages younger than 25 years in high SDI countries attests to the amenability of these deaths and supports the importance of access to diabetes medications and basic diabetes care. Protocols have been successfully implemented in high-income countries over the years.^20^ However, many LMICs, while striving to adjust to rapid and incomplete epidemiological transitions, have faced additional obstacles, such as limited technical expertise and political and economic instability, complicating their implementation of similar protocols. The rapid decrease in deaths due to acute complications in Brazil,^9^ a country in the middle SDI quintile, with increasingly adequate and accessible primary care and the provision of free insulin, highlights the preventability of such deaths in settings with fewer resources.

Almost 22 million people were living with type 1 diabetes in 2019,^12^ needing insulin daily. Insulin access and prices vary widely across countries,^21^ and limited government expenditures for health^22^ hinder the creation of universal care systems in many LMICs. In settings where individuals are required to pay out-of-pocket for all or part of their diabetes care, which includes insulin as well as syringes, blood glucose meters, and the necessary health education, cost can make insulin treatment unaffordable.^4^ This is particularly important in low-income countries, in which insulin prices are frequently higher than in middle-income and high-income countries.^23^, ^24^

By analysing diabetes mortality at ages younger than 25 years, we have mapped areas of the world that need greater action to prevent these avoidable early deaths. Among these areas are sub-Saharan Africa, parts of central and southeast Asia, Oceania, and Latin America and the Caribbean. Countries in these locations have high age-standardised death rates due to diabetes, in comparison to those from the high SDI quintile, and in some, rates are increasing.^12^ Although our analyses for these countries frequently depend on modelling, we can postulate that inadequate diagnosis and treatment of diabetes, as highlighted for sub-Saharan Africa,^25^ is likely to be a major contributor to these early deaths. The diabetes burden of these countries calls for global action in alignment with the UN Sustainable Development Goal 3, which emphasises the provision of universal access to care and affordable essential medicines, and with WHO's Global Target 9 for confronting NCDs, which focuses on the availability of essential medicines, including insulin.^1^, ^2^ The *Lancet Global Health* Commission on high-quality health systems emphasised the importance of developing health systems in LMICs to permit broad access to quality health services.^26^ As highlighted by the *Lancet* Diabetes Commission, access to insulin, patient education, and tools for monitoring blood glucose concentration are important to prevent premature deaths and emergencies in young patients with type 1 diabetes.^27^ So far, there has been no means of periodic monitoring of this mortality goal globally. The use of the age-standardised death rate from diabetes at ages younger than 25 years can help to track achievements in basic diabetes health care**.** Additionally, the recently described increased risk of young people with type 2 diabetes^19^ could be similarly tracked.

This study has limitations. First, the vital registration data are generally of low quality or absent in many countries, especially in sub-Saharan Africa. However, the GBD, over decades, has developed a comprehensive methodology to address this problem, including yearly searches with in-country collaborators for available data; detailed cleaning, correction, and smoothing routines; and modelling approaches to maximise the use of what data are available. Second, few well established risk factors for type 1 diabetes exist, limiting the ability to extrapolate findings from data-rich countries. Third, our diabetes mortality indicator could have varied, in part, due to differences in the underlying prevalence of diabetes. However, within the age range analysed, prevalence was poorly associated with age-standardised diabetes death rates. Although lack of adjustment for diabetes prevalence could be an issue for a few countries with very high prevalence of type 2 diabetes, to keep the indicator simple we did not make this adjustment. In the future, as the prevalence of type 2 diabetes in the young increases, this adjustment might become indicated.

Within these limitations and recognising that the rates modelled for specific countries are approximations of their true rates, we describe a comprehensive and global overview of the level and trends in age-standardised diabetes mortality at early ages. The metric allows the monitoring of potentially avoidable diabetes deaths—those caused by acute complications and, to a lesser extent, chronic complications resulting from extremely poor glycaemic control—around the world. If meaningful progress on decreasing avoidable diabetes deaths is to be made on a world scale, it must be focused on low to high-middle SDI countries where 97·5% of the deaths due to diabetes in people younger than 25 years occur. Prevention of diabetes mortality can be improved with the prompt diagnosis and treatment of type 1 diabetes and with the provision of basic care and education to both people with all types of diabetes and their families.^24^ It is time for insulin, now 100 years after its discovery, to become available to all in need.

Globally, progress has been made in decreasing diabetes mortality at ages younger than 25 years, although important variability across countries remains. The decreases are less pronounced in low and low-middle SDI countries. Additionally, the large variability of this metric within each SDI quintile and its strong inverse correlation with the GBD's UHC index indicate that factors related to the organisation and quality of health care are important determinants of these outcomes. Diabetes mortality at ages younger than 25 years can serve as a readily available indicator for the surveillance of basic diabetes care and access to insulin around the world.

## Data sharing

To download the data used in these analyses, please visit the Global Health Data Exchange at http://ghdx.healthdata.org/gbd-2019/data-input-sources.

## Declaration of interests

J L Baker reports non-financial support as an unpaid speaker at Novo Nordisk symposiums (outside the submitted work). S Bhaskar reports institutional support from NSW Health Pathology (Australia), grants or contracts from the New South Wales (NSW) Ministry of Health NSW Brain Clot Bank (2019–22) in Australia, and paid or unpaid leadership or fiduciary roles in board, society, committee, or advocacy groups with the Rotary Club of Sydney (NSW, Australia) as a board director and chair of the Youth Committee and with the International Rotary Fellowship of Healthcare Professionals as board director (all outside the submitted work). I Y Elgendy acknowledges grants from Caladrius Biosciences, outside the submitted work. D Flood reports grants or contracts from US National Institutes of Health (NIH) funding comparative health systems research on diabetes indicators (grant P30-DK09292); unpaid leadership or fiduciary roles in board, society, committee, or advocacy groups with Maya Health Alliance as lead diabetes physician for this non-governmental clinical organisation in Guatemala, conducting unpaid advocacy on behalf of people with diabetes; and stock or stock options as co-founder and 1% co-owner of GlucoSalud, a diabetes social business in Guatemala (all outside the submitted work). N Ghith reports support for the present manuscript via a grant from the Novo Nordisk Foundation (NNF16OC0021856). N E Ismail reports an unpaid leadership or fiduciary role in board, society, committee, or advocacy group, with the Malaysian Academy of Pharmacy as a council member (outside the submitted work). K Krishan reports non-financial support from the UGC Centre of Advanced Study, CAS II, Department of Anthropology, Panjab University, Chandigarh, India (outside the submitted work). U O Mueller reports a grant from the US National Institute of Aging to the University of Washington, granted as a subaward to the Center for Population and Health, and paid or unpaid leadership or fiduciary roles in board, society, committee, or advocacy group with the Center for Population and Health as the chairman (all outside the submitted work). M P Schlaich reports grants or contracts from Boehringer Ingelheim in support for an investigator-initiated study with SGLT-2 inhibitors in patients with diabetes (outside the submitted work). J A Singh reports consulting fees from Crealta/Horizon, Medisys, Fidia, PK Med, Two Labs, Adept Field Solutions, Clinical Care Options, Clearview Healthcare Partners, Putnam Associates, FocusForward, Navigant Consulting, Spherix, MedIQ, Jupiter Life Science, UBM, Trio Health, Medscape, WebMD, Practice Point communications, the NIH, and the American College of Rheumatology; payment or honoraria for lectures, presentations, speakers' bureaus, manuscript writing, or educational events from Simply Speaking; support for attending meetings or travel from OMERACT, an international organisation that develops measures for clinical trials and receives arm's length funding from 12 pharmaceutical companies, when travelling to OMERACT meetings; participation on a data safety monitoring board or an advisory board as a member of the US Food and Drug Administration Arthritis Advisory Committee; paid or unpaid leadership or fiduciary roles in board, society, committee, or advocacy groups with OMERACT as a member of the steering committee, with the Veterans Affairs Rheumatology Field Advisory Committee as a chair member, and with the UAB Cochrane Musculoskeletal Group Satellite Center on Network Meta-analysis as director and editor; stock or stock options in TPT Global Tech, Vaxart Pharmaceuticals, Atyu Biopharma, and Charlotte's Web Holdings and previously owned stock options in Amarin, Viking, and Moderna pharmaceuticals (all outside the submitted work). M Tonelli reports payment or honoraria for lectures, presentations, speakers' bureaus, manuscript writing, or educational events from AstraZeneca (outside the submitted work). R Uddin reports grants from Deakin University (Geelong, VIC, Australia) through an Alfred Deakin Postdoctoral Research Fellowship and support for attending meetings or travel from the Deakin University Institute for Physical Activity and Nutrition (all outside the submitted work). S Zadey reports unpaid leadership or fiduciary roles in other board, society, committee, or advocacy groups with the Association for Socially Applicable Research as the co-founding director.
