# Supplementary material for: Diabetes mortality and trends before 25 years of age: an analysis of the Global Burden of Disease Study 2019
Source: Lancet Diabetes Endocrinol. 2022 Mar;10(3):177–92. doi: 10.1016/S2213-8587(21)00349-1 (PMC8860753; doi:10.1016/S2213-8587(21)00349-1)

THE LANCET
Diabetes & Endocrinology

Supplementary appendix

This appendix formed part of the original submission and has been peer reviewed. We post it as supplied by the authors.

Supplement to: GBD 2019 Diabetes Mortality Collaborators*. Diabetes mortality and trends before 25 years of age: an analysis of the Global Burden of Disease Study 2019. *Lancet Diabetes Endocrinol* 2022; published online Feb 7. https://doi.org/10.1016/S2213-8587(21)00349-1.

Appendix to "Diabetes mortality under age 25: a readily accessible metric to monitor levels and trends in the provision of basic health care for diabetes"

Portions of this Appendix have been reproduced or adapted from Vos et al.,[1] Wang et al.,[2] and Lozano et al.[3]

Table of Contents

1. Section 1: Overview of GBD methodology 5
2. Section 2: All-cause mortality 6
3. Section 3: GBD 2019 Causes of Death database 7
3.1. Steps in Data Input 8
Step 1: Standardise input data 8
Step 2. Map to GBD cause list 9
Step 3. Split age-sex groups 9
Step 4. Correct for miscoding of Alzheimer's and other dementias, Parkinson's disease, and atrial fibrillation and flutter 10
Step 5. Redistribute 10
Step 6. Correct HIV/AIDS misclassification 10
Step 7. Scale strata to province 10
Step 8. Correct post-redistribution problems 10
Step 9. Drop VR country years or mark as non-representative 10
Step 10: Aggregate causes 11
Step 11: Remove shocks and HIV/AIDS maternal adjustments 11
Step 12. Apply noise reduction algorithms 11
Step 13. Identify outliers in the cause of death database 11
3.2. Data star rating for the quality of VR 12
4. Section 4. Causes of death modelling methods 15
4.1. CODEm 15
4.2. Causes modelled outside of CODEm 16
4.3. CoD Correct 16
4.4. GBD world population age standard 16
4.5. Statistical analyses 16
5. Section 5: Specific CoD modeling descriptions 16

5.1. Diabetes Mellitus ... 17
5.1.1. Overall Diabetes Mellitus ... 17
5.1.1.1. Input Data and Methodological Summary for diabetes mellitus ... 18
5.1.1.1.1. Input data ... 18
5.1.1.1.2. Modelling strategy ... 18
5.1.1.1.3. Covariate selection ... 18
5.1.1.1.4. Covariate Influences ... 20
5.2. Diabetes mellitus Type 1 and Type 2 ... 23
5.2.1. Input Data and Methodological Summary for Type 1 and Type 2 diabetes mellitus ... 25
5.2.1.1. Input Data ... 25
5.2.1.2. Modelling strategy ... 25
5.2.1.3. Covariate selection ... 26
5.2.1.4. Covariate Influences: ... 26
5.3. Chronic Kidney Disease ... 29
5.3.1. Input data ... 29
5.3.2. Modelling strategy ... 29
5.3.3. Covariate Influences ... 30
5.4. Chronic Kidney Disease subtypes, including those due to type 1 and type 2 diabetes ... 31
5.4.1. Input data ... 31
5.4.2. Modelling strategy ... 31
6. Socio-demographic Index ... 32
7. Universal Health Coverage (UHC) Effective Care Index ... 32
8. Specific analysis for the creation of the diabetes mortality underage 25 indicator ... 33
8.1. Aggregating deaths due to diabetes ... 33
8.2. Age-standardisation ... 33
9. GATHER Checklist ... 36
References ... 38
Additional Results in Tables and Figures ... 39
Supplementary Table 1 – List of countries by SDI quintile ... 40
Supplementary Table 2 - Age-standardised deaths rate (95% Uncertainty interval) due to diabetes under age 25, 1990 and 2019 , percentage change in rates 1990-2019, percent of total deaths, number of deaths, and population. ... 45
Supplementary Figure 1 – Map showing SDI quintile for each country. ... 53

Supplementary Figure 2: Diabetes (type 1 and type 2 combined) mortality rates under age 25 by Socio-demographic Index (SDI) groups A) Trends in age-standardised mortality rate from 1990 to 2019 B) Age-specific mortality in 2019. 54

Supplementary Figure 3: Age-standardised mortality rate (per 100,000) due to type 1 diabetes under age 25 in 2019. 56

Appendix Figures

Appendix Figure 1. Analytical flowchart for the estimation of all-cause mortality by age and sex, and HIV/AIDS incidence, prevalence, and mortality for GBD 2019

Appendix Figure 2. Classification of national time series of vital registration and verbal autopsy data 2010−2018

Appendix Tables

Appendix Table 1 – GBD standard world population under age 25

Appendix Table 2. Calculation of mortality from diabetes under age 25 in 2019 using publicly available GBD data as an example of calculations performed to generate estimates.

List of Abbrevations

Abbreviation	Meaning
BTL	basic tabulation list
CKD	chronic kidney disease
CoD	causes of death
CODEm	Cause of Death Ensemble modelling
DisMod-MR	disease model-Bayesian meta-regression
GATHER	Guidelines for Accurate and Transparent Health Estimates Reporting
GBD	Global Burden of Diseases, Injuries, and Risk Factors Study
GHDx	Global Health Data Exchange
ICD-	International Classification of Diseases
IHME	Institute for Health Metrics and Evaluation
LDI	lag-distributed income per capita
LMER	linear mixed effects regression
SDI	Socio-demographic Index
ST-GPR	spatiotemporal Gaussian process regression
UI	uncertainty interval
VA	verbal autopsy
VR	vital registration
WHO	World Health Organization

This Appendix provides methodological details and supplemental figures and tables. Briefly, it summarizes details presented principally in the Methods Appendix to "Global Burden of 369 diseases, injuries, and impairments, 1990-2019: a systematic analysis for the Global Burden of Disease Study 2019" (Named hereafter Capstone Appendix),[1] but also in the methods appendices of additional GBD 2019 publications.[2,3] Our aim is to give a comprehensive description of the analytical steps taken, with tables, figures and specific details to make transparent our estimation processes.

1. Section 1: Overview of GBD methodology

The GBD 2019 applies a standard methodological approach to generate estimates for mortality and causes of death for diseases for 204 countries and territories.

We grouped countries and territories into 21 regions and these into seven super-regions: 1) Central Europe, Eastern Europe, and Central Asia; 2) High Income; 3) Latin America and the Caribbean; 4) North Africa and the Middle East; 5) South Asia; 6) Southeast Asia, East Asia and Oceania; and 7) sub-Saharan Africa.

GBD organizes causes of death based on the GBD cause list, which is hierarchical, comprising four levels:

- At level 1, there are three cause groups: Group 1, communicable, maternal, neonatal, and nutritional diseases; Group 2, non-communicable diseases, including diabetes and chronic kidney disease; and Group 3, injuries.
- At level 2, these level 1 groups are subdivided into 22 cause groups, with diabetes and chronic kidney disease (CKD) being grouped together.
- At level 3, diabetes mellitus and chronic kidney diseases are disaggregated.
- At level 4, type 1 diabetes, type 2 diabetes, chronic kidney disease due to type 1 diabetes and chronic kidney disease due to type 2 diabetes are disaggregated to contains the finest detail for these causes captured in GBD 2019.

GBD publications comply with the Guidelines for Accurate and Transparent Health Estimates Reporting (GATHER) recommendations.[4] The steps in our analytical procedures and detailed data sources can be found in the Capstone Appendix (with Table 1 for the GATHER checklist). To check the GATHER recommendations visit the GATHER website under GATHER Statement.

GBD 2019 synthesizes a large and growing number of data input sources, including surveys, censuses, vital statistics, and other health-related data sources which are used to estimate mortality. The input sources are accessible through an interactive citation tool available in the Global Health Data Exchange (GHDx; http://ghdx.healthdata.org/). This tool allows users to view and access GHDx records for input sources and export a comma-separated value (CSV) file that includes metadata, citations, and information on where data were used in GBD. Citations for specific GBD components, causes and risks, and locations can also be found with this tool. As required by GATHER, additional metadata for input sources are available through the citation tool as well.

The GBD permits visualization of its results online. All GBD 2019 online data visualizations are available at https://vizhub.healthdata.org/gbd-compare/, which provides results for all GBD health metrics. Core summary GBD 2019 results, including for deaths, can be downloaded in tabular form with the GBD´s data download tool, available at http://ghdx.healthdata.org/gbd-results-tool. Data above a certain size cannot be viewed online but can be downloaded. Depending on the size of the download, users may need to enter an e-mail address; a download location will be sent to them when the files are prepared.

2. Section 2: All-cause mortality

The calculation of all-cause mortality estimates for all GBD age groups, by sex, for all locations and years is described in detail in the Methods Appendix to the 2019 GBD publication "Global, regional, and national age-sex-specific fertility, mortality, and population estimates, 1950–2019: a comprehensive demographic analysis for the Global Burden of Disease Study 2019".[2]

We calculated all-cause mortality based on the integration of data from a diverse set of sources. To estimate child mortality, we used data from vital registration (VR) systems, sample registration systems and disease surveillance point systems, household surveys (complete and summary birth histories), censuses (summary, and on rare occasions, complete birth histories), and demographic surveillance sites. To estimate adult mortality, we used, among others, VR systems and surveys and censuses from which we extracted household death recall data.[2]

Calculations were complicated by the fact that not all countries and territories have complete vital registration (VR) systems recording the event of death nor periodic censuses. Thus, our processes adjusted for the completeness (quality) of available VR data. [2]

We estimated incompleteness in VR sources for deaths under age 5 in mixed effects non-linear models, as described in section 2.2.6 of the Methods Appendix to "Global, regional, and national under-5 mortality, adult mortality, age-specific mortality, and life expectancy, 1950-2017: a systematic analysis for the Global Burden of Disease Study 2017".[5] In this process, for each country, we initially relied on expert opinion to choose a source, or combination of sources, which were believed to be the least biased. If a country had a VR system which we deemed to be complete, this was the reference source. If a country did not have a complete VR system, but had estimates from complete birth histories, these were used as the reference source. If a country had neither of these types of data, or complete birth histories estimates were deemed unreliable, we assigned the surveys conducted after 1950 (in combination) as the reference. Incomplete VR data were not included. Additionally, in many countries we chose alternate surveys as the reference. For accurate estimation, it was important to have local knowledge on specific data sources' accuracy. All-cause mortality experts drew from their familiarity with data quality to help us to choose the reference category.[2]

To determine incompleteness of sources at other ages, we next combined our findings of under-five VR data completeness with death distribution methods to estimate completeness for adults aged 15 to 59. Here, we used the three death distribution methods most common in demography: generalized growth balance, synthetic extinct generation, and a combined approach, which estimate completeness by comparing the age distribution of the population between two censuses with the age distribution of deaths between those same censuses. We also applied two additional death distribution methods that utilize the GBD Bayesian Population Model.[2]

As shown in Appendix Figure 1, aside from estimating completeness, five major methodological tasks were executed in estimating all-cause mortality: estimating the probability of death between birth and age 5 years (5q0); estimating the probability of death between age 15 years

and 60 years (45q15); estimating a complete set of age-specific mortality rates; estimating HIV mortality; and producing final estimates of age-specific mortality, including HIV mortality and fatal discontinuities. Estimates of overall mortality by age, sex, location, and year were the outputs of this process. These estimates were used for ages 15 and above, and a combination of these under-5 and adult estimates produced completeness estimates to be used for ages 5 to 9 and 10 to 14.[2]

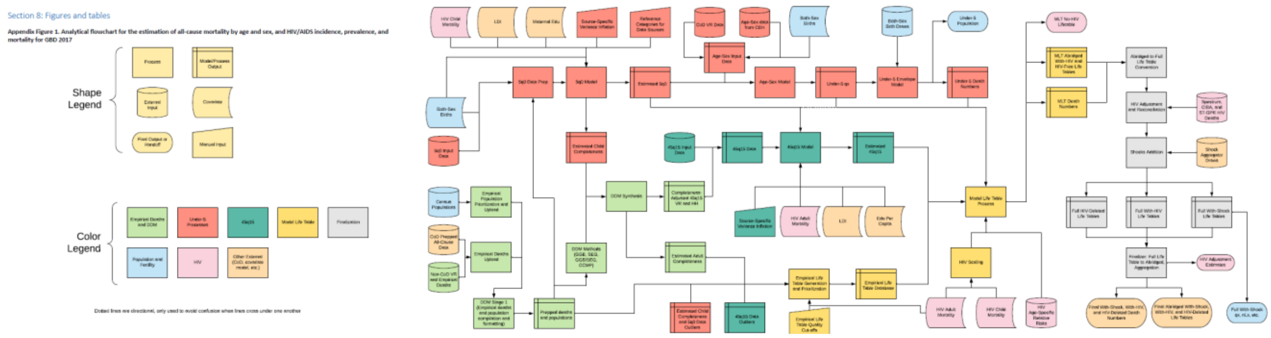

Appendix Figure 1. Analytical flowchart for the estimation of all-cause mortality by age and sex, and HIV/AIDS incidence, prevalence, and mortality for GBD 2019

3. Section 3: GBD 2019 Causes of Death database

Data sources for causes of death were obtained from vital registration systems, verbal autopsies, and other surveillance systems for 1990–2019.[6]

All available data on causes of death (CoD) data are standardized, based on International Classification of Diseases (ICD) 9 and 10 code mapping for diabetes and other GBD causes, and pooled into a single database used to generate cause-specific mortality estimates by age, sex, year, and geography. This process passes through several steps which are outlined below. Appendix Figures 1 and 2 of the Capstone Appendix show the high-level view of data inputs, analytical steps, and outputs of the causes of death (CoD) analysis frame.[1]

The CoD database contains seven types of data sources (Capstone Appendix Table 3), including vital registration (VR), verbal autopsy (VA), sibling history, and survey/census. In countries with complete VR systems, there is no need to use any other data source. Less than half the world's population has deaths captured in a VR system, therefore, for countries with incomplete VR systems, vital statistics for causes of death may be supplemented withother data types (Capstone Appendix Figure 3).[1] Data inputs used to generate the estimates are available at http://ghdx.healthdata.org/gbd-2019/data-input-sources.

A majority of the CoD data is VR data obtained from the World Health Organization (WHO) Mortality Database, a compilation of data submitted to the WHO by individual countries. VR is also obtained from country-specific mortality databases operated by official offices. Each cause is coded directly to the most detailed CoD when possible, whereas cause codes in data tabulated by International Classification of Disease (ICD-) are coded to aggregated cause groups.

Many countries use ICD Tabulation lists. The ICD-tabulation lists include the ICD-9 Basic Tabulation List (BTL), the ICD-10 Mortality Tabulation, the Russia Tabulation, and the India Medical Certification of Cause of Death. Two of the drawbacks in using tabulation lists are discrepancies in the accuracy of death counts and lack of detail due to aggregated cause groups.

There are instances where the sum of deaths in chapter subtotals are not equal to the sum of cause groups within the chapter. To account for any missing or duplicate deaths reported within the cause groupings, death counts are systematically adjusted by calculating the differences between subtotals and sub-causes within the cause groups. Any differences are assigned to a remainder cause group. To account for the lack of cause code detail, select cause groups are disaggregated to create a complete cause list.[1]

Sample registration systems are expanding in several countries and are key sources of data in Indonesia and India, as further detailed in the Capstone Appendix. In countries without VR systems, Verbal autopsy (VA) studies are a viable data source to inform CoD. Data are obtained by trained interviewers who use a standardised questionnaire to ask relatives about the signs, symptoms, and demographic characteristics of recently deceased family members. CoD is assigned based on the answers to the questionnaires. VA data are highly heterogeneous: studies use different instruments, different cause lists (from single causes to full ICD-cause lists), different methods for assigning CoD, different recall periods, and different age groups. Cultural differences may also affect the interpretation of specific questions. CoD validity must be considered when mapping to a GBD cause. VAs are likely accurate in assigning CoD to road injury or homicide but less accurate for causes requiring medical certification, such as diabetes or chronic kidney disease.[1]

3.1. Steps in Data Input

Processing of input data involves several steps, as follows:

Step 1: Standardise input data

The input data to the CoD database are received in various formats and must be standardised to run through central CoD machinery to then upload to the database. Raw data inputs come from data sources such as mortality databases, literature reviews, or reports. Usable data sources must have a clear sample size of the number of deaths in the population and exhaustive cause lists. The complexity of the data cleaning process varies drastically across data sources. For VR microdata with the location, age, sex, year, and ICD--coded cause of every death, very little effort is necessary to standardise it into a consistent structure. Other sources may require weeks of careful review to accurately extract scans of hardcover CoD reports into spreadsheets that can be transformed and standardised.[1]

At this point, data are assigned source identifiers so that they can be linked to the GHDx and cited appropriately. Any aggregate age and sex categories are flagged for age-sex splitting. The methods of cause-of-death assignment and data collection are reviewed to determine which source type to assign; for example, we distinguish sibling history data from surveys with a VA module. Only data at the most detailed level of the GBD location hierarchy are used. Documentation from the source is reviewed to determine if the population is representative of the location or only a subset of the population in that location. Data sources representing a subset of the population are flagged as non-representative; this flag is used by Cause of Death Ensemble modelling (CODEm) to increase the variance associated with such data points.[1]

Finally, diagnostics are reviewed at this stage to avoid sending cleaning errors downstream. We review cause-specific deaths for each demographic group to ensure the data are reasonable. For example, it is unlikely that deaths from neonatal causes occur in age groups over one year. All death totals are compared with the sum of cause-specific deaths to ensure the observed deaths are accounted for and sample size is complete.

CoD in tabulated VR data are condensed into aggregated groups, some of which can be mapped directly to GBD causes, while other aggregated cause groups are not informative and

cannot be mapped to them. To correct for this, aggregated causes were mapped and split onto multiple ICD-8, ICD-9, and ICD-10 detail causes, or targets, based on the ICD-groupings within the aggregated causes. ICD-8, ICD-9, and ICD-10 detail codes serve as targets because they are the highest-quality VR data and enable the calculation of proportions used to split the aggregated cause data into detailed causes. The proportions of deaths from nearby countries within the super-region were used to fill in data gaps as they were likely to have similar CoD trends.[1]

We determined the targets based on detail causes missing from the tabulated cause list. For any cause and demographic group for which we lacked ICD-detail, global proportions were used. State splitting and calculation of non-maternal deaths complete this step.[1]

Step 2. Map to GBD cause list

In GBD 2019, we used 439 maps to translate causes found in the input data to the GBD 2019 cause list. This included 31 maps for VR data, 314 for VA data sources, and 98 for other data types. The largest, and most universal, maps used were those for ICD-9 and ICD-10 VR data. Our mapping process enabled us to compare these various data sources across demographic groups.[1]

In GBD 2019, we developed additional maps to translate ICD-codes found in the input data that are non-underlying causes to appropriate target codes based on the levels of the GBD cause list.[6] These garbage codes were mapped to Levels 1-4 of the GBD cause list according to the following criteria:

1. Level 1 garbage codes include all codes for which a Level 1 GBD cause cannot be directly assigned. For example, the underlying causes of "sepsis" or "peritonitis", if not specified in the data, could be an injury, a non-communicable disease, or a type of communicable disease. In these cases, deaths will be redistributed across all three of the Level 1 causes. In addition, deaths coded to impossible or ill-defined causes of death (including "senility" and "unspecified causes") fall into this category, as they will be redistributed onto all causes.
2. Level 2 garbage codes include all codes that can be assigned within the same Level 1 GBD cause, being redistributed onto Level 2 causes.
3. Level 3 garbage codes include all codes that can be assigned within the same Level 2 GBD cause, being redistributed onto Level 3 causes.
4. Level 4 garbage codes include all codes (e.g., "unspecified diabetes mellitus") that can be assigned within the same Level 3 GBD cause, being redistributed onto Level 4 causes.

Step 3. Split age-sex groups

Different sources, particularly VA studies, report deaths for a wide range of age groups with varying intervals. For the analysis of CoD, we mapped these different age intervals to the GBD standard set of age groups. The Capstone Appendix displays formulas used for this purpose. In some cases, deaths are reported for an aggregate age group for both sexes combined. The task in thiscase is more complicated, but the same principle can be applied. In this case we assumed that the relative risks of death by age and sex are constant.[1]

We next adjusted separately for estimated adult and child VR completeness. Location-year-age-sex- cause specific deaths and population were then aggregated across all location-years, to produce cause- specific mortality rates by age and sex. These were used to determine the risk of death at any age relative to any reference age group, as shown in the above equations.[1]

Occasionally, data sources include deaths by a cause for which medical consensus exists that death is impossible for the sex and age. For example, some number of deaths may be attributed

to cervical cancer in males, or to maternal causes in children younger than 10 years. We have constructed a conservative list of age-sex restrictions. When deaths violate these restrictions, we redistribute them proportionally onto all causes. All restrictions are included, in the Capstone Appendix, in Appendix Table 5, Restrictions on age and sex by cause for GBD 2019.[1]

Step 4. Correct for miscoding of Alzheimer's and other dementias, Parkinson's disease, and atrial fibrillation and flutter

This step, less relevant for diabetes and CKD calculations, is described in the Capstone Appendix.

Step 5. Redistribute

A crucial aspect of enhancing the comparability of data for CoD is to deal with uninformative, so-called garbage codes. Garbage codes to which deaths were assigned should not be considered as the underlying CoD--for example: "heart failure", "ill-defined cancer site", "senility", "ill-defined external causes of injuries", and "septicaemia". The methods for redistributing these garbage-coded deaths were outlined in detail in Johnson SC. Because of the disparate nature of HIV/AIDS mortality across space and time, dynamic redistribution of HIV/AIDS-related garbage codes was applied.[6]

For each redistribution package, we defined the "universe" of data as all deaths coded to either the package's garbage codes or the package's redistribution targets for each country, year, age, and sex. We then ran a regression, the formula for which is given in the Capstone Appendix, separately for each target group and sex. In GBD 2019, we updated the regressions for stroke and diabetes. We dropped the proportion of garbage from the regression formula and ran regression on high-quality, low proportion garbage data (4/5 stars, < 50% GC). We also included all covariates included in the CODEm models for both stroke and diabetes.[6]

Step 6. Correct HIV/AIDS misclassification

This step, little relevant to diabetes and CKD calculations, is described in detail in the Capstone Appendix.[1]

Step 7. Scale strata to province

This step, related specifically to calculations related to China, is described in detail in the Capstone Appendix.[1]

Step 8. Correct post-redistribution problems

This step ensures that the detail of the cause list at this point in the data prep process is reasonable given the detail of the original data source and the methods by which the CoD was assigned. Two primary corrections are applied. First, any cause that is purely an artifact of the redistribution machinery targeting too detailed a cause is aggregated up to the parent cause. Second, a "bridge map" is applied over a certain set of sources to ensure that they do not contain causes that could not reliably be determined by the methods used.[1]

Step 9. Drop VR country years or mark as non-representative

Lozano and colleagues[7] describe the negative impact that low-completeness VR data could have on CoD modelling for GBD 2010. In particular, in settings where a data source does not capture all deaths in a population, the cause composition of deaths captured might be different from

those that are not. For GBD 2019, VR location-years with completeness less than 50% were dropped, while location-years with completeness between 50% and 69% were marked as non-representative. In addition, any country-year with a number of deaths registered to major garbage codes greater than 50% of the deaths registered was dropped.[1]

Step 10: Aggregate causes

The cause list is organised in a top-down hierarchical format containing four levels. Deaths are divided into three broad groupings (Level 1 causes): “communicable, maternal, neonatal, and nutritional diseases”; “non- communicable diseases”; and “injuries”. Within the Level 1 grouping of non-communicable diseases is the level 2 cause “Diabetes and kidney diseases” which aggregates the level 3 causes “Diabetes mellitus” and “Chronic kidney disease”. “Diabetes mellitus” aggregates the level 4 causes “Diabetes mellitus type 1” and “Diabetes mellitus type 2”. “Chronic kidney disease” aggregates five level 4 causes: “CKD due to diabetes type 1”, “CKD due to diabetes type 2”, “Hypertensive CKD”, “Glomerulonephritis CKD” and “Other CKD”. The mortality estimate for a parent cause in the hierarchy represents the sum of the mortality due to causes under that rubric. Included in the parent Level 3 cause estimate are deaths mapped directly to the parent and any Level 4 sub-causes.[1]

Step 11: Remove shocks and HIV/AIDS maternal adjustments

For GBD 2019, CODEm models use an HIV/AIDS- and shock-free envelope. To be comparable, cause fractions must also be HIV/AIDS- and shock-free. Cause fractions were uploaded to the CoD database as the number of deaths due to the cause over an adjusted sample in which the number of deaths due to “HIV/AIDS”, “conflict and terrorism”, “executions and police conflict”, and “exposure to forces of nature” were removed.[1]

Step 12. Apply noise reduction algorithms

To deal with problems of zero counts in VR, VA, or sibling histories for a given age group in a given year, we use a Bayesian noise-reduction algorithm. For this algorithm, we assume a normal prior and a normal data likelihood. We estimate the normal prior for a given country-series of data by running a Poisson regression to estimate the number of deaths due to each respective cause and sex with dummy variables for age and year. With two exceptions, these regressions are sex-, cause-, and country-specific, so borrowing strength over age and year is only within a given data type, country, cause, and sex. Formula and greater detail are offered in the Capstone Appendix. The first exception is that country-years with populations under 1 million are pooled with the region data to prevent over-dispersion and provide a stronger signal. The second is that handling of VA data diverge from the above description in two ways. First, all data for a given super-region are pooled together and a study dummy variable is added, allowing for different studies and surveillance sites to borrow strength from one another within a super-region. Second, unless the data are part of a time series (e.g., the Matlab Health and Demographic Surveillance System), the regression has no year component.[1]

Step 13. Identify outliers in the cause of death database

Death rates for different CoD generally have a stable age pattern. In large populations, these patterns will not change very rapidly over time. We can assume a relatively stable pattern in death rates for all causes except for some epidemic diseases and specific types of injuries. Rare causes in large populations and prevalent causes in small populations usually have stochastic patterns. To

correct forthese stochastic patterns, we implemented a noise-reduction process, explained in Step 12.[1]

In VR data, we infrequently find one or more data points for specific geography/age/sex/year combinations that lie very far from the stable pattern of death rates. In these situations, the model usually ignores the data point(s). If the model fails to ignore these data, dramatic jumps or drops can occur in the death rates. When no logical explanation exists for variation in the death rates to this degree, we regard the data point(s) as outlier(s). The selection of data points to regard as outliers occurs after data have been prepped for modelling, as well as during preliminary reviews of the models.[1]

In non-VR sources, data-collection methods and data quality can vary widely from source to source. Where data points in each age-sex-geography-year are very sparse, extreme data points can have a bad effect on regional estimation. In these situations, we investigate the study's methods and consider lower-quality data points as outliers.[1]

Identifying outliers in the CoD data occurs prior to finalisation of models for each cause. We do not automate the selection of outliers but investigate the source of the offending data as well as reviewing other data sources for the same cause, geography, and year. Ultimately, outliers are identified based on the judgement of the modeller and senior faculty. Outlier decisions are reversible and may be revisited.[1]

3.2. Data star rating for the quality of VR

GBD estimates are most accurate when computed with a full time series of complete VR with a low percentage of garbage codes. Even countries with the highest quality mortality registration systems continue to have major problems related to ill-defined causes of death. To deal with the inadequacies of vital registration, GBD developed a 5-star rating system to characterize quality of death reporting in terms of the fraction of deaths accurately certified. Countries improve in the star rating as they increase availability, completeness, and detail oftheir mortality data and reduce the percentage of deaths coded to ill-defined garbage codes or highly aggregated causes. Location and year specific information on completeness and data quality are listed in Capstone Appendix Figures 2 (Vital Registration and Verbal Autopsy data availability by country, 1980−2018) and Figure 4 (Percent of vital registration deaths assigned to major garbage codes for all ages and sexes by country) 1980-2018) provide.[1]

We assign "star" ratings to rate the quality of data for any given location year. The inputs that determinethis star rating are the percentage of total deaths determined to be major garbage (such as All, Ill-defined), and the level of completeness in the dataset. Causes such as "injuries" or "cancer" will also be included in major garbage percentage because this percentage includes useof highly aggregated causes. These three values were used to create a "percent well-certified" value between 0 and 1, determined as:

Percent well certified = Completeness x (1 –Percent major garbage)

The mapping of percent well certified to star rating is as followed:

- 5 stars if percent of data well certified equaled or exceeded 85%;
- 4 stars for 65% to less than 85%;
- 3 stars for 35% to less than 65%;
- 2 stars for 10% to less than 35%;

- 1 star for greater than 0% to less than 10%; and
- 0 stars for 0% (no verbal autopsy or vital registration data were available over the period from 1980 to 2019).

Once percent well-certified is calculated for each location-year of VR and each VA study-year, we then combine these into one measurement for each five-year time interval and the full time series 1980–2019. For each five-year time interval, we assign the star level corresponding to that of the year with highest rating within the interval. Then for 1980–2019, we take the average of the maximum percentages well-certified for the seven five-year time intervals. Any five-year time interval in which no data were available were given a percent well-certified value of zero.

The number of countries at each star level over the over the full time series for all countries and countries included specific in this analysis (population greater than 1 million):

Number of countries at each star level.

Star level	All Countries	Countries with population > 1 million
5 stars	30	27
4 stars	43	33
3 stars	30	21
2 stars	29	21
1 star	43	38
0 stars	29	19

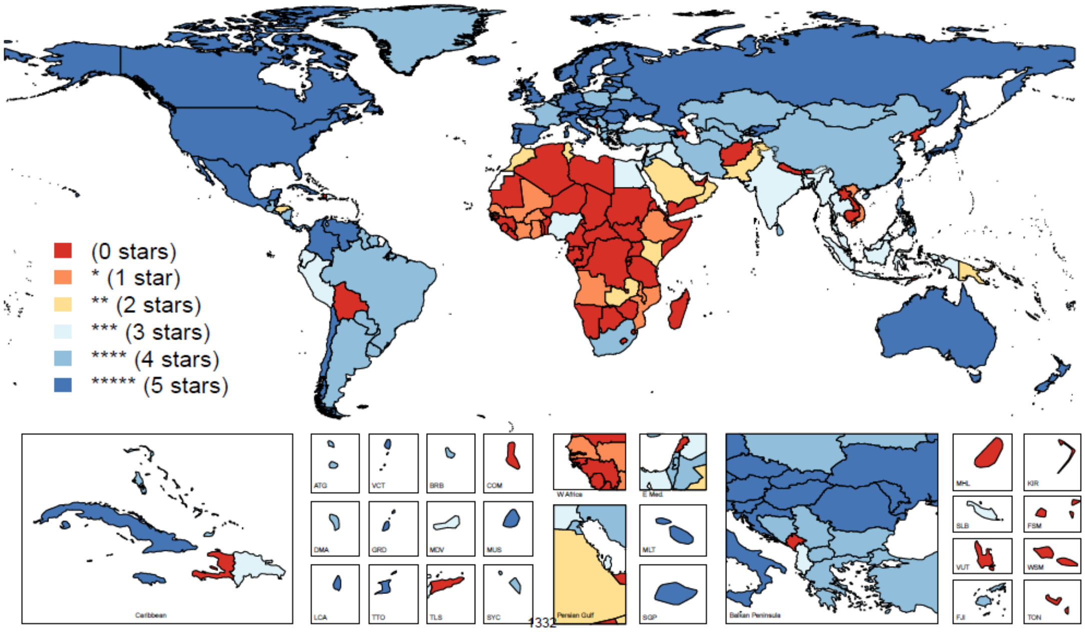

Appendix Figure 2. Classification of national vital registration and verbal autopsy data, showing the average star rating for locations for the period 2010-2018.

The GBD2019 Diseases and Injuries Capstone Appendix Figures 3 and 4 (pp 1441-1442) provide details of vital registration type and completeness, and percent of recorded deaths whose cause was identified as a major (level 1 or 2) garbage code for each of the countries analyzed.[1]

4. Section 4. Causes of death modelling methods

4.1. CODEm

Cause of death ensemble modelling (CODEm) is the framework used to model most cause-specific death rates in the GBD.[8] It relies on four key components:

First, all available data are identified and gathered to be used in the modelling process. Although the data may vary in quality, they all contain some signal of the true epidemiological process. Second, a diverse set of plausible models are developed to capture well-documented associations in the estimates. Using a wide variety of individual models to create an ensemble predictive model has been shown to outperform techniques using only a single model both in CoD estimation[9] and in more general prediction applications.[10] Third, the out-of-sample predictive validity is assessed for all individual models, which are then ranked for use in the ensemble modelling stage. Finally, differently weighted combinations of individual models are evaluated to select the ensemble model with the highest out-of-sample predictive validity.

For some causes evidence exists that the relationship between covariates and death rates might differ between children and adults. Separate models are therefore run for different age ranges, when applicable. Specifically, in the case of these analyses, deaths under age 15 are assumed to be due to type 1 diabetes, and above that age, due to type 2 diabetes. Additionally, separate models are developed for countries with extensive, complete, and representative VR for every cause to ensure that uncertainty can better reflect the more complete data in these locations.

Because many factors may co-vary with any given CoD, a range of plausible statistical models are developed for each cause. In the CODEm framework, four families of statistical models are used: linear mixed effects regression (LMER) models of the natural log of the cause-specific death rate, LMER models of the logit of the cause fraction, spatiotemporal Gaussian process regression (ST-GPR) models of the natural logarithm of the cause-specific death rate, and ST-GPR models of the logit of the cause fraction. The component models are weighted based on their predictive validity rank to determine their contribution to the ensemble estimate. A set of ensemble models is then created by using the weights.

The performance of all models (individual and ensemble) is evaluated by means of out-of-sample predictive validity tests. Thirty percent of the data are randomly excluded from the initial model fits. Individual model fits are evaluated and ranked by using half of the excluded data (15% of the total), then used to construct the ensembles based on their performance. These ensembles are tested by using the predictive validity metrics on the remaining 15% of the data, and the ensemble with the best performance in out-of-sample trend and root mean square error is chosen as the final model. Greater details of this process, including development of the model pool, data variance estimation, the testing of the model pool on a 15% sample, and ensemble development and testing are given in the Capstone Appendix.

Once a weighting scheme has been chosen, 1000 draws are created for the final ensemble, with the number of draws contributed by each model proportional to its weight. The mean of the draws is then used as the final estimate for the CODEm process, and a 95% uncertainty interval (UI) is created from the 0·025 and 0·975 quantiles of the draws. The validity of the UI can be checked via its coverage of the out-of-sample data; ideally, the 95% UI would capture 95% of these data. Higher coverage suggests that the UIs are too large, and lower coverage suggests overfitting.

4.2. Causes modelled outside of CODEm

CODEm is used to model both types of diabetes as well as CKD. However, the distribution of CKD deaths due to diabetes into the separate categories type 1 and type 2 diabetes is performed with DisMod-MR 2.1, which permits adjustment based on the prevalence of each type. Until GBD 2010, non-fatal estimates such as prevalence were based on a single data source on prevalence, incidence, remission, or a mortality risk selected by the researcher as most relevant to a particular location and time. Beginning with GBD 2010 a more ambitious goal was set: to evaluate all available information on a disease that passes a minimum quality standard. That required a different analytical tool that would be able to pool disparate information presented in varying age groupings and from data sources by using different methods. The DisMod-MR tool evaluates and pools all available data, adjusting data for systematic bias associated with methods that varied from the reference, and produces estimates with UIs by world regions.

Flow of data and settings is organized in an analytical cascade across different levels. The sequence of estimation occurred at five levels: global, super-region, region, country, and, where applicable, subnational locations. The super-region priors were generated at the global level with mixed-effects, non-linear regression by using all available data; the super-region fit, in turn, informed the region fit and so on down the cascade. The DisMod-MR 2.1 "wrapper" gives analysts the choice to branch the cascade in terms of time and sex at different levels depending on data density. The default used in most models was to branch by sex after the global fit but to retain all years of data until the lowest level in the cascade. Greater detail on DisMod-MR 2.1 is available in the Capstone Appendix.

4.3. CoD Correct

The CoD models are cause-specific. As such, there is no guarantee that the sum of these models will equal the results of the all-cause mortality estimates or that model results of child causes add up to the parent model results. The CoDCorrect process is used to make the CoD and all-cause mortality estimates internally consistent. The CoDCorrect process starts by rescaling the Level 1 causes to match the all-cause mortality estimates. Level 2 causes are then rescaled to their corrected parent causes. This process continues until all levels of the hierarchy have been rescaled.

4.4. GBD world population age standard

Age-standardised populations in the GBD were calculated by using the GBD world population age standard. We used the non-weighted mean of 2019 age-specific proportional distributions from the GBD 2019 population estimates for all national locations with a population greater than 5 million people in 2019 to generate an updated standard population age structure.[2]

4.5. Statistical analyses

GBD analyses were conducted with Python version 3.6.2, Stata version 13, and R version 3.5.0.

5. Section 5: Specific CoD modeling descriptions

The following text, flow charts and tables, as presented in the Capstone Appendix, describe details of modelling for diabetes, overall and by type, and CKD, overall and that due to type 1 and type 2 diabetes.

5.1. Diabetes Mellitus

Diabetes mellitus mortality was estimated for overall diabetes mellitus, diabetes mellitus type 1, anddiabetes mellitus type 2 in GBD 2019.

The following ICD codes were mapped to diabetes[1]:

Disease	ICD-9	ICD-10
Diabetes mellitus	250.00-250.99, 775.1	E10-E10.11, E10.3-E11.1, E11.3-E12.1, E12.3-E13.11, E13.3-E14.1, E14.3-E14.9, P70.2
Diabetes mellitus type 1	250-250.0, 250.01, 250.03-250.1, 250.11, 250.13-250.2, 250.21, 250.23-250.3, 250.31, 250.33-250.39, 250.5, 250.51, 250.53-250.6, 250.61, 250.63-250.7, 250.71, 250.73-250.8, 250.81, 250.83-250.9, 250.91, 250.93-250.99, 775.1	E10-E10.11, E10.3-E10.9, P70.2
Diabetes mellitus type 2	250.00, 250.02, 250.10, 250.12, 250.20, 250.22, 250.30, 250.32, 250.50, 250.52, 250.60, 250.62, 250.70, 250.72, 250.80, 250.82, 250.90, 250.92	E11-E11.1, E11.3-E11.9

The GBD map the ICD codes to the GBD cause list. In its analysis, the GBD does not use the details of coding in modeling (e.g. .0x-.9x) of codes 250 and E10-14.

5.1.1. Overall Diabetes Mellitus

Flowchart

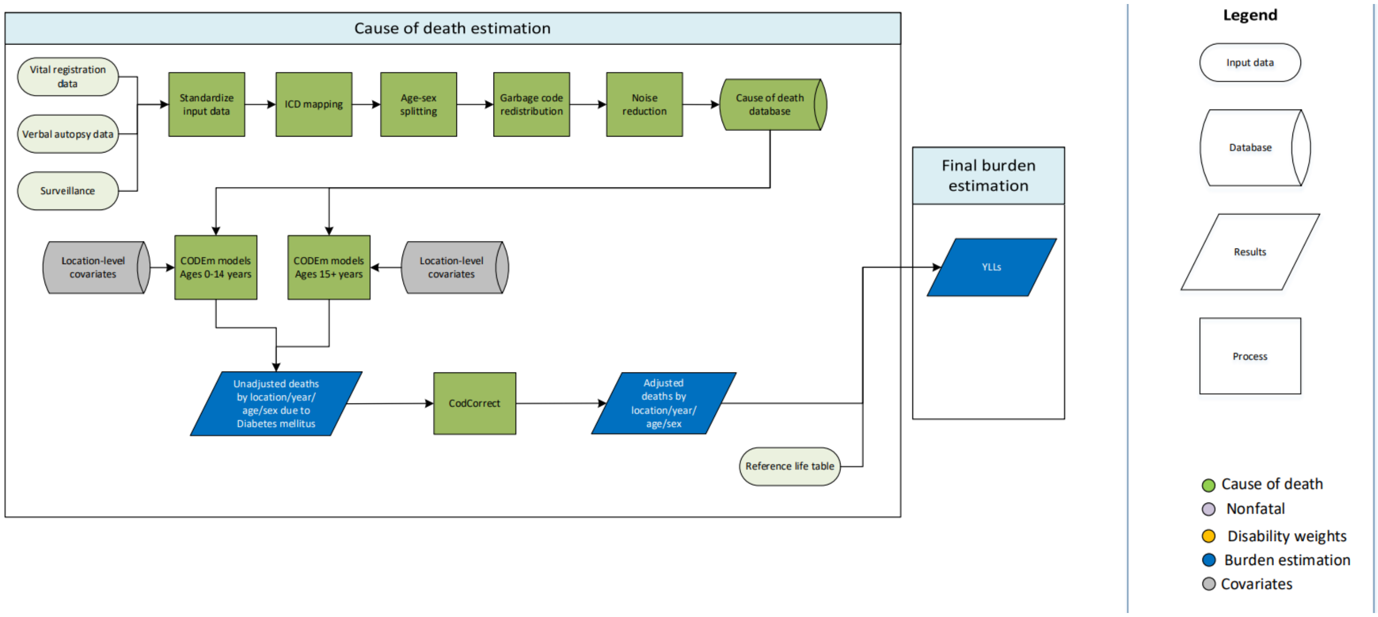

5.1.1.1. Input Data and Methodological Summary for diabetes mellitus

5.1.1.1.1. Input data

Overall diabetes mellitus mortality was estimated using deaths directly attributed to diabetes mellitus.We used verbal autopsy and vital registration data as inputs into the model.

Verbal autopsy data: We outliered data points from sources where there were zero deaths estimated inan age group as this was not realistic for deaths due to diabetes and we determined that these data sources were unreliable.

Vital registration data: We outliered all data from the India Medical Certification of Cause of Death report since the source of the data was unreliable according to expert opinion. We also outliered ICD9BTL data points that were inconsistent with the rest of the data series and created unlikely timetrends.

5.1.1.1.2. Modelling strategy

The Cause of Death Ensemble model (CODEm) was used for deaths due to diabetes mellitus estimation. In the overall diabetes mellitus model, we used two models to estimate overall diabetes deaths with different age restrictions. This is because deaths in younger age groups are almost exclusively due to type 1 diabetes, while deaths in older ages are primarily due to type 2 diabetes. This allowed us to select predictive covariates that are specific to the pathophysiology of diabetes type 1 and type 2. We set the younger age model from 0-14 years and the older age model from 15-95+ years. We determined the agethreshold based on evidence of the onset age of diabetes type 2 occurring at younger ages.

5.1.1.1.3. Covariate selection

The following table lists the covariates included in the model. This requires that the covariate selected for the model must have the directional relationship with diabetes mellitus deaths. In

GBD 2019, we made 2 updates. First, we changed 4 covariates to reflect the most current covariate available, proportion underweight to age-standardised underweight (weight-for-age) summary exposure variable, proportion stunting to age-standardised stunting (height-for-age) summary exposure variable, energy- adjusted grams of fruits to age- and sex-specific summary exposure variable for low fruit, and energy- adjusted grams of vegetables to age- and sex-specific summary exposure variable for low vegetables.

Second, we selected a direction on covariates that we did not set a direction in previous GBD. We determined the direction based on the strength of the evidence.

Model	Level	Covariate	Direction
0-14 years	1	Healthcare access and quality index	-
	3	Education years per capita	-
	2	Age-standardised fertility rate	+
	2	Latitude	+
	2	Age-standardised underweight (weight-for-age) summary exposure variable	-
	2	Percentage of births occurring in women >35 years old	+
	2	Percentage of births occurring in women >40 years old	+
	3	Socio-demographic Index	-
	2	Age-standardised stunting (height-for-age)summary exposure variable	-
	2	Mean birth weight	-
15 + model	1	Age-standardised mean fasting plasmaglucose (mmol/L)	+
	1	Age-standardised prevalence of diabetes	+
	3	Education years per capita	-
	3	Lag-distributed income per capita	+
	1	Mean BMI	+
	2	Mean cholesterol	+
	2	Mean systolic blood pressure	+
	1	Prevalence of obesity	+
	2	Age- and sex-specific summary exposurevariable for low fruit	-
	2	Energy-adjusted grams of sugar	+

Model	Level	Covariate	Direction

	2	Age- and sex-specific summary exposurevariable for low vegetables	-
	3	Healthcare access and quality index	-
	2	Age- and sex-specific summary exposurevariable for alcohol use	+

5.1.1.1.4. Covariate Influences

The following plots show the influence of each covariate on the four CODEm models (male global, male data rich, female global, and female data rich). A positive standardized beta (to the right) means that the covariate was associated with increased death. A negative standardized beta (to the left) means the covariate was associated with decreased death.

	Data rich	Global
Female<br>0-14	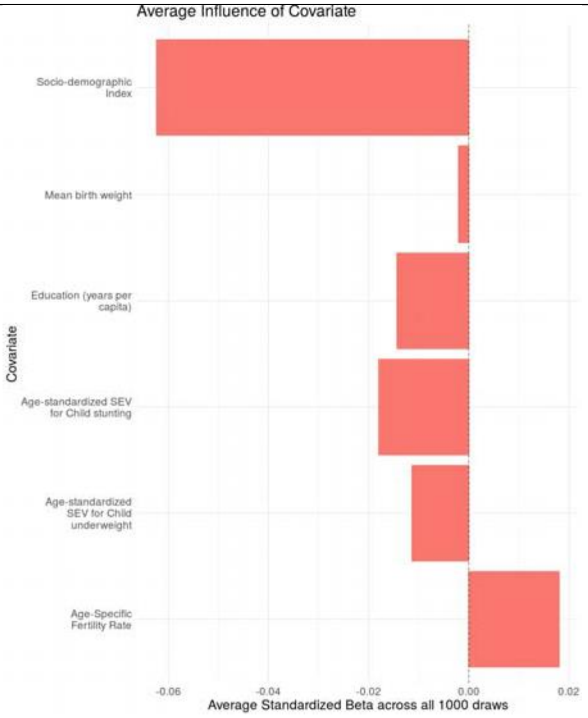	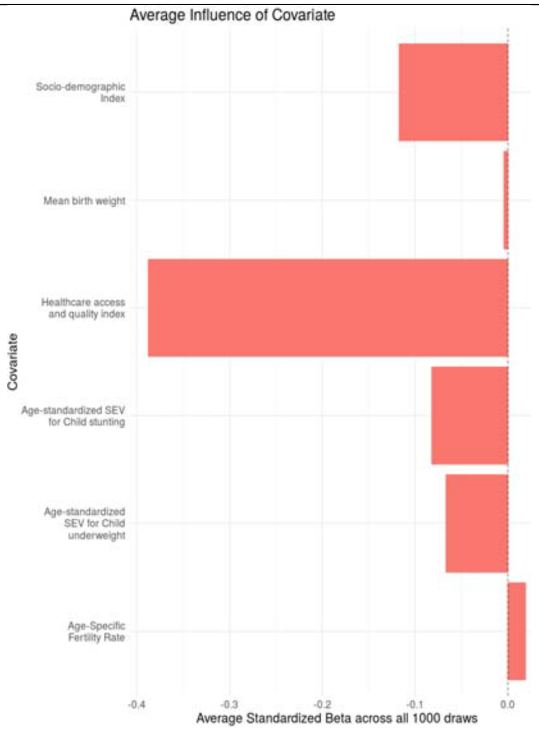

	Data rich	Global
Male 0-14 model	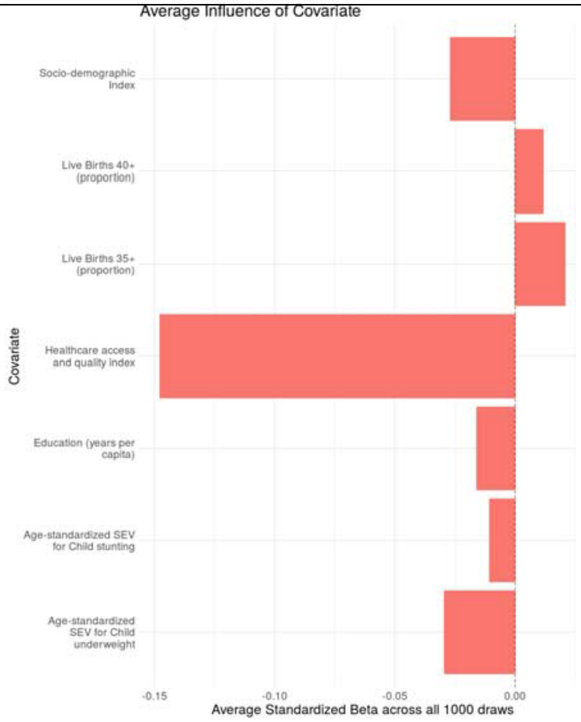	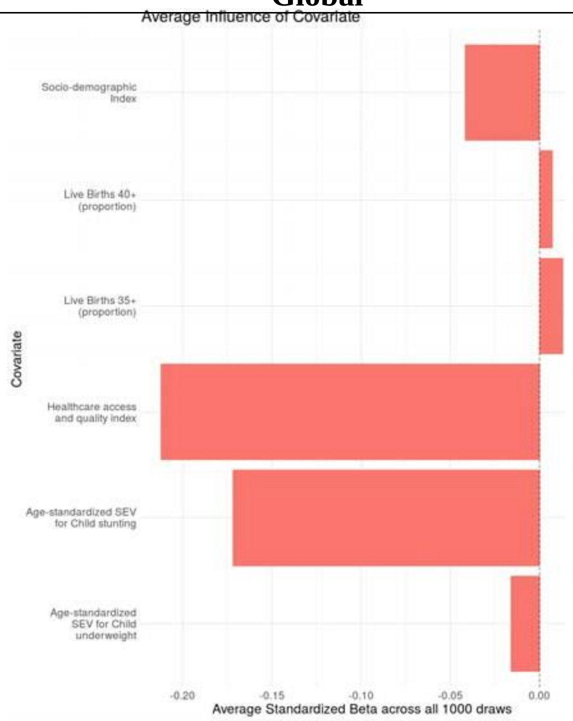
Female 15+ model	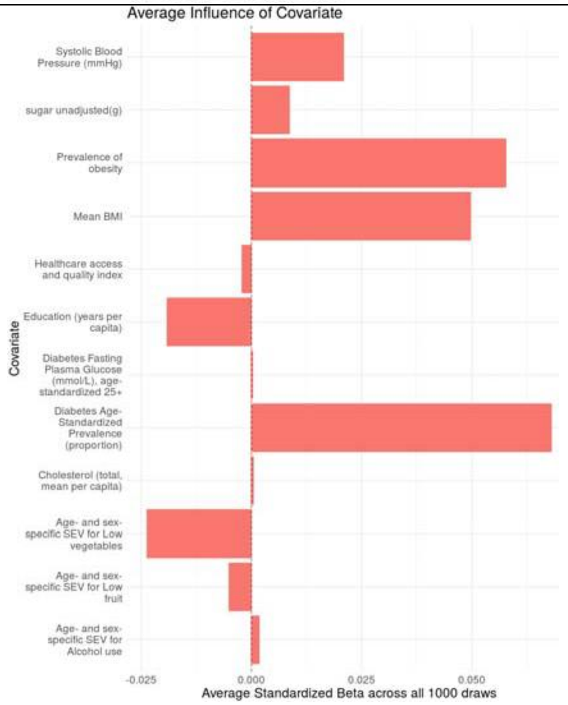	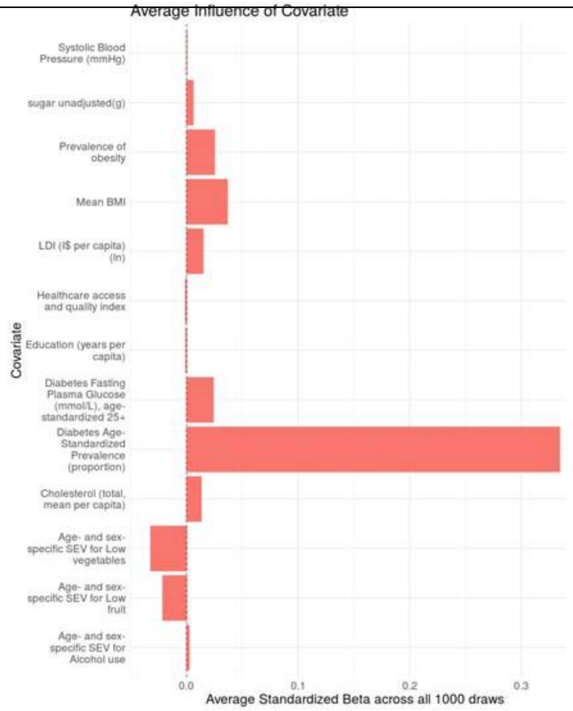

<table>
<tr><th></th><th>Data rich</th><th>Global</th></tr>
<tr><td>Male 15+ model</td><td>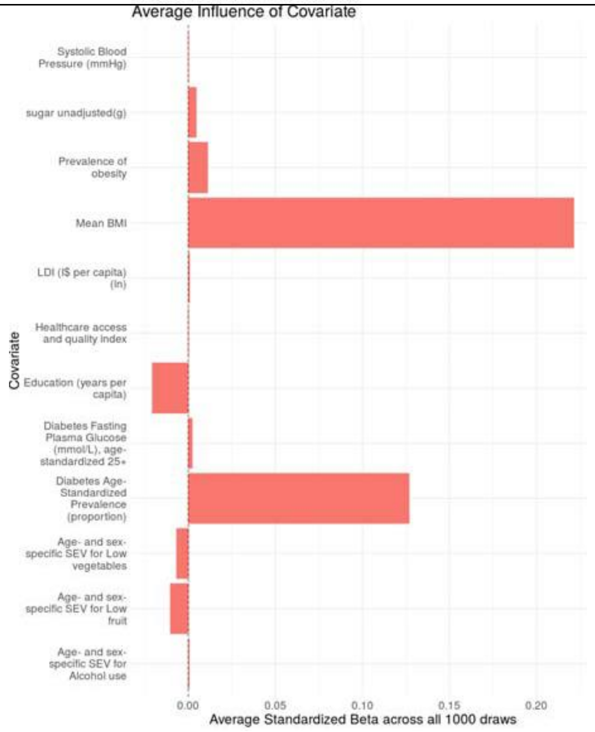
</td><td>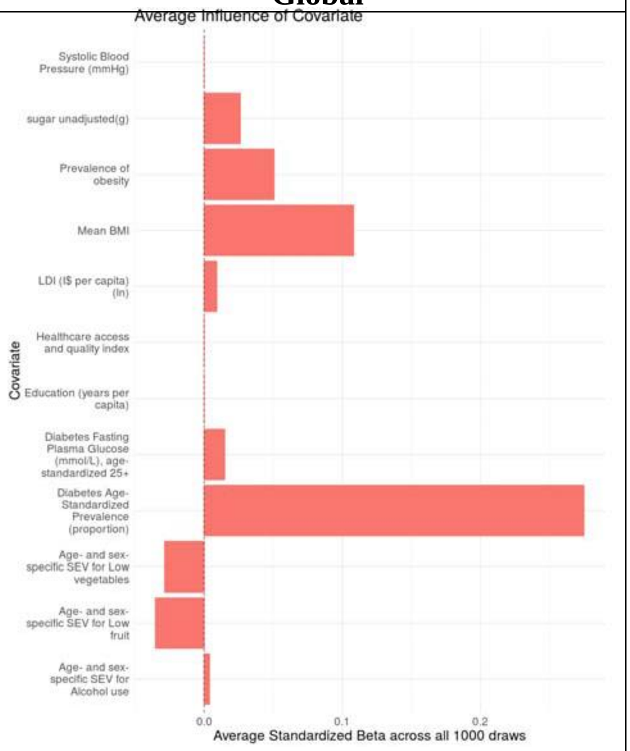
</td></tr>
</table>

5.2. Diabetes mellitus Type 1 and Type 2

Flowchart

Diabetes mellitus Type 1

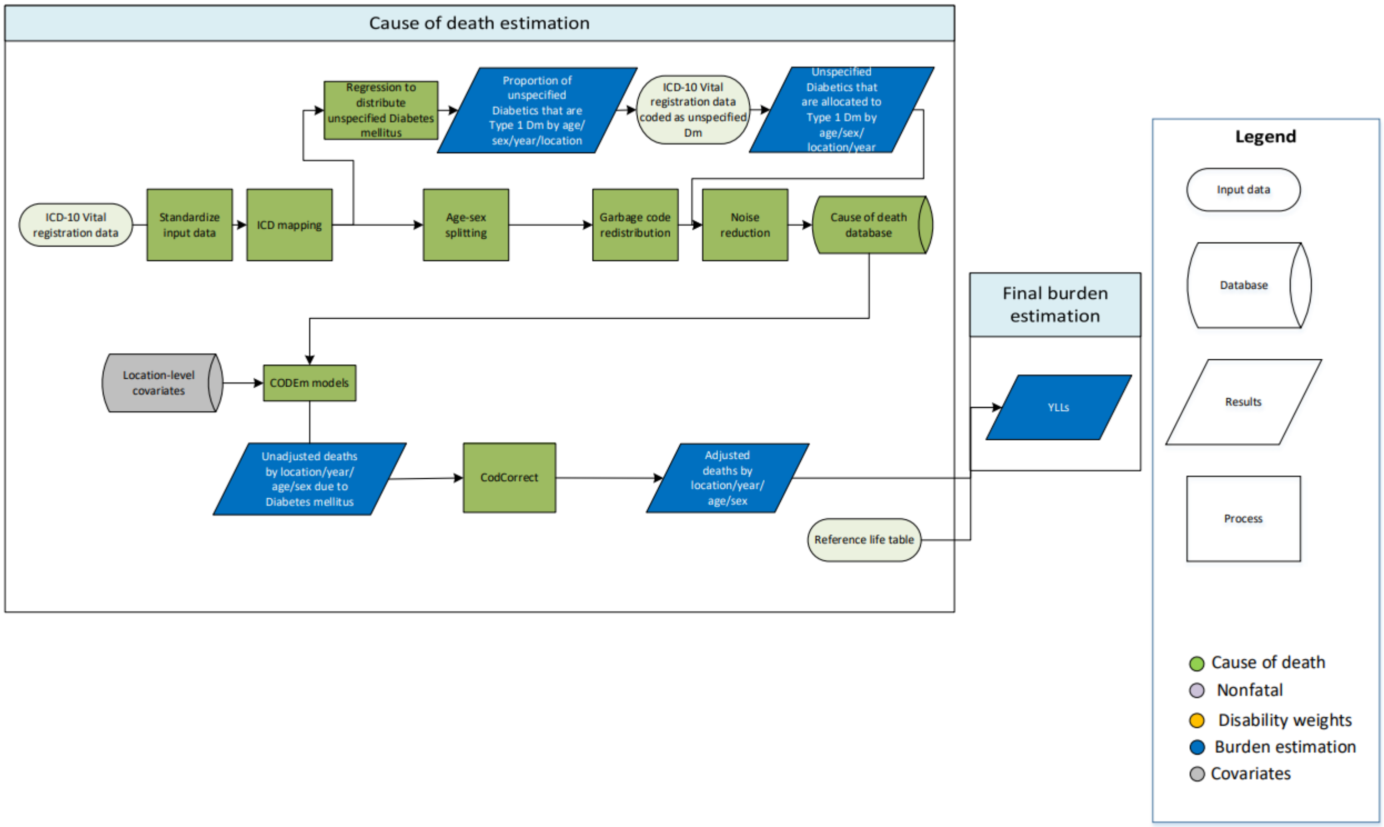

Diabetes mellitus Type 2

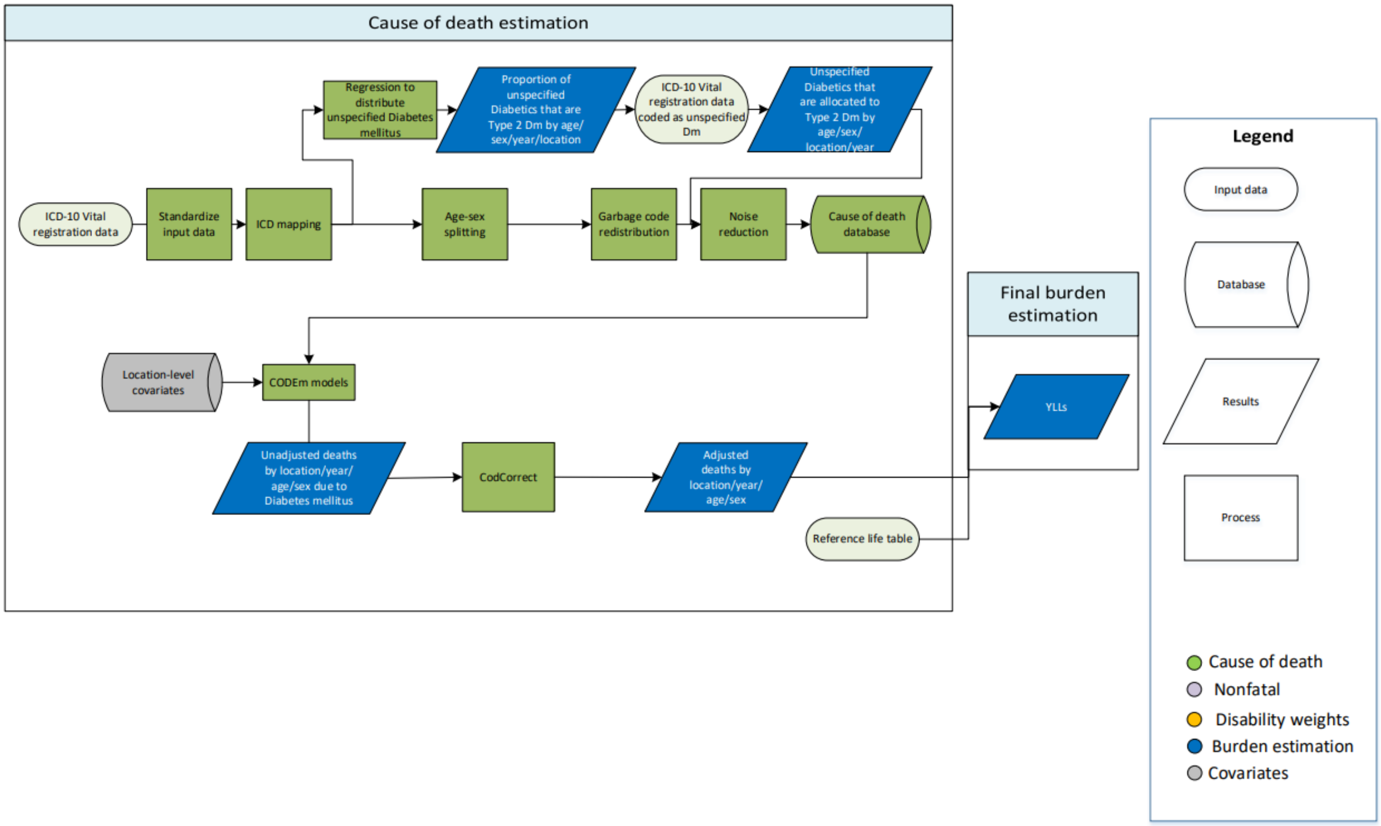

5.2.1. Input Data and Methodological Summary for Type 1 and Type 2 diabetes mellitus

5.2.1.1. Input Data

Type-specific diabetes mellitus mortality was estimated using deaths from vital registration sources in ICD-10 codes only. Diabetes type-specific information was not available in ICD-9 codes or deaths determined by verbal autopsy.

5.2.1.2. Modelling strategy

The Cause of Death Ensemble model (CODEm) was used for deaths due to diabetes mellitus estimation. Deaths in younger age groups are almost exclusively due to type 1 diabetes, while deaths in older agesare primarily due to type 2 diabetes. To account for this age pattern, we set the age range of the diabetes type 1 model to 0-95+ years and the age range of the diabetes type 2 model to 15-95+ years. We used the same covariates in the diabetes type 1 model and diabetes type 2 model as the 0-14 yearand 15-95+ year in the overall diabetes models, respectively.

There were two unique data manipulation steps that occurred to prepare the data as part of the modelling process.

1. We assumed that all deaths <15 years were due to type 1 regardless of the ICD-10 code assignedto the death. We imposed 100% attribution of diabetes mellitus deaths in <15 years to type 1 diabetes mellitus.
2. ICD-10 diabetes data were reported as type 1, type 2, or unspecified. We developed a regressionto estimate the fraction of unspecified diabetes mellitus that was type 1 and type 2. We only used data from 703 country-years to inform the regression. This is because these country-years had more than 50% of the deaths typed to type 1 or type 2 AND at least 70% of type-specific deaths in people >25 years were coded to type 2. Since there was a separate regression to estimate the proportion of type 1 diabetes mellitus and type 2 diabetes mellitus, we scaled the predicted proportions to one. These scaled proportions were then applied to number of deaths
3. coded to unspecified diabetes in each location, year, sex where ICD-10 data was reported.

<u>Regression equation</u>

Type 1:

$$logit\left(\frac{number\ type\ 1\ DM}{number\ total\ DM}\right) \sim logit\left(\frac{number\ unspecified\ DM}{number\ total\ DM}\right) + \beta_1 age\ group + \beta_2 age\text{-}st\ prev\ obesity * age\ group + age\text{-}st\ prev\ obesity$$

Type 2:

$$logit\left(\frac{number\ type\ 2\ DM}{number\ total\ DM}\right) \sim logit\left(\frac{number\ unspecified\ DM}{number\ total\ DM}\right) + \beta_1 age\ group + \beta_2 age\text{-}st\ prev\ obesity * age\ group + age\text{-}st\ prev\ obesity$$

5.2.1.3. Covariate selection

The following are the covariates included in the model. We selected the same covariates for the type 1 diabetes model as the 0-14 year diabetes model and the type 2 diabetes model as the 15-95+ year diabetes model.

Model	Level	Covariate	Direction
Type 1	1	Healthcare access and quality index	-
	3	Education years per capita	-
	2	Age-standardised fertility rate	+
	2	Latitude	+
	2	Age-standardised underweight (weight-for-age) summary exposure variable	-
	2	Percentage of births occurring in women >35 years old	+
	2	Percentage of births occurring in women >40 years old	+
	3	Socio-demographic Index	-
	2	Age-standardised stunting (height-for-age) summary exposure variable	-
	2	Mean birth weight	-
Type 2	1	Age-standardised mean fasting plasma glucose (mmol/L)	+
	1	Age-standardised prevalence of diabetes	+
	3	Education years per capita	-
	3	Lag-distributed income per capita	+
	1	Mean BMI	+
	2	Mean cholesterol	+
	2	Mean systolic blood pressure	+
	1	Prevalence of obesity	+
	2	Age- and sex-specific summary exposurevariable for low fruit	-
	2	Energy-adjusted grams of sugar	+
	2	Age- and sex-specific summary exposure variable for low vegetables	-
	3	Healthcare access and quality index	-
	2	Age- and sex-specific summary exposure variable for alcohol use	+

5.2.1.4. Covariate Influences:

The following plots show the influence of each covariate on the four CODEm models (male global, male data rich, female global, and female data rich). A positive standardized beta (to the right) means that the covariate was associated with increased death. A negative standardized beta (to the left) means the covariate was associated with decreased death.

Type 1 diabetes

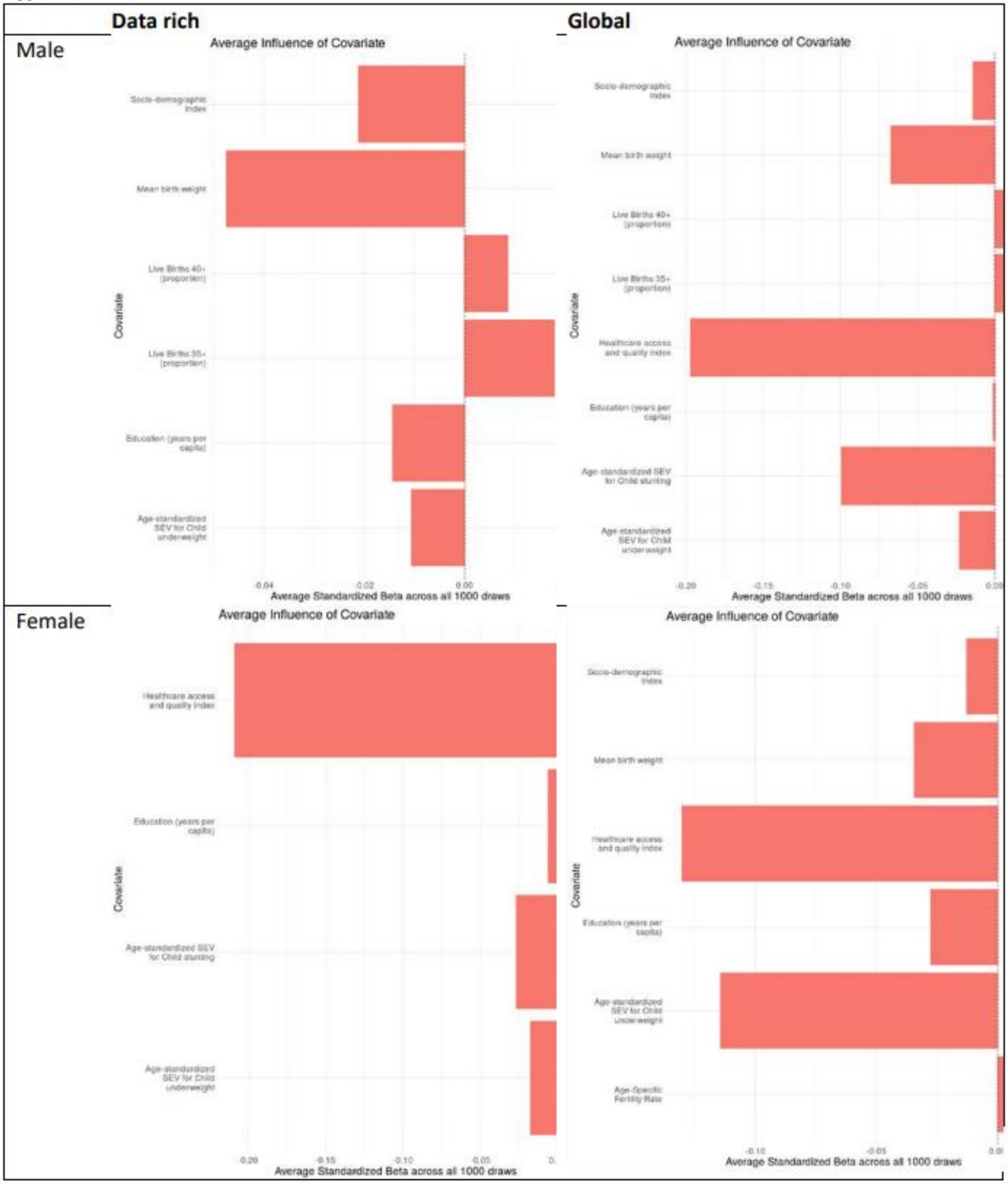

Type 2 diabetes

	Data rich	Global
Male	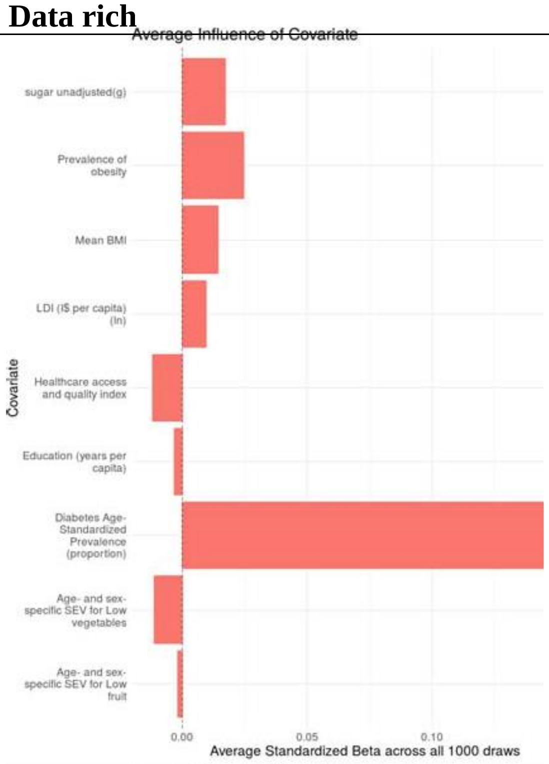	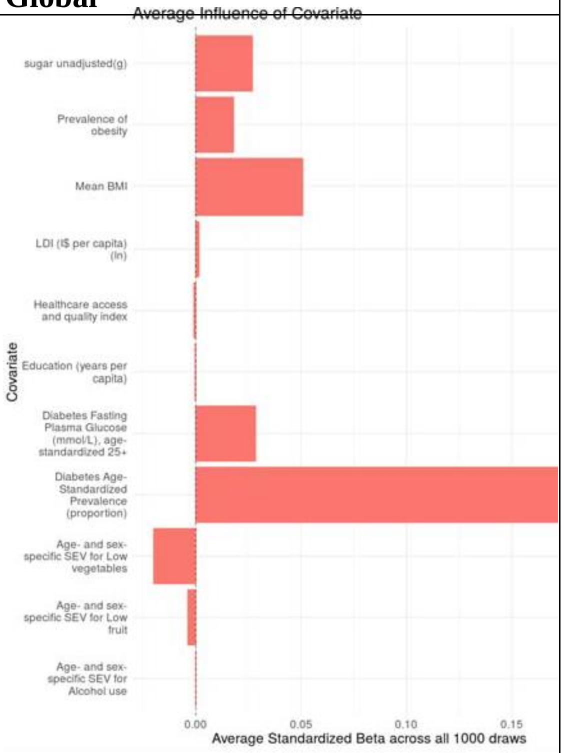
Female	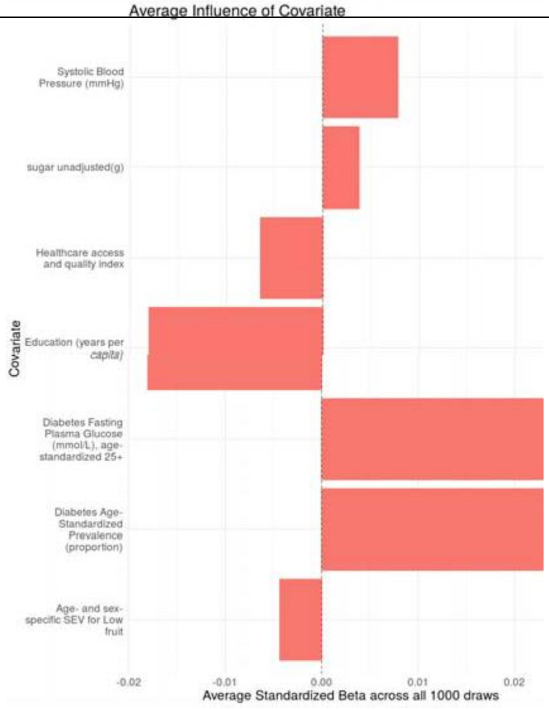	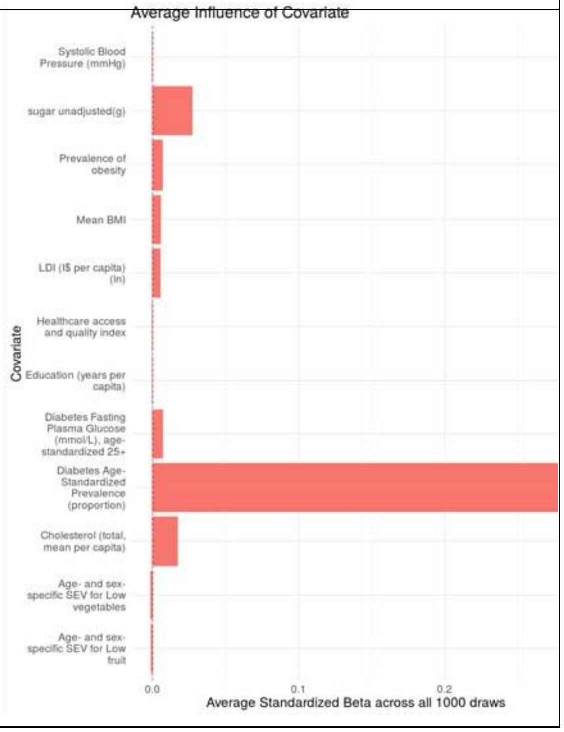

5.3. Chronic Kidney Disease

Flowchart

Chronic Kidney Disease

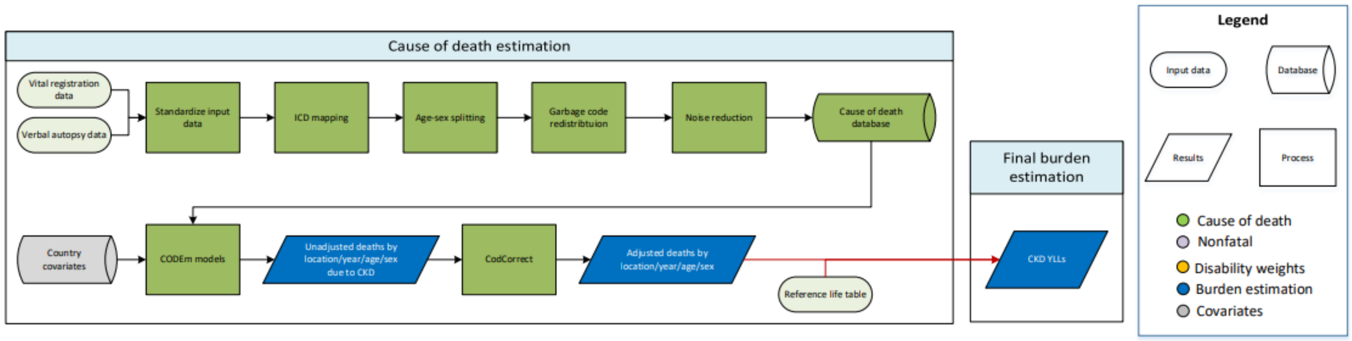

5.3.1. Input data

Vital registration and verbal autopsy data were used to model mortality due to chronic kidney disease. Data were standardised and mapped according to the GBD causes of death ICD mapping method. These data were then age-sex split, and appropriate redistribution of garbage code data was performed. Data points that violated well-established age or time trends or that resulted in extremely high or low cause fractions were marked as outliers and excluded.

5.3.2. Modelling strategy

The estimation strategy used for fatal chronic kidney disease is largely similar to methods used in GBD 2017. A standard CODEm model with location-level covariates was used to model deaths due to chronic kidney disease.

The full list of covariates used in the GBD 2019 model are displayed below.

Level	Covariate	Direction
1	Diabetes fasting plasma glucose (mmol/L)	+
	Diabetes age-standardised prevalence (proportion)	+
	Mean systolic blood pressure (mmHg)	+
	Mean BMI	+
	Healthcare access and quality index	–
2	Mean cholesterol	+
	Total Calories available per capita per day	+
	Red meat unadjusted (kcal per capita)	+
3	Socio-demographic Index	–
	Education (years per capita)	–
	LDI (I$ per capita)	–

5.3.3. Covariate Influences

The following plots show the influence of each covariate on the four CODEm models (male global, male data rich, female global, and female data rich). A positive standardized beta (to the right) means that the covariate was associated with increased death. A negative standardized beta (to the left) means the covariate was associated with decreased death.

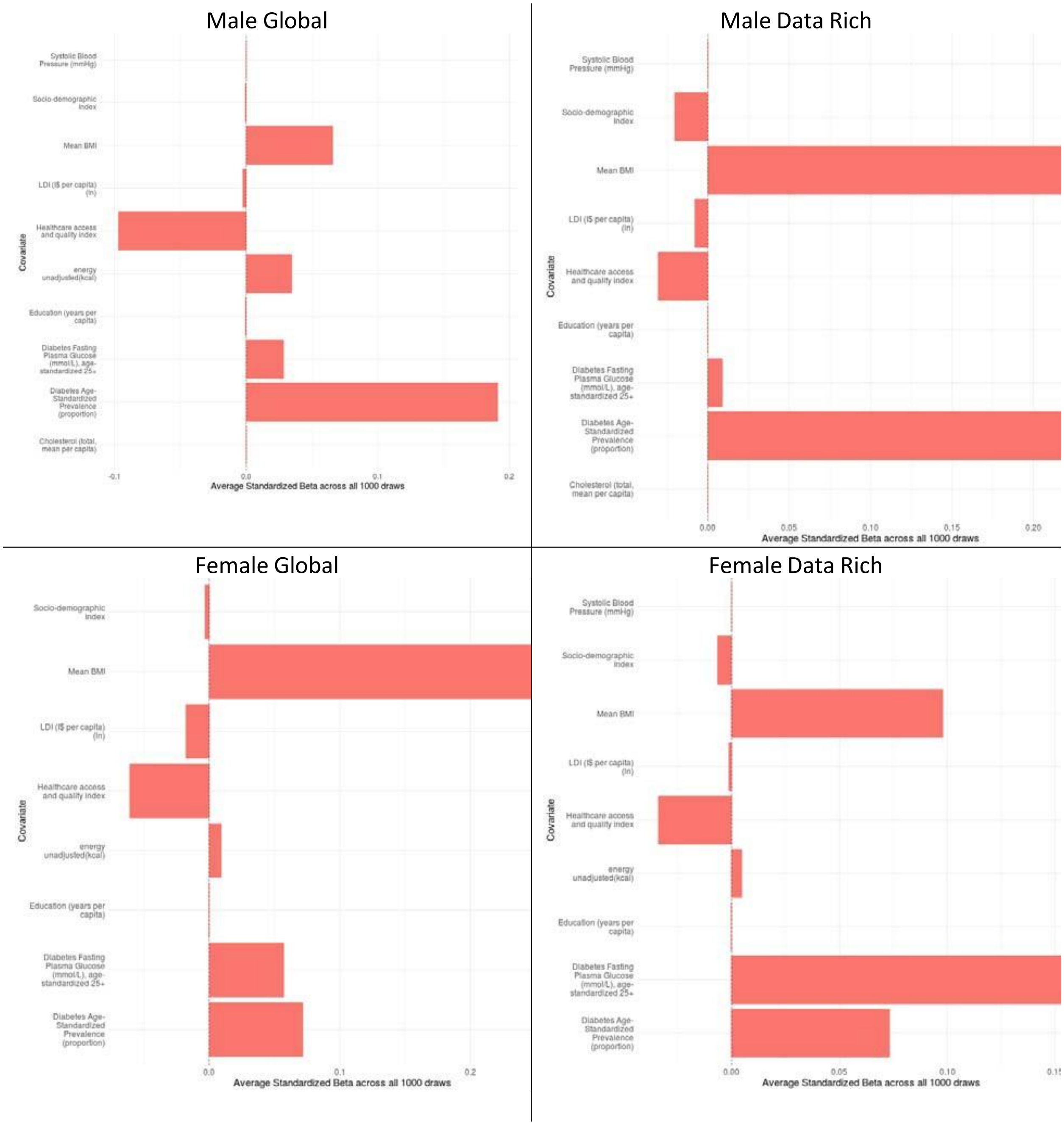

5.4. Chronic Kidney Disease subtypes, including those due to type 1 and type 2 diabetes
Flowchart

Chronic Kidney Disease subtypes

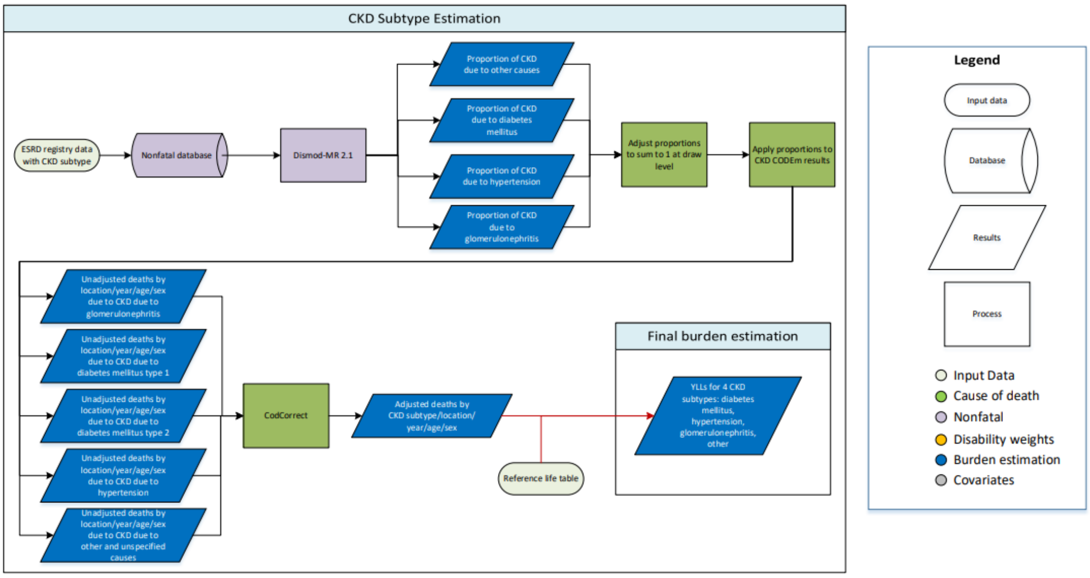

5.4.1. Input data

We estimated deaths due to five subtypes of chronic kidney disease: diabetes mellitus type 1, diabetes mellitus type 2, hypertension, glomerulonephritis, and other causes.

The following codes were used to identify CKD due to diabetes:

Aetiology	ICD 9 Codes	ICD 10 Codes
Type 1 diabetes	250.41, 250.43	E10.2, E10.21, E10.22, E10.29
Type 2 diabetes	250.40, 250.42	E11.2, E11.21, E11.22, E11.29

Deaths due to congenital kidney anomalies (cystic kidney disease and reflux hydronephrosis) were included in the latter category. Data from end-stage renal disease registries were used to estimate proportion of CKD mortality attributable to each CKD subtype. Age-specific data on the proportion of ESRD by subtype was available from the United States, Australia, New Zealand, Nigeria, and Russia.

Vital registration (VR) data were excluded from subtype-specific estimates, as etiology coding in VR sources was considered to be of highly variable quality between countries.

5.4.2. Modelling strategy

We utilized data primarily from end-stage kidney registries that included CKD aetiologies to model CKD-death aetiology proportions.

Data for CKD due to overall diabetes were more widely available than data by type of diabetes. In order to make use of all available data, we modelled the proportion of CKD due to overall diabetes, diabetes type 1, and diabetes type 2. We ran DisMod-MR 2.1 models including diabetes prevalence and mean systolic blood pressure as country-level

covariates to obtain estimates of proportions for each subtype by location, year, age, and sex. Proportion of CKD due to diabetes type 1 and diabetes type 2 were then scaled to sum to the proportion of overall diabetes at the gender, age, and country-matched level. The results from all subtype-specific modelswere adjusted so that estimates across the subtypes equaled 1 at each of 1,000 draws. These adjusted proportions were applied to the parent CKD CODEm model to obtain type-specific estimates of CKD mortality.

6. Socio-demographic Index

We used the GBD Socio-demographic Index (SDI)[2] groups to explore the difference in mortality rates between countries with different levels of development. The SDI is a composite indicator of development status strongly correlated with health outcomes. In short, it is the geometric mean of 0 to 1 indices of total fertility rate in those under 25 years old, mean education for those age 15 years or older, and lag-distributed income per capita (LDI). An index score of 0 represents the minimum level of each covariate input past which selected health outcomes can get no worse, and an index score of 1 represents the maximum level of each covariate input past which selected health outcomes cease to improve. As a composite, a location with an SDI of 0 would have a theoretical minimum level of development relevant to these health outcomes, and a location with an SDI of 1 would have a theoretical maximum level of development relevant to these health outcomes. Detailed information about SDI calculation and the SDI values for each country has been described elsewhere.[2]

7. Universal Health Coverage (UHC) Effective Care Index

As applied in this analysis and explained in greater detail elsewhere,[3] the UHC effective coverage measurement framework involves 30 unique cells from a matrix of five health service types—promotion, prevention, treatment, rehabilitation, and palliation— against five population-age groups (reproductive and newborn, children younger than 5 years, children and adolescents aged 5–19 years, adults aged 20–64 years, and older adults aged ≥65 years). Treatment is sub-divided into two separate groups: first, communicable diseases and maternal, newborn, and child health; and second, non-communicable diseases. Effective coverage indicators were then mapped to these cells to represent needed health services across the life course. Twenty-three effective coverage indicators were included in the present analysis. Data for directly measuring effective intervention coverage are rarely available across health services, locations, and over time. Subsequently, we used viable proxy measures and analytical techniques to approximate effective coverage for conditions considered amenable to health care. Criteria set forth by the WHO 13th General Work Program (GPW13) Expert Reference Group guided selection of effective coverage indicators and preferred measurement approaches. Such criteria stipulated that effective coverage indicators should be currently measurable (i.e., data and methods that support indicator measurement today); reflect differences in effective health services and not factors outside the immediate scope of health systems and UHC (e.g., tobacco taxation and physical infrastructure such as roads and water systems); and use indicators already encompassed within the SDGs and GPW13, or draw from data systems required for monitoring of sustainable development goals (SDGs) and GPW13.

Four effective coverage indicators were measures of intervention coverage and 19 were mortality-based measures to proxy access to quality of care. For the mortality-based measures, we primarily used mortality-to-incidence ratios (MIRs) and mortality-to-prevalence ratios (MPRs) for chronic or longer-term conditions (e.g., diabetes or asthma).

Effective coverage indicators for intervention coverage were kept on their natural scale (0–100%), whereas the 19 other effective coverage indicators were transformed to values on a 0–100 scale. Across locations and from 1990 to 2019, 0 was set by values at the 97·5th percentile or higher (ie, "worst" levels of MIRs) and 100 by the 2·5th percentile or lower (ie, "best" levels of MIRs).

Population-level measures of effective coverage should represent the fraction of total health gains a health system could potentially provide, given currently available interventions, that a health system actually delivers. This construct is thus grounded in the principle of comparability — all health systems ought to maximise potential health gains for their populations — but also requires accounting for local health needs and epidemiological profiles. For instance, if a country currently experiences a high burden of diabetes and a comparatively lower burden of HIV, at least equal or even higher priority in expanding services for diabetes should occur relative to HIV in order to further support health gains

To construct the UHC effective coverage index, we weighted each effective coverage indicator relative to their health gain weights, a metric approximating the population health gains potentially deliverable by health systems for each location-year. In brief, calculations were based on three inputs for each effective coverage indicator and corresponding population-age group: estimates on the 0–100 scale, targeted disease burden, and effectiveness categories of associated interventions or services. For effectiveness, incremental values were assumed by category (i.e., 90% effectiveness for category 1, 70% for category 2, 50% for category 3, and so on).

8. Specific analysis for the creation of the diabetes mortality underage 25 indicator

Country-specific analyses were restricted to those with total population greater than 1 million in 2019 to minimise the higher variability present in the 45 countries with smaller populations.

Analyses specific to this study were done with R version 3.6.

8.1. Aggregating deaths due to diabetes

To create the metric of age-standardised diabetes mortality under age 25 we aggregated deaths due to the GBD cause groups diabetes mellitus, CKD due to diabetes type 1, and CKD due diabetes type 2. For this study, deaths from diabetes were those coded as 250 in ICD-9 or E10–E14 or P70.2 in ICD-10, encompassing both diabetes and chronic kidney disease due to diabetes as defined in the GBD classification.

8.2. Age-standardisation

We age standardized estimates using the direct method, applying age-specific rates to the GBD world population age standard (Appendix Table 1).

Appendix Table 1 – GBD standard world population under age 25

Age group	Percent of population
Early Neonatal	0.08886
Late Neonatal	0.26412
Post Neonatal	4.18093
1 to 4	17.70126
5 to 9	21.41286
10 to 14	20.11906
15 to 19	18.62883
20 to 24	17.60408
Total	100

Appendix Table 2 lists data sources and describes the aggregation of causes in the calculation of age-standardised mortality from diabetes under age 25.

Appendix Table 2. Calculation of mortality from diabetes under age 25 in 2019 using publicly available GBD data as an example of calculations performed to generate estimates. Each age strata´s mortality rates from diabetes, and CKD due to type 1 and type 2 diabetes are summed. This sum is then multiplied by the corresponding strata weight, and the resulting strata contributions to mortality are then summed to produce the total diabetes under 25 mortality rate.

Age group	Percent of population (Weight)	Mortality/100000*				
		Diabetes	CKD due to type 1 diabetes	CKD due to type 2 diabetes	Sum of Causes	Mortality Under 25
Early Neonatal	0.09	1.45	0	0	1.45	0.0013
Late Neonatal	0.26	1.27	0	0	1.27	0.0033
Post Neonatal	4.18	1.12	0.02	0	1.14	0.0477
1 to 4	17.70	0.23	0.01	0	0.24	0.0425
5 to 9	21.41	0.19	0.01	0	0.20	0.0428
10 to 14	20.12	0.22	0.02	0	0.24	0.0483
15 to 19	18.53	0.53	0.08	0.04	0.65	0.1204
20 to 24	17.60	0.81	0.2	0.09	1.1	0.1936
					Total:	0.50

CKD=Chronic kidney disease

*Source: GBD Compare (https://vizhub.healthdata.org/gbd-compare/) with advanced settings in the Patterns view with Sex selected. Display: Cause; Measure: Deaths; Location: Global; Year: 2019; Ages: age-groups at Age-specific slider ; Units: Rate; Sex: Both; Causes: Diabetes, CKD due to 1 diabetes type, CKD due to diabetes type 2. The data can be read off the screen or downloaded using the GBD Results Tool.

9. GATHER Checklist

Checklist of information that should be included in new reports of global health estimates

Item #	Checklist item	Reported on page #
**Objectives and funding**		
**1**	Define the indicator(s), populations (including age, sex, and geographic entities), and time period(s) for which estimates were made.	6
**2**	List the funding sources for the work.	8
**Data Inputs**		
For all data inputs from multiple sources that are synthesized as part of the study:		
**3**	Describe how the data were identified and how the data were accessed.	Methods appendix: Section 3.1
**4**	Specify the inclusion and exclusion criteria. Identify all ad-hoc exclusions.	6
**5**	Provide information on all included data sources and their main characteristics. For each data source used, report reference information or contact name/institution, population represented, data collection method, year(s) of data collection, sex and age range, diagnostic criteria or measurement method, and sample size, as relevant.	Methods appendix: Sections 3.1;<br>5.1.1.1; 5.2.1.1;<br>5.4.1<br>http://ghdx.healthdata.org/gbd-2019
**6**	Identify and describe any categories of input data that have potentially important biases (e.g., based on characteristics listed in item 5).	Methods appendix: Sections 3.1;<br>3.2;<br>5.1.1.1.1; 5.2.1.1;<br>5.4.1
For data inputs that contribute to the analysis but were not synthesized as part of the study:		
**7**	Describe and give sources for any other data inputs.	http://ghdx.healthdata.org/gbd-2019
For all data inputs:		
**8**	Provide all data inputs in a file format from which data can be efficiently extracted (e.g., a spreadsheet rather than a PDF), including all relevant meta-data listed in item 5. For any data inputs that cannot be shared because of ethical or legal reasons, such as third-party ownership, provide a contact name or the name of the institution that retains the right to the data.	http://ghdx.healthdata.org<br><br>https://vizhub.healthdata.org/gbd-compare/
**Data analysis**		
**9**	Provide a conceptual overview of the data analysis method. A diagram may be helpful.	Main text Methods: 6-8;<br><br>Methods appendix: Sections 3.1;<br>4;<br>5.1.1.1.1; 5.2.1.1;<br>5.4.1
**10**	Provide a detailed description of all steps of the analysis, including mathematical formulae. This description should cover, as relevant, data cleaning, data pre-processing, data adjustments and weighting of data sources, and mathematical or statistical model(s).	Methods appendix: Sections 3.1;<br>4;<br>5.1.1.1.1; 5.2.1.1;<br>5.4.1<br>8
**11**	Describe how candidate models were evaluated and how the final model(s) were selected.	Methods appendix: Sections 3.1;<br>4;<br>5.1.1.1.2;<br>5.2.1.2;<br>5.3.2;<br>5.4.2;<br>8

**12**	Provide the results of an evaluation of model performance, if done, as well as the results of any relevant sensitivity analysis.	Methods appendix: Sections 4;<br>5.1.1.1.4; 5.2.1.4;<br>5.3.3
**13**	Describe methods for calculating uncertainty of the estimates. State which sources of uncertainty were, and were not, accounted for in the uncertainty analysis.	Methods appendix: Section 4
**14**	State how analytic or statistical source code used to generate estimates can be accessed.	Code is provided in an online repository https://github.com/ihmeuw/ihme-modeling/tree/main/gbd_2019
**Results and Discussion**		
**15**	Provide published estimates in a file format from which data can be efficiently extracted.	Main text, methods appendix, and online<br>data tools (data visualization tools, data<br>query tools, and the Global Health Data<br>Exchange, http://ghdx.healthdata.org
**16**	Report a quantitative measure of the uncertainty of the estimates (e.g. uncertainty intervals).	8-10
**17**	Interpret results in light of existing evidence. If updating a previous set of estimates, describe the reasons for changes in estimates.	10-13
**18**	Discuss limitations of the estimates. Include a discussion of any modelling assumptions or data limitations that affect interpretation of the estimates.	12-13

This checklist should be used in conjunction with the GATHER statement and Explanation and Elaboration document, found on gather-statement.org

References

1 Vos T, Lim SS, Abbafati C, *et al.* Global burden of 369 diseases and injuries in 204 countries and territories, 1990–2019: a systematic analysis for the Global Burden of Disease Study 2019. *The Lancet* 2020; **396**: 1204–22.

2 Wang H, Abbas KM, Abbasifard M, *et al.* Global age-sex-specific fertility, mortality, healthy life expectancy (HALE), and population estimates in 204 countries and territories, 1950–2019: a comprehensive demographic analysis for the Global Burden of Disease Study 2019. *The Lancet* 2020; **396**: 1160–203.

3 Lozano R, Fullman N, Mumford JE, *et al.* Measuring universal health coverage based on an index of effective coverage of health services in 204 countries and territories, 1990–2019: a systematic analysis for the Global Burden of Disease Study 2019. *The Lancet* 2020; **396**: 1250–84.

4 Stevens GA, Alkema L, Black RE, *et al.* Guidelines for accurate and transparent health estimates reporting: the GATHER statement. *PLoS Med* 2016; **13**: e1002056.

5 Dicker D, Nguyen G, Abate D, *et al.* Global, regional, and national age-sex-specific mortality and life expectancy, 1950–2017: a systematic analysis for the Global Burden of Disease Study 2017. *The lancet* 2018; **392**: 1684–735.

6 Johnson SC, Cunningham M, Dippenaar IN, *et al.* Public health utility of cause of death data: applying empirical algorithms to improve data quality. *BMC Med Inform Decis Mak* 2021; **21**: 1–20.

7 Lozano R, Naghavi M, Foreman K, *et al.* Global and regional mortality from 235 causes of death for 20 age groups in 1990 and 2010: a systematic analysis for the Global Burden of Disease Study 2010. *The lancet* 2012; **380**: 2095–128.

8 Foreman KJ, Lozano R, Lopez AD, Murray CJ. Modeling causes of death: an integrated approach using CODEm. *Popul Health Metr* 2012; **10**: 1–23.

9 Bell RM, Koren Y. Lessons from the netflix prize challenge. *Acm Sigkdd Explor Newsl* 2007; **9**: 75–9.

10 Bell RM, Koren Y, Volinsky C. All together now: A perspective on the netflix prize. *Chance* 2010; **23**: 24–9.

Additional Results in Tables and Figures

Supplementary Table 1 – List of countries by SDI quintile.

Supplementary Table 2 - Age-standardised deaths rate (95% Uncertainty interval) due to diabetes under age 25, 1990 and 2019 , percentage change in rates 1990-2019, percent of total deaths, number of deaths, and population.

Supplementary Figure 1 – Map showing SDI quintile for each country.

Supplementary Figure 2: Diabetes (type 1 and type 2 combined) mortality rates under age 25 by Socio-demographic Index (SDI) groups A) Trends in age-standardised mortality rate from 1990 to 2019 B) Age-specific mortality in 2019.

Supplementary Figure 3: Age-standardised mortality rate (per 100,000) due to type 1 diabetes under age 25 in 2019. Countries with total population <1 million are excluded.

Supplementary Table 1 - Countries by SDI quintile	
**Location**	**SDI quintile**
Andorra	High SDI
Australia	High SDI
Austria	High SDI
Belgium	High SDI
Bermuda	High SDI
Brunei	High SDI
Canada	High SDI
Cyprus	High SDI
Czech Republic	High SDI
Denmark	High SDI
Estonia	High SDI
Finland	High SDI
France	High SDI
Germany	High SDI
Guam	High SDI
Iceland	High SDI
Ireland	High SDI
Japan	High SDI
Kuwait	High SDI
Latvia	High SDI
Lithuania	High SDI
Luxembourg	High SDI
Monaco	High SDI
Netherlands	High SDI
New Zealand	High SDI
Norway	High SDI
Puerto Rico	High SDI
Qatar	High SDI
San Marino	High SDI
Saudi Arabia	High SDI
Singapore	High SDI
Slovakia	High SDI
Slovenia	High SDI
South Korea	High SDI
Sweden	High SDI
Switzerland	High SDI
Taiwan (province of China)	High SDI
UK	High SDI
United Arab Emirates	High SDI
USA	High SDI
American Samoa	High-middle SDI
Antigua and Barbuda	High-middle SDI
Argentina	High-middle SDI
Bahrain	High-middle SDI
Barbados	High-middle SDI

Belarus	High-middle SDI
Bosnia and Herzegovina	High-middle SDI
Bulgaria	High-middle SDI
Chile	High-middle SDI
Cook Islands	High-middle SDI
Croatia	High-middle SDI
Dominica	High-middle SDI
Georgia	High-middle SDI
Greece	High-middle SDI
Greenland	High-middle SDI
Hungary	High-middle SDI
Israel	High-middle SDI
Italy	High-middle SDI
Jordan	High-middle SDI
Kazakhstan	High-middle SDI
Lebanon	High-middle SDI
Libya	High-middle SDI
Malaysia	High-middle SDI
Malta	High-middle SDI
Mauritius	High-middle SDI
Moldova	High-middle SDI
Montenegro	High-middle SDI
Niue	High-middle SDI
North Macedonia	High-middle SDI
Northern Mariana Islands	High-middle SDI
Oman	High-middle SDI
Palau	High-middle SDI
Poland	High-middle SDI
Portugal	High-middle SDI
Romania	High-middle SDI
Russia	High-middle SDI
Saint Kitts and Nevis	High-middle SDI
Serbia	High-middle SDI
Seychelles	High-middle SDI
Spain	High-middle SDI
Sri Lanka	High-middle SDI
The Bahamas	High-middle SDI
Trinidad and Tobago	High-middle SDI
Turkey	High-middle SDI
Ukraine	High-middle SDI
Uruguay	High-middle SDI
Virgin Islands	High-middle SDI
Albania	Middle SDI
Algeria	Middle SDI
Armenia	Middle SDI
Azerbaijan	Middle SDI
Botswana	Middle SDI

Brazil	Middle SDI
China	Middle SDI
Colombia	Middle SDI
Costa Rica	Middle SDI
Cuba	Middle SDI
Ecuador	Middle SDI
Egypt	Middle SDI
Equatorial Guinea	Middle SDI
Fiji	Middle SDI
Gabon	Middle SDI
Grenada	Middle SDI
Guyana	Middle SDI
Indonesia	Middle SDI
Iran	Middle SDI
Iraq	Middle SDI
Jamaica	Middle SDI
Mexico	Middle SDI
Namibia	Middle SDI
Nauru	Middle SDI
Panama	Middle SDI
Paraguay	Middle SDI
Peru	Middle SDI
Philippines	Middle SDI
Saint Lucia	Middle SDI
Saint Vincent and the Grenadines	Middle SDI
Samoa	Middle SDI
South Africa	Middle SDI
Suriname	Middle SDI
Syria	Middle SDI
Thailand	Middle SDI
Tokelau	Middle SDI
Tonga	Middle SDI
Tunisia	Middle SDI
Turkmenistan	Middle SDI
Uzbekistan	Middle SDI
Vietnam	Middle SDI
Angola	Low-middle SDI
Bangladesh	Low-middle SDI
Belize	Low-middle SDI
Bhutan	Low-middle SDI
Bolivia	Low-middle SDI
Cambodia	Low-middle SDI
Cameroon	Low-middle SDI
Cape Verde	Low-middle SDI
Congo (Brazzaville)	Low-middle SDI
Djibouti	Low-middle SDI
Dominican Republic	Low-middle SDI

El Salvador	Low-middle SDI
eSwatini	Low-middle SDI
Federated States of Micronesia	Low-middle SDI
Ghana	Low-middle SDI
Guatemala	Low-middle SDI
Honduras	Low-middle SDI
India	Low-middle SDI
Kenya	Low-middle SDI
Kiribati	Low-middle SDI
Kyrgyzstan	Low-middle SDI
Laos	Low-middle SDI
Lesotho	Low-middle SDI
Maldives	Low-middle SDI
Marshall Islands	Low-middle SDI
Mauritania	Low-middle SDI
Mongolia	Low-middle SDI
Morocco	Low-middle SDI
Myanmar	Low-middle SDI
Nicaragua	Low-middle SDI
Nigeria	Low-middle SDI
North Korea	Low-middle SDI
Palestine	Low-middle SDI
SÃ£o TomÃ© and PrÃncipe	Low-middle SDI
Sudan	Low-middle SDI
Tajikistan	Low-middle SDI
Timor-Leste	Low-middle SDI
Tuvalu	Low-middle SDI
Vanuatu	Low-middle SDI
Venezuela	Low-middle SDI
Zambia	Low-middle SDI
Zimbabwe	Low-middle SDI
Afghanistan	Low SDI
Benin	Low SDI
Burkina Faso	Low SDI
Burundi	Low SDI
CÃ´te d'Ivoire	Low SDI
Central African Republic	Low SDI
Chad	Low SDI
Comoros	Low SDI
DR Congo	Low SDI
Eritrea	Low SDI
Ethiopia	Low SDI
Guinea	Low SDI
Guinea-Bissau	Low SDI
Haiti	Low SDI
Liberia	Low SDI
Madagascar	Low SDI

Malawi	Low SDI
Mali	Low SDI
Mozambique	Low SDI
Nepal	Low SDI
Niger	Low SDI
Pakistan	Low SDI
Papua New Guinea	Low SDI
Rwanda	Low SDI
Senegal	Low SDI
Sierra Leone	Low SDI
Solomon Islands	Low SDI
Somalia	Low SDI
South Sudan	Low SDI
Tanzania	Low SDI
The Gambia	Low SDI
Togo	Low SDI
Uganda	Low SDI
Yemen	Low SDI

Supplementary Table 2 - Age-standardised deaths rate (95% Uncertainty interval) due to diabetes under age 25, 1990 and 2019 , percentage change in rates 1990-2019, percent of total deaths, number of deaths, and population.

	Age-standardised deaths rate (per 100,000)									Percent of total deaths			Number of deaths due to DM			Total Population	Population under 25 years
	1990			2019			Percentage Change 1990-2019			2019			2019			2019	2019
**Location**	**mean**	**lower**	**upper**	**mean**	**lower**	**upper**	**mean**	**lower**	**upper**	**mean**	**lower**	**upper**	**mean**	**lower**	**upper**		
**Global**	**0.60**	**0.51**	**0.69**	**0.50**	**0.44**	**0.58**	**-17.0%**	**-28.4%**	**-2.9%**	**0.2%**	**0.2%**	**0.3%**	**16306**	**14192**	**18936**	**7,737,464,623**	**3,179,418,532**
**Low SDI**	**0.83**	**0.70**	**0.95**	**0.71**	**0.60**	**0.86**	**-13.6%**	**-28.4%**	**3.4%**	**0.1%**	**0.1%**	**0.2%**	**4864**	**4074**	**5898**	**1,128,676,637**	**700,553,830**
**Low-middle SDI**	**0.71**	**0.60**	**0.84**	**0.60**	**0.51**	**0.70**	**-15.4%**	**-30.9%**	**2.6%**	**0.3%**	**0.2%**	**0.3%**	**5298**	**4509**	**6202**	**1,763,982,420**	**857,237,934**
**Middle SDI**	**0.70**	**0.56**	**0.83**	**0.48**	**0.41**	**0.57**	**-31.3%**	**-40.2%**	**-15.4%**	**0.4%**	**0.3%**	**0.5%**	**4709**	**3996**	**5639**	**2,396,565,865**	**918,894,779**
**High-middle SDI**	**0.39**	**0.34**	**0.45**	**0.22**	**0.19**	**0.25**	**-44.5%**	**-52.0%**	**-36.5%**	**0.3%**	**0.3%**	**0.4%**	**1000**	**878**	**1136**	**1,430,403,837**	**418,213,712**
**High SDI**	**0.19**	**0.17**	**0.20**	**0.13**	**0.12**	**0.14**	**-29.4%**	**-33.3%**	**-25.4%**	**0.3%**	**0.3%**	**0.4%**	**415**	**390**	**443**	**1,013,384,784**	**282,545,209**
**Central Europe, eastern Europe, and central Asia**	**0.29**	**0.28**	**0.31**	**0.32**	**0.28**	**0.35**	**7.9%**	**-2.8%**	**19.5%**	**0.4%**	**0.3%**	**0.4%**	**415**	**369**	**464**	**417,725,139**	**128,780,706**
**Central Asia**	**0.43**	**0.40**	**0.49**	**0.62**	**0.53**	**0.72**	**43.5%**	**22.8%**	**66.9%**	**0.4%**	**0.4%**	**0.5%**	**257**	**219**	**298**	**93,530,807**	**41,508,906**
Armenia	0.29	0.25	0.34	0.32	0.26	0.39	11.3%	-14.1%	41.1%	0.47%	0.37%	0.57%	3	3	4	3,019,674	963,847
Azerbaijan	0.51	0.44	0.61	0.63	0.49	0.78	22.1%	-7.7%	56.9%	0.43%	0.33%	0.54%	25	20	32	10,278,674	3,788,141
Georgia	0.28	0.25	0.34	0.36	0.29	0.43	25.4%	1.9%	57.7%	0.46%	0.38%	0.56%	4	3	5	3,664,752	1,133,212
Kazakhstan	0.46	0.41	0.52	0.33	0.27	0.39	-29.3%	-40.9%	-15.2%	0.32%	0.26%	0.38%	23	20	28	18,392,068	7,515,500
Kyrgyzstan	0.37	0.31	0.46	0.23	0.19	0.28	-39.3%	-49.1%	-27.0%	0.20%	0.16%	0.24%	7	6	9	6,535,459	3,184,484
Mongolia	0.41	0.32	0.54	0.30	0.21	0.41	-26.6%	-48.9%	7.1%	0.18%	0.14%	0.24%	4	3	5	3,387,589	1,475,723
Tajikistan	0.49	0.42	0.57	0.75	0.55	0.96	52.7%	10.0%	107.8%	0.36%	0.27%	0.46%	37	27	47	9,492,414	5,000,865
Turkmenistan	0.60	0.52	0.70	0.92	0.74	1.15	54.2%	21.9%	94.7%	0.52%	0.42%	0.61%	21	17	26	5,083,080	2,326,454
Uzbekistan	0.41	0.36	0.48	0.81	0.66	0.99	96.3%	57.0%	137.7%	0.56%	0.46%	0.67%	132	107	161	33,677,096	16,120,679
**Central Europe**	**0.19**	**0.17**	**0.20**	**0.10**	**0.08**	**0.11**	**-48.2%**	**-56.0%**	**-39.7%**	**0.3%**	**0.2%**	**0.3%**	**31**	**27**	**36**	**114,223,622**	**29,546,514**
Albania	0.25	0.19	0.29	0.15	0.12	0.20	-38.1%	-56.1%	-11.1%	0.19%	0.15%	0.24%	2	1	2	2,720,353	882,894
Bosnia and Herzegovina	0.15	0.13	0.18	0.25	0.19	0.33	68.1%	20.3%	132.2%	0.78%	0.65%	0.92%	3	2	4	3,299,982	877,293
Bulgaria	0.37	0.32	0.41	0.27	0.21	0.35	-25.4%	-42.9%	-3.3%	0.48%	0.41%	0.55%	5	4	6	6,934,625	1,610,771
Croatia	0.07	0.06	0.08	0.04	0.03	0.05	-42.6%	-56.2%	-24.8%	0.15%	0.13%	0.17%	1	0	1	4,247,902	1,071,055
Czech Republic	0.11	0.10	0.12	0.09	0.07	0.11	-17.0%	-36.7%	5.5%	0.32%	0.28%	0.36%	2	2	3	10,643,487	2,650,576
Hungary	0.12	0.11	0.14	0.07	0.06	0.09	-42.9%	-55.5%	-26.3%	0.24%	0.21%	0.28%	2	1	2	9,674,413	2,388,659
Montenegro	0.25	0.21	0.30	0.18	0.14	0.23	-29.6%	-46.9%	-6.3%	0.57%	0.46%	0.69%	0	0	0	620,340	190,652
North Macedonia	0.22	0.19	0.26	0.18	0.14	0.24	-19.6%	-40.9%	8.8%	0.39%	0.32%	0.47%	1	1	2	2,152,731	611,218
Poland	0.16	0.15	0.18	0.07	0.06	0.08	-58.4%	-64.3%	-51.6%	0.20%	0.17%	0.22%	7	6	8	38,434,445	9,786,583
Romania	0.24	0.21	0.27	0.10	0.08	0.12	-58.2%	-66.0%	-48.4%	0.19%	0.16%	0.22%	5	4	6	19,237,066	5,041,746

Serbia	0.22	0.18	0.27	0.07	0.06	0.09	-66.4%	-76.5%	-53.9%	0.27%	0.23%	0.33%	2	2	3	8,746,785	2,521,950
Slovakia	0.13	0.12	0.15	0.07	0.05	0.09	-46.6%	-60.5%	-30.2%	0.18%	0.15%	0.21%	1	1	1	5,437,223	1,411,747
Slovenia	0.06	0.05	0.07	0.03	0.03	0.04	-46.1%	-57.1%	-33.3%	0.13%	0.11%	0.15%	0	0	0	2,074,271	501,371
**Eastern Europe**	**0.30**	**0.28**	**0.31**	**0.22**	**0.20**	**0.24**	**-25.6%**	**-33.1%**	**-17.4%**	**0.3%**	**0.3%**	**0.3%**	**127**	**114**	**140**	**209,970,710**	**57,725,286**
Belarus	0.26	0.23	0.30	0.08	0.06	0.11	-69.8%	-78.1%	-57.7%	0.15%	0.13%	0.19%	2	1	3	9,500,785	2,549,760
Estonia	0.25	0.22	0.28	0.18	0.14	0.23	-25.3%	-43.8%	-3.4%	0.53%	0.46%	0.61%	1	0	1	1,312,361	340,793
Latvia	0.28	0.25	0.31	0.27	0.22	0.34	-4.0%	-24.5%	22.9%	0.61%	0.52%	0.70%	1	1	2	1,915,292	478,102
Lithuania	0.24	0.21	0.27	0.15	0.12	0.18	-36.4%	-49.6%	-20.8%	0.38%	0.32%	0.44%	1	1	1	2,794,223	717,594
Moldova	0.35	0.31	0.40	0.16	0.14	0.19	-54.1%	-63.1%	-42.6%	0.25%	0.20%	0.30%	2	1	2	3,688,191	982,271
Russia	0.30	0.28	0.32	0.19	0.16	0.21	-37.1%	-44.3%	-29.2%	0.27%	0.24%	0.30%	76	66	86	146,717,427	41,479,062
Ukraine	0.30	0.27	0.33	0.37	0.30	0.45	23.2%	-0.3%	52.7%	0.41%	0.35%	0.50%	44	36	54	44,042,431	11,177,705
**High income**	**0.16**	**0.15**	**0.18**	**0.12**	**0.11**	**0.13**	**-25.6%**	**-29.0%**	**-21.9%**	**0.3%**	**0.3%**	**0.3%**	**410**	**388**	**434**	**1,083,976,063**	**306,973,375**
**Australasia**	**0.12**	**0.11**	**0.13**	**0.07**	**0.07**	**0.08**	**-37.2%**	**-44.5%**	**-28.4%**	**0.2%**	**0.2%**	**0.3%**	**7**	**6**	**8**	**29,063,781**	**9,084,298**
Australia	0.12	0.10	0.13	0.08	0.07	0.09	-35.3%	-43.9%	-24.5%	0.24%	0.21%	0.27%	6	5	7	24,568,113	7,609,303
New Zealand	0.12	0.11	0.14	0.07	0.06	0.07	-45.9%	-53.3%	-37.3%	0.17%	0.15%	0.19%	1	1	1	4,495,667	1,474,995
**High-income Asia Pacific**	**0.15**	**0.14**	**0.16**	**0.05**	**0.05**	**0.06**	**-65.4%**	**-68.6%**	**-62.0%**	**0.2%**	**0.2%**	**0.3%**	**25**	**23**	**28**	**187,291,233**	**42,175,058**
Brunei	1.14	0.90	1.45	0.53	0.43	0.66	-53.3%	-64.6%	-39.0%	0.94%	0.74%	1.17%	1	1	1	437,119	170,549
Japan	0.10	0.09	0.11	0.04	0.03	0.04	-63.6%	-66.2%	-58.7%	0.18%	0.17%	0.21%	11	11	13	127,788,411	27,773,561
South Korea	0.24	0.22	0.27	0.08	0.07	0.09	-68.6%	-73.8%	-62.8%	0.35%	0.30%	0.41%	12	10	14	53,398,252	12,915,671
Singapore	0.15	0.11	0.18	0.04	0.03	0.05	-73.5%	-79.6%	-63.2%	0.21%	0.15%	0.29%	1	0	1	5,667,451	1,315,277
**High-income North America**	**0.21**	**0.20**	**0.23**	**0.20**	**0.19**	**0.21**	**-4.4%**	**-11.6%**	**0.7%**	**0.4%**	**0.4%**	**0.4%**	**248**	**235**	**262**	**364,560,550**	**113,747,120**
Canada	0.13	0.12	0.15	0.10	0.09	0.12	-22.7%	-33.1%	-10.8%	0.27%	0.24%	0.32%	11	10	13	36,519,840	10,328,744
Greenland	0.16	0.12	0.21	0.07	0.05	0.10	-54.0%	-72.6%	-28.4%	0.09%	0.07%	0.11%	0	0	0	56,188	19,375
USA	0.21	0.20	0.24	0.21	0.19	0.22	-3.5%	-10.5%	2.0%	0.39%	0.37%	0.41%	236	223	249	327,978,730	103,397,194
**Southern Latin America**	**0.24**	**0.22**	**0.26**	**0.18**	**0.17**	**0.21**	**-23.1%**	**-31.4%**	**-13.3%**	**0.3%**	**0.2%**	**0.3%**	**50**	**45**	**56**	**66,753,135**	**25,182,188**
Argentina	0.30	0.26	0.33	0.22	0.20	0.25	-25.7%	-35.1%	-14.1%	0.29%	0.25%	0.33%	42	37	47	45,115,284	17,742,952
Chile	0.12	0.11	0.14	0.09	0.08	0.10	-25.6%	-36.2%	-13.2%	0.19%	0.16%	0.22%	6	5	7	18,198,359	6,229,948
Uruguay	0.22	0.19	0.25	0.18	0.15	0.20	-19.7%	-32.2%	-4.4%	0.28%	0.23%	0.33%	2	2	3	3,436,137	1,208,023
**Western Europe**	**0.13**	**0.11**	**0.13**	**0.06**	**0.06**	**0.07**	**-49.4%**	**-53.3%**	**-42.8%**	**0.2%**	**0.2%**	**0.3%**	**80**	**75**	**87**	**436,307,365**	**116,784,712**
Andorra	0.07	0.05	0.09	0.04	0.03	0.05	-43.2%	-63.1%	-14.6%	0.18%	0.14%	0.24%	0	0	0	83,064	19,199
Austria	0.09	0.08	0.10	0.06	0.05	0.07	-29.4%	-39.4%	-17.9%	0.23%	0.20%	0.26%	2	1	2	8,916,185	2,279,360
Belgium	0.13	0.11	0.14	0.05	0.04	0.06	-61.2%	-67.7%	-52.1%	0.17%	0.14%	0.20%	2	1	2	11,419,166	3,220,466
Cyprus	0.05	0.04	0.08	0.03	0.02	0.04	-45.9%	-58.6%	-29.2%	0.08%	0.07%	0.12%	0	0	0	1,313,477	361,812
Denmark	0.13	0.12	0.15	0.10	0.09	0.12	-23.4%	-37.6%	-6.5%	0.46%	0.39%	0.54%	2	2	2	5,802,733	1,688,353

Finland	0.31	0.27	0.36	0.15	0.13	0.17	-53.2%	-62.8%	-41.1%	0.61%	0.52%	0.71%	2	2	3	5,534,095	1,483,865
France	0.08	0.07	0.09	0.05	0.04	0.06	-36.3%	-46.0%	-24.4%	0.17%	0.15%	0.20%	10	9	11	66,204,315	19,757,887
Germany	0.12	0.11	0.13	0.06	0.06	0.07	-46.9%	-54.6%	-38.0%	0.26%	0.23%	0.29%	15	13	17	84,914,056	20,554,281
Greece	0.07	0.06	0.08	0.04	0.03	0.05	-44.2%	-52.5%	-34.7%	0.12%	0.11%	0.15%	1	1	1	10,337,172	2,481,924
Iceland	0.05	0.04	0.06	0.03	0.02	0.04	-42.7%	-55.5%	-26.1%	0.13%	0.11%	0.15%	0	0	0	344,876	112,466
Ireland	0.10	0.09	0.11	0.05	0.04	0.05	-53.5%	-61.8%	-44.0%	0.17%	0.15%	0.20%	1	1	1	4,910,357	1,629,276
Israel	0.07	0.06	0.09	0.05	0.04	0.06	-30.0%	-43.7%	-16.3%	0.17%	0.14%	0.19%	2	2	2	9,309,583	3,977,742
Italy	0.14	0.11	0.15	0.06	0.05	0.06	-60.1%	-65.8%	-51.4%	0.23%	0.21%	0.26%	8	7	9	60,313,170	13,794,297
Luxembourg	0.10	0.09	0.12	0.05	0.04	0.06	-55.5%	-65.3%	-43.4%	0.18%	0.16%	0.21%	0	0	0	618,550	170,655
Malta	0.16	0.14	0.19	0.12	0.10	0.14	-27.6%	-40.0%	-12.2%	0.32%	0.26%	0.38%	0	0	0	439,221	108,397
Monaco	0.05	0.04	0.07	0.04	0.03	0.05	-21.8%	-44.3%	6.9%	0.15%	0.12%	0.19%	0	0	0	37,572	8,553
Netherlands	0.12	0.10	0.13	0.05	0.04	0.06	-58.0%	-64.9%	-47.7%	0.20%	0.17%	0.23%	3	2	3	17,156,788	4,822,100
Norway	0.36	0.31	0.38	0.18	0.16	0.19	-50.7%	-55.3%	-43.5%	0.79%	0.72%	0.84%	3	3	3	5,348,847	1,597,426
Portugal	0.19	0.16	0.21	0.06	0.06	0.07	-65.7%	-71.7%	-58.1%	0.27%	0.23%	0.31%	2	2	2	10,651,263	2,538,228
San Marino	0.07	0.05	0.09	0.05	0.03	0.07	-29.8%	-56.3%	9.8%	0.17%	0.14%	0.21%	0	0	0	33,100	9,494
Spain	0.12	0.10	0.14	0.04	0.03	0.05	-69.7%	-75.7%	-59.6%	0.15%	0.13%	0.20%	4	3	5	46,021,218	11,238,120
Sweden	0.15	0.13	0.16	0.11	0.10	0.12	-27.2%	-37.0%	-16.6%	0.45%	0.40%	0.50%	3	3	4	10,222,546	2,933,651
Switzerland	0.10	0.08	0.11	0.03	0.03	0.04	-64.8%	-70.9%	-57.1%	0.1%	0.1%	0.2%	1	1	1	8,775,204	2,241,415
UK	0.17	0.15	0.18	0.09	0.09	0.10	-46.1%	-49.5%	-38.2%	0.3%	0.3%	0.3%	20	19	21	67,220,447	19,653,934
**Latin America and Caribbean**	**0.72**	**0.62**	**0.79**	**0.52**	**0.44**	**0.61**	**-28.2%**	**-37.6%**	**-17.1%**	**0.40%**	**0.36%**	**0.47%**	**1340**	**1151**	**1555**	**584,378,201**	**242,623,036**
**Andean Latin America**	**0.36**	**0.31**	**0.42**	**0.29**	**0.22**	**0.36**	**-19.8%**	**-37.4%**	**1.1%**	**0.23%**	**0.19%**	**0.26%**	**87**	**68**	**109**	**63,595,539**	**29,221,125**
Bolivia	0.59	0.45	0.75	0.37	0.26	0.50	-37.2%	-56.9%	-12.6%	0.18%	0.14%	0.23%	22	16	30	12,011,750	6,060,596
Ecuador	0.35	0.29	0.41	0.39	0.30	0.50	12.0%	-14.0%	44.8%	0.3%	0.3%	0.4%	34	27	44	17,588,392	8,292,012
Peru	0.29	0.24	0.35	0.19	0.13	0.27	-33.5%	-53.9%	-5.4%	0.19%	0.15%	0.24%	30	21	42	33,995,397	14,868,517
**Caribbean**	**1.04**	**0.88**	**1.23**	**0.96**	**0.75**	**1.21**	**-7.7%**	**-29.1%**	**16.2%**	**0.43%**	**0.36%**	**0.51%**	**193**	**152**	**243**	**47,166,960**	**19,328,477**
Antigua and Barbuda	0.81	0.69	0.94	0.69	0.55	0.85	-14.5%	-32.2%	12.2%	1.11%	0.94%	1.32%	0	0	0	88,489	30,917
The Bahamas	0.71	0.60	0.84	0.71	0.55	0.91	-0.2%	-23.6%	31.5%	0.75%	0.62%	0.88%	1	1	1	376,940	141,544
Barbados	1.01	0.88	1.15	0.82	0.65	1.04	-18.9%	-37.7%	4.9%	1.18%	1.02%	1.39%	1	1	1	297,771	88,364
Belize	1.04	0.82	1.23	0.94	0.78	1.13	-9.0%	-28.2%	16.4%	0.85%	0.72%	0.99%	2	2	3	410,094	207,024
Bermuda	0.36	0.29	0.48	0.21	0.16	0.28	-42.2%	-54.9%	-24.9%	0.56%	0.47%	0.72%	0	0	0	64,030	14,952
Cuba	0.41	0.37	0.46	0.15	0.11	0.19	-64.8%	-72.8%	-54.6%	0.41%	0.34%	0.49%	5	4	7	11,358,510	3,190,363
Dominica	1.09	0.91	1.32	1.15	0.84	1.56	4.9%	-25.7%	44.3%	0.86%	0.70%	1.04%	0	0	0	68,681	25,532
Dominican Republic	1.10	0.90	1.30	0.95	0.65	1.30	-14.3%	-41.5%	17.7%	0.57%	0.44%	0.72%	50	34	69	10,881,855	4,994,855
Grenada	1.28	1.07	1.51	0.91	0.75	1.10	-28.3%	-44.0%	-10.2%	1.17%	0.92%	1.48%	0	0	1	103,215	40,499
Guyana	1.57	1.25	1.87	1.51	1.13	1.98	-3.8%	-30.9%	33.8%	0.93%	0.80%	1.09%	6	5	8	770,705	362,583

Haiti	2.39	1.71	3.29	1.57	1.14	2.10	-34.1%	-56.0%	-7.5%	0.35%	0.26%	0.46%	103	75	139	12,402,099	6,616,190
Jamaica	0.47	0.40	0.55	0.52	0.39	0.68	10.1%	-15.8%	44.4%	0.75%	0.63%	0.89%	7	5	9	2,810,754	1,132,656
Puerto Rico	0.40	0.35	0.46	0.33	0.24	0.43	-18.4%	-40.8%	9.8%	0.59%	0.50%	0.69%	4	3	5	3,521,431	984,521
Saint Kitts and Nevis	0.81	0.70	0.93	0.60	0.43	0.79	-25.4%	-47.7%	-3.1%	0.65%	0.46%	0.83%	0	0	0	59,508	20,690
Saint Lucia	1.07	0.92	1.23	0.82	0.66	1.02	-23.3%	-39.5%	-2.6%	1.01%	0.86%	1.19%	1	0	1	174,626	59,283
Saint Vincent and the Grenadines	1.41	1.18	1.65	1.17	0.93	1.44	-17.1%	-35.1%	5.3%	1.21%	1.02%	1.41%	1	0	1	113,144	43,137
Suriname	0.86	0.55	1.08	0.79	0.60	1.01	-8.5%	-33.8%	50.3%	0.5%	0.4%	0.6%	2	2	3	575,888	238,769
Trinidad and Tobago	0.62	0.54	0.70	0.56	0.41	0.75	-10.2%	-34.8%	22.1%	0.55%	0.47%	0.65%	3	2	4	1,387,457	449,574
Virgin Islands	0.59	0.47	0.73	0.34	0.25	0.47	-42.1%	-60.2%	-15.0%	0.56%	0.46%	0.69%	0	0	0	103,985	32,273
**Central Latin America**	**0.74**	**0.61**	**0.82**	**0.58**	**0.48**	**0.70**	**-20.9%**	**-33.2%**	**-5.6%**	**0.53%**	**0.45%**	**0.64%**	**682**	**565**	**824**	**250,020,433**	**109,152,395**
Colombia	0.59	0.48	0.67	0.27	0.20	0.35	-54.4%	-66.9%	-36.2%	0.25%	0.22%	0.30%	55	42	73	47,776,679	19,133,887
Costa Rica	0.19	0.17	0.23	0.15	0.11	0.20	-21.8%	-42.7%	5.2%	0.25%	0.20%	0.31%	3	2	4	4,716,744	1,813,168
El Salvador	0.48	0.41	0.57	0.49	0.34	0.68	1.5%	-27.5%	40.1%	0.49%	0.39%	0.63%	16	11	22	6,256,143	2,909,122
Guatemala	0.48	0.38	0.66	0.89	0.66	1.15	84.8%	18.1%	158.2%	0.54%	0.46%	0.65%	90	67	116	17,776,490	9,585,012
Honduras	0.57	0.39	0.74	0.31	0.19	0.47	-46.1%	-65.6%	-19.6%	0.26%	0.18%	0.35%	17	11	25	9,814,396	5,359,934
Mexico	0.94	0.75	1.07	0.71	0.59	0.88	-24.4%	-36.8%	-6.8%	0.71%	0.58%	0.90%	412	339	513	124,940,175	53,919,125
Nicaragua	0.52	0.44	0.61	0.44	0.33	0.59	-16.0%	-37.7%	11.7%	0.5%	0.4%	0.6%	15	11	20	6,510,365	3,242,972
Panama	0.25	0.21	0.32	0.31	0.23	0.42	25.5%	-9.4%	70.3%	0.31%	0.26%	0.37%	6	4	8	4,160,457	1,847,162
Venezuela	0.48	0.43	0.53	0.59	0.44	0.78	24.7%	-8.0%	66.3%	0.43%	0.37%	0.50%	69	52	91	28,068,986	11,342,013
**Tropical Latin America**	**0.73**	**0.63**	**0.81**	**0.42**	**0.38**	**0.46**	**-42.9%**	**-49.0%**	**-35.3%**	**0.3%**	**0.3%**	**0.4%**	**378**	**343**	**418**	**223,595,269**	**84,921,040**
Brazil	0.75	0.64	0.83	0.42	0.38	0.47	-43.6%	-49.8%	-36.5%	0.3%	0.3%	0.4%	366	332	402	216,664,814	81,666,427
Paraguay	0.30	0.24	0.37	0.37	0.26	0.51	24.7%	-11.3%	73.8%	0.38%	0.31%	0.48%	13	9	17	6,930,455	3,254,612
**North Africa and Middle East**	**0.59**	**0.50**	**0.69**	**0.37**	**0.29**	**0.47**	**-37.2%**	**-49.7%**	**-21.0%**	**0.24%**	**0.20%**	**0.28%**	**1044**	**827**	**1311**	**608,713,645**	**280,522,846**
**North Africa and Middle East**	**0.59**	**0.50**	**0.69**	**0.37**	**0.29**	**0.47**	**-37.2%**	**-49.7%**	**-21.0%**	**0.24%**	**0.20%**	**0.28%**	**1044**	**827**	**1311**	**608,713,645**	**280,522,846**
Afghanistan	1.05	0.66	1.49	0.81	0.46	1.22	-23.0%	-45.3%	10.9%	0.19%	0.11%	0.28%	200	111	306	38,277,536	25,364,922
Algeria	0.46	0.33	0.68	0.27	0.20	0.39	-41.7%	-59.2%	-19.7%	0.20%	0.16%	0.29%	49	36	71	41,847,290	18,016,342
Bahrain	0.86	0.66	1.15	0.70	0.54	0.88	-18.2%	-41.5%	17.5%	1.27%	1.04%	1.50%	3	2	3	1,442,691	400,130
Egypt	0.65	0.55	0.81	0.43	0.30	0.60	-34.0%	-54.3%	-6.5%	0.39%	0.31%	0.49%	215	151	298	99,069,551	50,667,720
Iran	0.31	0.26	0.39	0.26	0.20	0.30	-16.2%	-39.0%	4.2%	0.28%	0.22%	0.33%	82	64	94	84,297,882	31,657,812
Iraq	1.19	0.90	1.52	0.55	0.41	0.72	-53.6%	-66.7%	-33.3%	0.48%	0.38%	0.62%	130	96	171	42,119,490	22,522,304
Jordan	0.38	0.30	0.48	0.18	0.14	0.24	-52.7%	-65.6%	-34.8%	0.21%	0.17%	0.27%	11	9	14	11,636,717	6,006,184
Kuwait	1.12	0.87	1.41	0.45	0.34	0.62	-59.6%	-71.2%	-44.6%	0.73%	0.57%	0.94%	6	5	9	4,426,561	1,394,787

Lebanon	0.33	0.23	0.45	0.19	0.13	0.26	-43.4%	-61.4%	-17.5%	0.23%	0.17%	0.32%	4	3	5	5,177,069	2,081,516
Libya	0.34	0.24	0.49	0.23	0.17	0.32	-31.6%	-52.4%	-2.8%	0.24%	0.19%	0.32%	6	5	9	6,735,543	2,642,247
Morocco	0.42	0.30	0.62	0.29	0.19	0.45	-29.9%	-55.3%	6.1%	0.26%	0.19%	0.37%	45	30	68	35,952,186	15,526,108
Oman	0.65	0.46	0.95	0.42	0.33	0.53	-36.3%	-58.8%	-3.4%	0.44%	0.36%	0.56%	7	6	9	4,583,999	1,695,947
Palestine	0.53	0.37	0.70	0.33	0.26	0.41	-37.9%	-55.4%	-11.1%	0.39%	0.31%	0.47%	9	7	11	4,956,597	2,872,981
Qatar	0.64	0.47	0.89	0.25	0.19	0.34	-60.2%	-74.2%	-41.8%	0.37%	0.30%	0.46%	2	1	3	2,864,548	801,116
Saudi Arabia	0.53	0.36	0.74	0.23	0.17	0.31	-56.8%	-71.0%	-37.4%	0.42%	0.33%	0.53%	35	26	47	35,731,972	12,934,117
Sudan	0.44	0.28	0.68	0.32	0.20	0.48	-26.2%	-52.5%	18.3%	0.12%	0.08%	0.17%	78	48	117	40,808,425	24,226,560
Syria	0.82	0.64	1.05	0.43	0.33	0.57	-47.6%	-62.3%	-26.9%	0.49%	0.41%	0.59%	33	25	44	14,491,247	7,247,391
Tunisia	0.35	0.25	0.57	0.17	0.11	0.25	-50.7%	-70.2%	-26.4%	0.23%	0.17%	0.31%	7	5	11	11,571,604	4,259,173
Turkey	0.88	0.65	1.24	0.24	0.19	0.31	-73.1%	-82.8%	-60.9%	0.3%	0.2%	0.4%	68	54	85	81,359,693	29,055,842
United Arab Emirates	0.20	0.14	0.29	0.15	0.10	0.22	-23.8%	-48.5%	8.8%	0.3%	0.2%	0.4%	3	2	4	9,241,704	1,834,860
Yemen	0.29	0.14	0.49	0.27	0.13	0.42	-8.0%	-45.8%	56.4%	0.08%	0.04%	0.12%	50	24	80	31,502,896	19,029,780
**South Asia**	**0.55**	**0.45**	**0.66**	**0.49**	**0.41**	**0.58**	**-10.6%**	**-29.4%**	**12.2%**	**0.23%**	**0.19%**	**0.27%**	**4409**	**3729**	**5189**	**1,805,200,296**	**862,873,366**
**South Asia**	**0.55**	**0.45**	**0.66**	**0.49**	**0.41**	**0.58**	**-10.6%**	**-29.4%**	**12.2%**	**0.23%**	**0.19%**	**0.27%**	**4409**	**3729**	**5189**	**1,805,200,296**	**862,873,366**
Bangladesh	1.41	0.90	2.01	1.12	0.71	1.49	-20.5%	-47.5%	22.1%	0.70%	0.45%	0.91%	834	536	1110	159,259,850	73,979,413
Bhutan	0.41	0.18	0.61	0.39	0.25	0.60	-2.8%	-44.8%	129.9%	0.24%	0.17%	0.33%	1	1	2	754,250	341,872
India	0.43	0.36	0.50	0.32	0.27	0.39	-24.2%	-40.0%	-4.0%	0.2%	0.1%	0.2%	2214	1814	2647	1,390,706,968	639,986,958
Nepal	0.65	0.42	0.93	0.53	0.38	0.69	-18.5%	-48.4%	20.7%	0.3%	0.2%	0.4%	84	61	110	30,416,382	15,572,923
Pakistan	0.52	0.39	0.66	1.00	0.74	1.26	91.4%	41.1%	146.0%	0.24%	0.17%	0.30%	1275	946	1613	224,062,847	132,992,199
**Southeast Asia, east Asia, and Oceania**	**0.74**	**0.54**	**0.90**	**0.53**	**0.43**	**0.65**	**-28.3%**	**-39.5%**	**-8.9%**	**0.6%**	**0.5%**	**0.7%**	**3939**	**3186**	**4905**	**2,159,261,972**	**684,738,239**
**East Asia**	**0.43**	**0.34**	**0.53**	**0.18**	**0.15**	**0.22**	**-58.5%**	**-65.5%**	**-49.0%**	**0.31%**	**0.25%**	**0.39%**	**777**	**629**	**975**	**1,472,203,526**	**396,315,884**
China	0.43	0.34	0.53	0.17	0.14	0.21	-59.6%	-66.8%	-49.9%	0.3%	0.2%	0.4%	722	579	909	1,422,350,422	381,781,422
North Korea	0.67	0.41	0.99	0.45	0.31	0.61	-33.3%	-53.9%	1.4%	0.50%	0.37%	0.68%	46	31	63	26,232,861	8,742,217
Taiwan (province of China)	0.30	0.24	0.37	0.14	0.10	0.18	-54.6%	-65.3%	-39.9%	0.43%	0.36%	0.52%	10	7	13	23,620,243	5,792,246
Oceania	1.56	1.16	2.05	1.80	1.36	2.35	15.3%	-13.7%	55.8%	0.50%	0.40%	0.61%	130	98	170	13,276,441	7,328,956
American Samoa	0.77	0.59	1.02	1.07	0.73	1.58	38.1%	-2.0%	98.6%	1.26%	0.95%	1.66%	0	0	0	55,505	27,707
Cook Islands	0.98	0.73	1.32	0.64	0.42	0.91	-34.4%	-58.4%	2.0%	1.66%	1.24%	2.11%	0	0	0	17,987	6,852
Fiji	1.81	1.36	2.34	2.17	1.61	2.92	19.6%	-16.9%	72.0%	1.40%	1.16%	1.69%	9	7	12	911,248	416,386
Guam	0.40	0.31	0.53	0.48	0.33	0.67	20.0%	-11.0%	60.5%	0.49%	0.35%	0.68%	0	0	1	170,628	72,775
Kiribati	2.23	1.67	2.90	2.68	1.97	3.50	20.1%	-14.2%	63.8%	0.96%	0.71%	1.27%	2	1	2	118,621	63,572
Marshall Islands	1.46	1.04	1.95	1.54	1.04	2.18	4.9%	-25.1%	46.4%	0.92%	0.67%	1.20%	0	0	1	56,842	28,760
Federated States of Micronesia	1.58	1.14	2.13	2.06	1.34	2.96	30.4%	-12.7%	85.1%	1.52%	1.03%	2.10%	1	1	2	102,116	52,126
Nauru	1.68	1.24	2.18	2.04	1.46	2.77	21.5%	-11.4%	62.6%	0.95%	0.71%	1.23%	0	0	0	10,551	6,039
Niue	1.19	0.83	1.65	1.35	0.91	1.99	12.9%	-24.2%	71.5%	1.04%	0.75%	1.38%	0	0	0	1,672	653
Northern Mariana Islands	0.58	0.39	0.84	0.63	0.43	0.90	8.5%	-20.7%	54.2%	0.9%	0.6%	1.2%	0	0	0	42,494	15,089

Palau	1.08	0.72	1.59	1.37	0.94	1.87	26.4%	-13.1%	87.2%	1.20%	0.86%	1.61%	0	0	0	18,007	5,694
Papua New Guinea	1.58	1.10	2.19	1.78	1.29	2.39	13.1%	-22.1%	67.1%	0.44%	0.33%	0.55%	98	71	132	9,866,614	5,560,121
Samoa	1.07	0.77	1.51	1.00	0.62	1.44	-7.2%	-45.3%	45.9%	1.00%	0.72%	1.29%	1	1	2	211,354	115,810
Solomon Islands	2.21	1.52	3.11	2.50	1.81	3.32	13.3%	-21.5%	62.9%	0.94%	0.72%	1.22%	9	6	12	655,632	383,886
Tokelau	1.05	0.73	1.47	1.03	0.67	1.52	-1.5%	-35.8%	48.5%	1.17%	0.87%	1.54%	0	0	0	1,411	694
Tonga	0.66	0.49	0.86	0.87	0.59	1.28	32.7%	-8.4%	96.9%	0.85%	0.66%	1.17%	0	0	1	102,350	55,125
Tuvalu	1.72	1.20	2.36	1.34	0.91	1.94	-21.9%	-51.0%	16.9%	1.15%	0.84%	1.51%	0	0	0	11,798	5,645
Vanuatu	0.80	0.53	1.17	1.19	0.75	1.64	47.4%	-0.1%	117.0%	0.59%	0.41%	0.78%	2	1	3	294,550	165,867
**Southeast Asia**	**1.51**	**1.06**	**1.86**	**1.01**	**0.80**	**1.26**	**-33.5%**	**-45.2%**	**-12.1%**	**0.75%**	**0.61%**	**0.93%**	**3032**	**2397**	**3814**	**673,782,005**	**281,093,399**
Cambodia	1.95	1.08	2.78	0.95	0.65	1.27	-51.1%	-66.2%	-27.0%	0.48%	0.34%	0.63%	78	54	104	16,603,118	8,026,657
Indonesia	1.21	0.94	1.55	1.00	0.77	1.31	-17.3%	-32.5%	3.3%	0.77%	0.59%	1.01%	1217	928	1591	259,465,835	109,633,938
Laos	2.49	1.47	3.47	1.24	0.82	1.66	-50.2%	-66.7%	-25.2%	0.49%	0.36%	0.64%	47	31	63	7,158,250	3,644,482
Malaysia	0.59	0.46	0.76	0.32	0.22	0.44	-46.7%	-62.3%	-26.9%	0.54%	0.41%	0.71%	48	34	67	31,301,402	13,299,244
Maldives	0.78	0.55	1.07	0.30	0.22	0.41	-61.1%	-71.5%	-47.5%	0.3%	0.2%	0.5%	1	0	1	498,414	179,981
Mauritius	0.67	0.52	0.84	1.31	0.98	1.74	97.1%	51.0%	151.2%	2.0%	1.6%	2.5%	6	5	9	1,276,663	402,349
Myanmar	2.62	1.86	3.58	1.93	1.30	2.68	-26.4%	-52.3%	13.8%	0.82%	0.65%	1.02%	487	330	675	54,676,901	24,598,054
Philippines	3.38	1.83	4.31	1.35	1.07	1.70	-60.0%	-71.1%	-21.3%	0.84%	0.66%	1.07%	775	612	977	112,142,764	56,494,842
Seychelles	0.36	0.26	0.49	0.50	0.34	0.67	37.5%	8.8%	73.2%	0.59%	0.42%	0.80%	0	0	0	102,145	36,089
Sri Lanka	0.61	0.45	0.81	0.55	0.38	0.77	-9.7%	-36.7%	25.8%	0.84%	0.64%	1.07%	50	34	70	21,854,452	8,431,991
Thailand	0.82	0.56	1.18	0.38	0.26	0.55	-53.2%	-67.7%	-33.5%	0.58%	0.42%	0.76%	97	65	139	70,111,586	19,845,116
Timor-Leste	1.14	0.51	1.84	0.75	0.38	1.04	-34.7%	-66.8%	32.8%	0.36%	0.17%	0.50%	6	3	8	1,334,823	796,596
Vietnam	0.64	0.40	0.94	0.57	0.40	0.80	-10.9%	-40.7%	31.7%	0.7%	0.5%	0.9%	216	150	307	96,372,928	35,335,799
**Sub-Saharan Africa**	**0.87**	**0.72**	**1.03**	**0.72**	**0.59**	**0.89**	**-16.6%**	**-31.8%**	**1.3%**	**0.15%**	**0.12%**	**0.17%**	**4749**	**3833**	**5883**	**1,078,209,307**	**672,906,964**
**Central sub-Saharan Africa**	**1.28**	**0.99**	**1.63**	**0.89**	**0.65**	**1.21**	**-30.0%**	**-50.1%**	**-3.0%**	**0.22%**	**0.16%**	**0.29%**	**723**	**520**	**1009**	**131,544,552**	**83,078,127**
Angola	1.11	0.79	1.50	0.87	0.60	1.17	-21.3%	-50.6%	19.9%	0.22%	0.16%	0.28%	162	110	225	30,138,521	19,617,230
Central African Republic	1.47	1.10	1.89	1.24	0.87	1.81	-15.5%	-42.5%	22.0%	0.14%	0.10%	0.18%	41	29	60	5,299,863	3,323,576
Congo (Brazzaville)	1.33	0.97	1.79	0.75	0.52	1.07	-43.7%	-63.1%	-13.8%	0.25%	0.19%	0.35%	21	14	30	5,265,846	2,936,697
DR Congo	1.30	0.95	1.75	0.89	0.61	1.32	-31.6%	-56.8%	4.2%	0.22%	0.15%	0.33%	484	321	735	87,670,444	55,369,372
Equatorial Guinea	1.21	0.77	1.71	0.68	0.38	1.08	-44.1%	-71.2%	6.2%	0.27%	0.18%	0.39%	6	3	10	1,419,839	903,811
Gabon	1.24	0.87	1.67	0.86	0.57	1.25	-30.8%	-57.8%	8.4%	0.42%	0.27%	0.59%	8	5	12	1,750,038	927,441
**Eastern sub-Saharan Africa**	**1.04**	**0.88**	**1.22**	**0.81**	**0.66**	**0.99**	**-22.7%**	**-37.7%**	**-3.8%**	**0.20%**	**0.17%**	**0.23%**	**2080**	**1704**	**2556**	**411,777,253**	**262,637,723**
Burundi	1.20	0.89	1.61	0.79	0.54	1.09	-34.6%	-58.9%	0.6%	0.15%	0.11%	0.22%	57	38	83	11,934,361	7,700,575

Comoros	0.77	0.18	1.22	0.68	0.38	0.98	-10.8%	-51.4%	284.4%	0.23%	0.12%	0.32%	3	1	4	714,351	370,649
Djibouti	0.74	0.50	1.06	0.79	0.48	1.17	6.5%	-36.9%	72.6%	0.23%	0.16%	0.31%	5	3	7	1,202,797	626,215
Eritrea	0.87	0.56	1.30	0.86	0.56	1.26	-1.0%	-38.1%	70.9%	0.26%	0.20%	0.34%	34	23	50	6,711,213	4,070,481
Ethiopia	1.41	1.15	1.77	0.68	0.54	0.85	-52.2%	-64.8%	-35.8%	0.20%	0.16%	0.24%	460	368	579	107,591,164	69,237,043
Kenya	0.47	0.39	0.56	0.48	0.39	0.59	2.6%	-18.4%	29.1%	0.18%	0.15%	0.22%	142	114	174	50,227,709	29,984,138
Madagascar	1.04	0.82	1.32	0.60	0.44	0.79	-42.4%	-59.8%	-18.7%	0.2%	0.1%	0.2%	95	69	125	26,690,344	16,411,490
Malawi	1.11	0.79	1.52	0.83	0.59	1.14	-25.5%	-51.4%	17.7%	0.22%	0.16%	0.28%	93	66	127	18,442,238	11,908,993
Mozambique	0.97	0.68	1.38	1.20	0.82	1.72	22.9%	-20.1%	85.5%	0.22%	0.15%	0.30%	228	155	329	29,528,037	19,700,343
Rwanda	1.17	0.89	1.52	0.72	0.50	1.00	-38.7%	-60.9%	-9.6%	0.25%	0.18%	0.32%	52	37	73	12,688,117	7,468,447
Somalia	1.07	0.74	1.49	1.13	0.81	1.62	5.2%	-28.9%	58.3%	0.16%	0.12%	0.21%	154	108	223	20,343,112	13,685,055
South Sudan	0.78	0.51	1.09	0.76	0.49	1.17	-2.6%	-36.1%	49.7%	0.12%	0.08%	0.18%	47	30	73	9,282,963	6,092,060
Uganda	0.64	0.44	0.88	0.74	0.50	1.05	16.4%	-24.4%	82.9%	0.17%	0.12%	0.23%	198	132	278	41,117,856	27,803,238
Tanzania	1.13	0.84	1.50	1.09	0.76	1.51	-3.2%	-36.1%	50.1%	0.3%	0.2%	0.4%	407	277	577	56,736,116	35,803,751
Zambia	1.18	0.87	1.54	0.93	0.68	1.22	-20.7%	-46.5%	14.8%	0.24%	0.19%	0.31%	104	76	137	18,237,683	11,565,281
**Southern sub-Saharan Africa**	**0.59**	**0.51**	**0.70**	**0.62**	**0.51**	**0.76**	**6.1%**	**-13.3%**	**29.3%**	**0.21%**	**0.17%**	**0.24%**	**236**	**194**	**286**	**78,574,818**	**37,422,996**
Botswana	0.57	0.43	0.75	0.70	0.45	1.03	22.8%	-21.6%	84.3%	0.25%	0.17%	0.35%	8	5	12	2,338,721	1,126,065
eSwatini	0.73	0.57	0.91	1.16	0.78	1.68	59.3%	3.6%	133.7%	0.31%	0.23%	0.42%	7	5	11	1,142,109	644,207
Lesotho	0.53	0.39	0.70	0.94	0.68	1.25	77.1%	17.2%	162.3%	0.20%	0.15%	0.26%	11	8	14	2,091,588	1,088,879
Namibia	0.50	0.30	0.68	0.48	0.29	0.68	-5.6%	-43.4%	77.1%	0.18%	0.12%	0.24%	6	4	9	2,403,124	1,307,244
South Africa	0.58	0.50	0.69	0.43	0.34	0.53	-25.5%	-42.1%	-4.7%	0.17%	0.13%	0.21%	108	85	134	55,588,425	24,236,755
Zimbabwe	0.62	0.44	0.83	1.08	0.79	1.45	73.4%	19.9%	165.4%	0.28%	0.20%	0.36%	95	70	128	15,010,852	9,019,845
**Western sub-Saharan Africa**	**0.64**	**0.48**	**0.86**	**0.61**	**0.45**	**0.82**	**-3.9%**	**-25.4%**	**21.0%**	**0.10%**	**0.07%**	**0.13%**	**1711**	**1239**	**2332**	**456,312,684**	**289,768,118**
Benin	0.79	0.54	1.16	0.74	0.44	1.15	-5.5%	-41.7%	46.7%	0.12%	0.08%	0.17%	59	34	93	12,665,751	8,260,129
Burkina Faso	0.73	0.44	1.12	0.89	0.45	1.54	20.6%	-23.8%	74.5%	0.11%	0.06%	0.20%	129	64	239	22,691,773	14,714,346
Cape Verde	0.36	0.26	0.48	0.38	0.28	0.49	5.3%	-27.1%	42.8%	0.33%	0.24%	0.43%	1	1	1	563,563	260,386
Cameroon	0.72	0.53	0.96	0.86	0.53	1.25	18.0%	-25.9%	73.7%	0.18%	0.12%	0.24%	146	90	215	29,101,868	17,971,067
Chad	0.64	0.47	0.85	0.74	0.49	1.00	15.1%	-22.4%	65.7%	0.08%	0.06%	0.11%	79	53	108	16,398,860	11,450,169
Côte d'Ivoire	0.76	0.50	1.09	0.73	0.45	1.07	-3.7%	-40.3%	46.4%	0.14%	0.09%	0.20%	109	66	163	26,171,532	15,666,131
The Gambia	0.56	0.33	0.84	0.60	0.39	0.83	7.4%	-34.6%	72.8%	0.23%	0.15%	0.32%	8	5	11	2,245,866	1,403,270
Ghana	0.60	0.43	0.86	0.66	0.41	1.00	11.0%	-28.1%	58.0%	0.20%	0.13%	0.29%	117	73	176	31,536,232	17,673,631
Guinea	0.93	0.61	1.46	0.94	0.64	1.34	0.2%	-32.2%	51.2%	0.13%	0.10%	0.19%	73	48	109	12,643,149	8,137,345
Guinea-Bissau	1.11	0.78	1.53	0.96	0.67	1.30	-14.1%	-38.6%	20.5%	0.19%	0.13%	0.25%	11	7	15	1,901,191	1,183,680
Liberia	0.86	0.57	1.29	0.64	0.42	0.89	-25.1%	-52.3%	15.7%	0.17%	0.11%	0.23%	18	12	25	4,789,907	2,847,774
Mali	0.98	0.64	1.57	0.84	0.48	1.35	-14.7%	-42.1%	27.1%	0.10%	0.06%	0.15%	122	67	206	21,917,467	14,748,167

Mauritania	0.65	0.49	0.82	0.43	0.25	0.67	-33.6%	-61.4%	1.4%	0.16%	0.11%	0.22%	10	6	16	4,014,273	2,432,564
Niger	0.73	0.51	1.00	0.65	0.41	0.96	-11.3%	-41.9%	34.7%	0.08%	0.05%	0.10%	102	62	156	23,295,353	16,534,667
Nigeria	0.50	0.37	0.68	0.44	0.32	0.60	-11.5%	-36.1%	23.5%	0.07%	0.05%	0.09%	592	424	797	214,823,786	137,424,367
São Tomé and Príncipe	0.51	0.31	0.77	0.43	0.28	0.60	-16.4%	-44.4%	34.0%	0.27%	0.20%	0.36%	0	0	1	205,385	116,144
Senegal	0.82	0.54	1.22	0.71	0.45	1.01	-13.9%	-47.1%	36.7%	0.22%	0.14%	0.30%	63	40	91	15,134,067	9,195,887
Sierra Leone	0.73	0.43	1.18	0.82	0.50	1.24	12.6%	-30.0%	78.4%	0.12%	0.08%	0.18%	42	25	64	8,284,755	5,085,493
Togo	0.68	0.46	1.02	0.63	0.39	0.96	-7.4%	-37.8%	29.2%	0.15%	0.10%	0.22%	28	17	43	7,921,527	4,658,850

Supplementary Figure 1 – Map showing SDI quintile for each country.

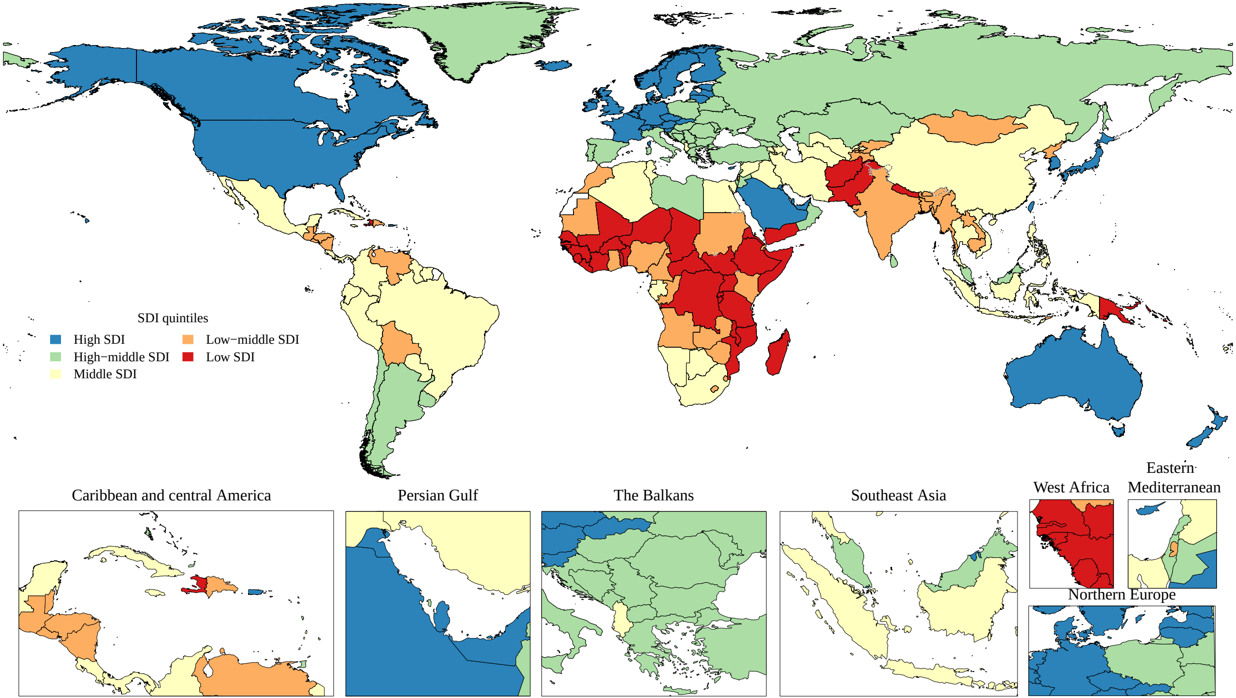

Supplementary Figure 2A: Diabetes (type 1 and type 2 combined) mortality rates under age 25 by Socio-demographic Index (SDI) groups, Trends in age-standardised mortality rate from 1990 to 2019:

A

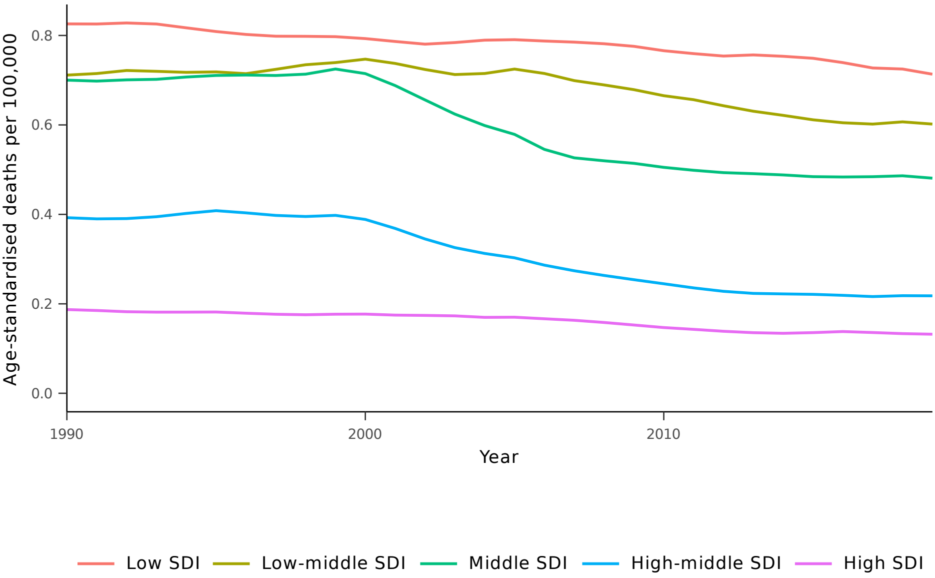

Supplementary Figure 2B: Diabetes (type 1 and type 2 combined) mortality rates under age 25 by Socio-demographic Index (SDI) groups, Age-specific mortality in 2019

B

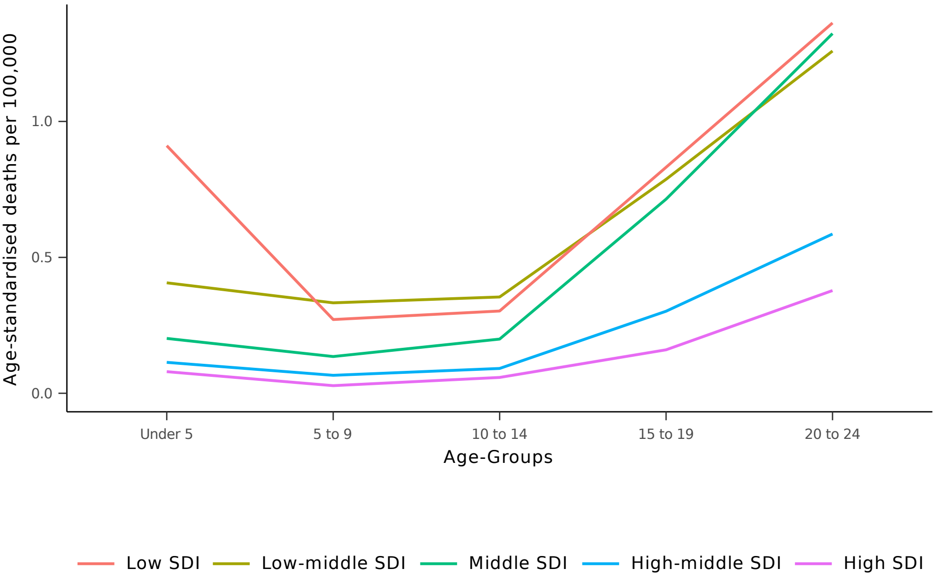

Supplementary Figure 3: Age-standardised mortality rate (per 100,000) due to type 1 diabetes under age 25 in 2019. Countries with total population <1 million are excluded.

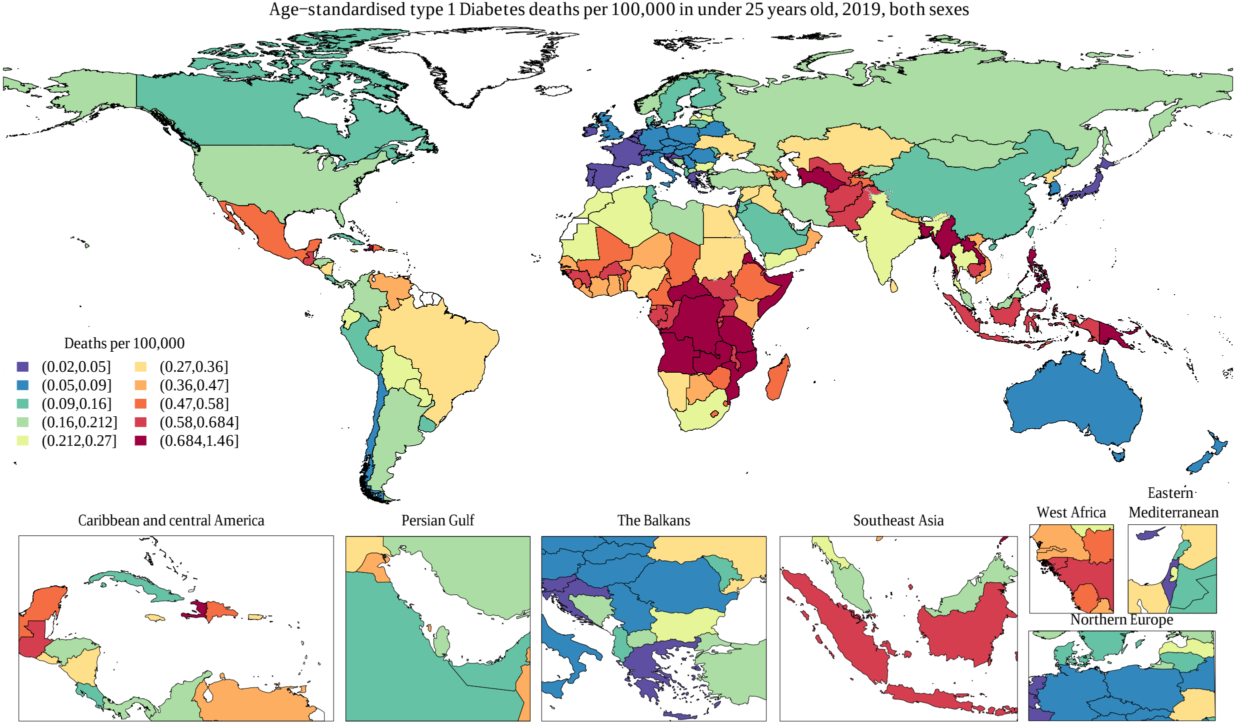

Supplement: Supplementary appendix [file mmc1.pdf]
